# Allele-Stratified Activity of RAS Inhibitors in Pancreatic Ductal Adenocarcinoma: A Systematic Review and Exploratory Meta-Analysis

**DOI:** 10.3390/cancers18193139

**Published:** 2026-09-28

**Authors:** Munahil Ilyas Muhammad, Aisha Al-Khinji, Mayasa Al-Ansari, Najlaa Al-Marri, Shahad Al-Shaheen, Dhafer Malouche

**Affiliations:** 1 College of Medicine, Qatar University, Doha P.O. Box 2713, Qatar; 2 Department of Mathematics and Statistics, College of Arts and Sciences, Qatar University, Doha P.O. Box 2713, Qatar

**Keywords:** PDAC, KRAS mutations, RAS inhibitors, allele-specific therapy, precision oncology, preclinical model validity, evidence stratification

## Abstract

**Background:** PDAC is a highly aggressive malignancy driven predominantly by activating KRAS mutations, and allele-specific and pan-RAS inhibitors have emerged as candidate targeted therapies. Evidence supporting them is dominated by experimental studies of which the models differ widely in how faithfully they reproduce human disease, and clinical maturity is uneven across alleles. **Objectives:** The objective of this study was to evaluate allele-stratified RAS-inhibitor activity across KRAS subtypes in PDAC, synthesizing experimental and clinical evidence separately, and determine whether the principal conclusions of the review survive when the experimental evidence is stratified by the level of model validation achieved. **Methods:** PubMed, Cochrane Library, Scopus, and Google Scholar were searched from inception to 26 June 2026, with a pre-specified updated search in June 2026. Experimental studies were appraised with a modified JBI Critical Appraisal Checklist for Quasi-Experimental Studies supplemented by preclinical-specific reporting domains, and were independently classified by the highest level of model validation achieved, from conventional two-dimensional cell lines through to multi-model validation spanning patient-derived and immunocompetent in vivo systems; clinical studies were appraised with ROBINS-I. Experimental and clinical evidence was synthesized separately. Single-arm proportions from KRAS G12C trials were pooled on the logit scale using inverse-variance random-effects meta-analysis with Hartung–Knapp confidence intervals, and certainty was rated using GRADE. **Results:** Twenty-four studies were included (twenty experimental and four early-phase, single-arm clinical trials). Four of the twenty experimental studies (20%) rested on conventional two-dimensional cell lines alone, nine (45%) incorporated patient-derived material, seven (35%) used immunocompetent models, and fourteen (70%) reproduced their principal finding in at least two independent model classes. The allele-dependent pattern of single-agent activity persisted when cell-line-only studies were set aside, whereas 6 of the 17 studies reporting combination strategies provided no confirmation in any higher-order system. Across three single-arm KRAS G12C trials, the pooled objective response rate was 36.9% (95% CI, 9.8–75.7%; I^2^ = 66.2%; individual trials 21.1%, 45.5%, and 46.9%), the pooled disease control rate was 85.9% (95% CI, 62.1–95.8%; I^2^ = 0%; individual datasets 81.8%, 84.2%, and 93.8%), and the grade ≥ 3 treatment-related adverse event rate was 23.8% (95% CI, 11.2–43.5%; I^2^ = 0%). GRADE certainty was very low for all three outcomes. **Conclusions:** The available evidence is consistent with allele-dependent activity of RAS-targeted therapy in PDAC and supports biomarker-guided treatment selection, but the clinical evidence base is confined to small, single-arm, early-phase trials of KRAS G12C inhibitors. The pooled estimates are exploratory descriptions of observed activity in selected early-phase populations, not comparative estimates of treatment efficacy. KRAS G12D-directed therapy remains at a substantially earlier stage of clinical validation than KRAS G12C-directed therapy despite a much larger preclinical literature.

## Simple Summary

Pancreatic cancer is one of the deadliest cancers, and most cases are driven by mutations in a gene called KRAS. New drugs have been developed to switch off specific mutated forms of KRAS, but it is unclear whether they work equally well against the different KRAS mutations found in pancreatic tumors. We systematically gathered 24 studies: 20 laboratory studies and 4 small early-stage clinical trials. Because laboratory and clinical studies answer different questions, we analyzed them separately, and we additionally graded each laboratory study according to how closely its models resemble human pancreatic cancer: from simple cell cultures through to tumors grown from patients’ own tissue, and to mice with intact immune systems. Drugs targeting the G12C mutation have the most developed clinical evidence and produced the most consistent tumor control. Drugs targeting the more common G12D mutation have, so far, been tested mainly in the laboratory and in a single early trial, so the two are not at comparable stages of clinical validation. Combining these drugs with other treatments enhanced activity and delayed the emergence of resistance in selected laboratory models, but for about a third of the combinations, that finding was never checked in a more lifelike model, and for a quarter of them, it rests on simple cell cultures alone; confirmation in patients is still lacking. These findings support choosing therapy according to a tumor’s specific KRAS mutation, and show where clinical trials are most needed.

## 1. Introduction

PDAC remains a leading cause of cancer-related mortality worldwide and is associated with poor clinical outcomes despite advances in systemic therapy [1]. Most patients with PDAC who are diagnosed with unresectable or metastatic disease have poor long-term survival [2,3]. Clinical outcomes in PDAC remain poor despite the use of chemotherapy and combination treatment regimens [4]. These limitations have increased interest in molecularly targeted therapeutic approaches. Activating KRAS mutations occur in approximately 90–95% of PDAC tumors and represent a major molecular target in PDAC [5].

The high prevalence of KRAS mutations in PDAC has led to the development of therapies that directly target mutant KRAS [6,7]. Allele-specific inhibitors targeting KRAS G12D and KRAS G12C, together with broader RAS-targeted agents, are being evaluated in PDAC [8]. These therapeutic approaches are designed to selectively inhibit mutant KRAS signaling while limiting effects on normal cells [9]. Early clinical studies have reported objective responses and disease control with these therapies. Several KRAS-targeted agents are currently being evaluated across preclinical studies and early-phase clinical trials [10]. However, treatment outcomes may vary across KRAS mutation subtypes and therapeutic approaches [5].

The clinical efficacy of RAS inhibitors is influenced by the specific KRAS allele targeted, with responses varying across different mutation subtypes [11]. KRAS G12D, G12V, and G12R are the most common KRAS mutations in PDAC, whereas KRAS G12C occurs less frequently [12]. Therapies targeting specific KRAS mutations, including KRAS G12D and KRAS G12C, are being evaluated in PDAC. Clinical studies have reported differences in treatment outcomes across KRAS alleles [8,13]. However, these findings are derived from studies evaluating different therapeutic agents, treatment regimens, and patient populations [11,13]. The available evidence, therefore, remains difficult to interpret when comparing the efficacy of RAS inhibitors across individual KRAS mutation subtypes [14].

Allele-specific treatment outcomes are increasingly relevant to the development of biomarker-guided therapies in PDAC [15]. Estimates of mutation-specific response and survival outcomes may improve patient selection and clinical decision-making [13]. Differences in treatment outcomes across KRAS mutation subtypes may also improve the development of future RAS-targeted therapies and clinical trials [11]. Evidence describing the efficacy of RAS inhibitors across individual KRAS mutation subtypes remains limited and dispersed across preclinical and clinical studies [10]. A comprehensive evaluation of the available evidence may improve the interpretation of mutation-specific treatment outcomes across the RAS inhibitor class. This study evaluates the allele-stratified activity of RAS inhibitors in PDAC across KRAS G12D, G12V, G12R, and G12C, as well as broader RAS-mutant populations.

Two features of this evidence base shape the present review and are returned to explicitly in the Discussion. First, the great majority of published data are experimental rather than clinical. Experimental systems in PDAC range from conventional two-dimensional cell lines through three-dimensional spheroids, patient-derived organoids and patient-derived xenografts to genetically engineered, syngeneic and orthotopic immunocompetent models, and these systems differ substantially in the extent to which they reproduce the dense stromal compartment, immune context, vascular supply, intratumoral heterogeneity and drug-exposure characteristics of human PDAC [16,17]. A technically well-conducted experiment in an established cell line may, therefore, possess good internal validity while providing limited evidence about clinical effect. Second, clinical maturity is markedly uneven across alleles: KRAS G12C-directed agents have been evaluated in several early-phase trials, whereas KRAS G12D-directed agents have entered clinical testing only recently despite a far larger preclinical literature. This review is, therefore, designed not only to stratify therapeutic activity by KRAS allele but also to stratify the underlying evidence by the level of model validation on which it rests, so that hypothesis-generating findings can be distinguished from findings with stronger translational support.

## 2. Objectives

The terminology is fixed here before the objectives are stated. Throughout this review, RAS inhibitors denote agents that act on RAS itself, or on the nucleotide exchange interaction that loads it, and comprises the three classes that were eligible as the index intervention: (a) allele-specific KRAS inhibitors and degraders, namely KRAS G12C-directed agents (sotorasib, adagrasib, garsorasib, glecirasib) and KRAS G12D-directed agents (MRTX1133, setidegrasib); (b) multi-selective pan-RAS(ON) inhibitors, represented in the experimental evidence by RMC-7977, the two clinical trials of daraxonrasib having been excluded at the eligibility stage with reasons recorded in Figure 1; and (c) KRAS::SOS1-disrupting agents (BI-3406), which act on a step common to all alleles rather than on a specific one. No pan-KRAS-selective agent met the eligibility criteria. Inhibitors acting downstream of RAS, including MEK, ERK, SHP2, CDK4/6 and BET inhibitors, were not eligible as the index intervention and enter this review only as the combination partners of an eligible RAS-directed agent. Because an agent that is active across RAS G12 variants cannot contribute to a comparison between alleles, pan-RAS agents were eligible for the narrative review but were excluded from the allele-stratified synthesis and from every pooled estimate.

Allele-stratified activity is defined operationally as the activity of an eligible RAS-directed agent reported separately for a named RAS genotype. The alleles considered are KRAS G12C, G12D, G12V and G12R, together with any further RAS genotype, including Q61 variants and RAS wild-type comparators, for which an included study reported an outcome separately. The endpoints differ by evidence stream and are never combined. In the clinical stream, activity is measured by objective response rate, disease control rate, progression-free survival, overall survival, duration of response and clinical benefit rate, with grade ≥ 3 treatment-related adverse events as the safety endpoint. In the experimental stream, it is measured by quantitative potency values (IC50, EC50, or the concentration at which a stated effect was observed), cell viability, proliferation, apoptosis, colony formation and migration, inhibition of target-pathway signaling, tumor growth inhibition or regression, and survival of tumor-bearing animals. Stratification here means that these endpoints are reported and interpreted within allele. The review makes no formal statistical comparison between alleles, and none is available: allele assignment is not randomized or otherwise controlled in any included study, and clinical data exist for only two of the four principal PDAC alleles.

The two evidence streams are ranked rather than merged. Clinical evidence obtained in patients ranks above all experimental evidence for any statement about therapeutic effect; within the experimental stream, evidence is ranked by the level of model validation defined in the next paragraph. Only the clinical stream is synthesized quantitatively, and only where at least two clinically comparable trials of agents of the same class reported the same outcome in the same allele, a condition met by KRAS G12C alone. All experimental evidence, and every clinical outcome that does not meet that condition, was synthesized qualitatively by structured thematic analysis. No experimental result is pooled with, or used in support of, any clinical estimate, and no experimental study contributes to any pooled estimate.

The level of model validation is defined as a six-step ordinal hierarchy of increasing reproduction of the human tumor context, specified in full before any study was classified: (i) conventional two-dimensional cell lines only; (ii) cell lines with three-dimensional spheroid culture; (iii) patient-derived material in vitro, that is, patient-derived primary cultures or organoids, without an in vivo component; (iv) cell-line-derived xenograft, syngeneic or orthotopic in vivo models without patient-derived material; (v) patient-derived xenograft models; and (vi) multi-model validation, defined as at least three complementary systems including both patient-derived material and an in vivo model. This sequence follows the conventional progression from two-dimensional culture through three-dimensional and organoid systems to xenograft, patient-derived and immunocompetent in vivo models. It stops short of clinical evidence by construction: clinical evidence constitutes the separate stream described above and is never treated as the uppermost step of the experimental hierarchy. Each study was assigned the highest step it reached, independently and in duplicate. The full criteria, the duplicate-judgement procedure and the per-study assignments are given under Classification of Experimental Model Validation.

Against these definitions, the main aim of this study was to evaluate the allele-stratified activity of RAS inhibitors in PDAC. The specific objectives were as follows: (i) to synthesize, separately and without merging, the experimental and the clinical evidence for allele-specific and pan-RAS inhibitors in PDAC; (ii) to classify the experimental evidence by the highest level of model validation achieved and to determine whether the principal conclusions of the review survive when evidence derived exclusively from conventional cell lines is considered separately; (iii) to appraise whether the drug concentrations used in vitro are pharmacologically achievable at clinically evaluated doses; and (iv) to derive exploratory pooled estimates of observed activity and toxicity from the available early-phase KRAS G12C trials. Objectives (ii) and (iii) were added in response to peer review and are addressed by post hoc analyses, identified as such in the Methods.

## 3. Methodology

The study adhered to the Preferred Reporting Items for Systematic Reviews and Meta-Analyses (PRISMA) 2020 guidelines [18].

### 3.1. Protocol and Registration

This review was not prospectively registered in PROSPERO. Because the analysis substantially involved preclinical experimental studies, it fell outside the scope of PROSPERO, which registers systematic reviews of health-related outcomes in humans. An a priori internal protocol specifying the PICOS eligibility criteria, the search strategy, the critical appraisal tools and the planned synthesis was prepared and dated before screening began; it was not published in a public register and is available from the corresponding author on request. References elsewhere in this article to a “prespecified” search or analysis refer to that internal protocol and not to a public registration; no claim of registration is made. Two amendments to the protocol were made after screening began. The first was the addition of a pre-specified updated search using a gap-corrected drug block, described under Search Strategy; the second was the admission of the clinical cohort embedded in Dilly et al. to the disease-control pool, described in the Results. Both are recorded in Supplementary File S1 (item 24c). Two further analyses—the classification of experimental model validation and the appraisal of pharmacological achievability—were added in response to peer review. They are post hoc analyses rather than protocol amendments and are identified as such wherever they are described. The review was otherwise conducted and reported in accordance with the PRISMA 2020 guidelines [18]; the completed checklist is provided as Supplementary File S1.

### 3.2. Search Strategy for Identifying Studies

A review author developed the search strings using Medical Subject Headings (MeSH terms) and keywords identified via PubMed. A comprehensive database search was then conducted across PubMed, Cochrane Library, and Scopus. The search was run from each database’s inception to 26 June 2026. Google Scholar was searched using simplified keyword combinations, and the results were screened by relevance; the first 200 results were reviewed, consistent with recommended methodological practice for systematic reviews. Additionally, a manual search of other sources, including clinical trial registries such as ClinicalTrials.gov and the WHO International Clinical Trials Registry Platform, was conducted to identify ongoing or unpublished studies. The citations of the eligible studies were also searched to identify relevant articles. A single pre-specified updated search was then run in June 2026 using a gap-corrected KRAS/RAS drug block, to capture agents that had entered the literature after the original search strings were written; this was a PubMed search with targeted registry verification rather than a full four-database re-run, and it is presented as a distinct identification stream in Figure 1 so that its narrower coverage is transparent. Studies identified by either stream were screened, appraised and counted under identical criteria. The complete search strategies, per-source yields and search dates for both streams are reported in Supplementary File S2.

### 3.3. Study Selection

A review author used Zotero (version 6.0.36) for study selection and screening. All retrieved records were imported, and the software algorithms automatically identified duplicates, which were then manually merged. Titles and abstracts were screened against eligibility criteria, and irrelevant studies were excluded. The remaining articles were then retrieved and evaluated for inclusion by accessing their full texts. Reasons for non-inclusion at the full-text level were recorded. Two reviewers conducted the screening independently and, in cases of disagreement, resolved them through discussion. If consensus was not reached, a third reviewer was consulted. Zotero’s publication-type filters were additionally used to remove records matching the pre-specified excluded publication types (reviews, editorials, letters, comments, conference abstracts and errata) before manual screening; every such exclusion was verified by a reviewer, and the count is reported separately in Figure 1.

### 3.4. Eligibility Criteria

#### 3.4.1. Inclusion Criteria

The study included peer-reviewed original research articles evaluating the activity of RAS inhibitors in PDAC. Eligibility was defined a priori using the PICOS framework [19], applied separately to the clinical and the experimental literature.

**Population (clinical).** Patients with histologically or cytologically confirmed PDAC harboring KRAS or other RAS mutations. **Population (experimental).** Human PDAC-derived experimental systems—established cell lines, primary cultures, spheroids, patient-derived organoids and patient-derived xenografts—and murine PDAC models, including genetically engineered, syngeneic and orthotopic models, with a documented KRAS or RAS genotype.

**Intervention.** RAS-targeted therapies, comprising allele-specific small-molecule inhibitors, degraders and pan-RAS or KRAS::SOS1-directed agents, administered as monotherapy or in combination with other anticancer agents.

**Comparator.** Standard therapy, placebo, alternative targeted therapy, or no comparator where appropriate for single-arm clinical studies; untreated, vehicle-treated or wild-type/non-target-allele controls for experimental studies.

**Outcomes (clinical).** ORR, DCR, PFS, OS, DoR and CBR; secondary outcomes were treatment-related safety, particularly grade ≥ 3 TRAEs. **Outcomes (experimental).** Allele-specific antitumor activity expressed as cell viability, proliferation, apoptosis, colony formation, migration, signaling inhibition, tumor growth or regression, survival of tumor-bearing animals, and mechanisms of therapeutic resistance.

**Study designs.** Early-phase clinical trials and non-randomized clinical studies reporting PDAC-specific outcomes; and in vitro and in vivo experimental studies. Both evidence types were eligible, but they were synthesized separately and are reported separately throughout, because they answer different questions. Experimental studies were included because the allele-specific hypothesis under examination—that therapeutic activity depends on the identity of the KRAS mutation—is currently testable across the full range of PDAC alleles only in preclinical systems: clinical data exist for KRAS G12C and, very recently, for KRAS G12D, whereas G12V and G12R, which together account for a substantial share of PDAC, have no allele-directed clinical evidence at all. Restricting the review to clinical studies would, therefore, have confined it to a single allele and would have made an allele-stratified comparison impossible. No experimental result is used in this review to estimate a clinical effect, and no experimental study contributes to any pooled estimate.

#### 3.4.2. Exclusion Criteria

This study excluded review articles, systematic reviews, meta-analyses, conference abstracts, editorials, commentaries, letters, case reports, study protocols, and non-peer-reviewed publications. Studies not involving PDAC, studies not evaluating KRAS- or RAS-targeted therapies, studies reporting neither a clinical efficacy outcome nor an experimental activity outcome, duplicate publications, and non-English articles were excluded. Publications reporting overlapping patient populations were assessed for cohort overlap, and duplicate data were excluded from quantitative synthesis to avoid the double-counting of participants. Trials of multi-selective (pan-RAS) agents were eligible for the review but were excluded from the allele-stratified synthesis, since an agent active across RAS G12 variants cannot contribute to a comparison between alleles; the exclusions so made are recorded with their reasons in Figure 1.

### 3.5. Risk of Bias Assessment

The methodological quality of the included studies was independently assessed by two reviewers using design-specific tools. Discrepancies between reviewers were resolved through discussion or, where consensus could not be reached, consultation with a third reviewer. No automation tools were used at this stage. Throughout this article, risk-of-bias appraisal is treated as an assessment of internal methodological quality only. It is reported separately from, and must not be read as a substitute for, the assessment of biological model validity and translational relevance described in the following subsection.

The included experimental studies were appraised using a modified JBI Critical Appraisal Checklist for Quasi-Experimental Studies [20]. This tool was selected because the included experimental studies are, in design terms, non-randomized before-and-after or treated-versus-control comparisons without random allocation, which is precisely the design the JBI quasi-experimental checklist was constructed to appraise; because no single validated instrument covers the full range of in vitro, organoid, xenograft and immunocompetent animal designs represented here; and because using one instrument across all experimental designs keeps item-level judgements comparable between studies. The alternative of applying SYRCLE’s risk-of-bias tool [21] to the animal studies and a separate in vitro instrument to the cell-line studies was considered and rejected, because it would have produced two non-comparable sets of judgements across a body of evidence in which most reports combine both designs. The modifications made to the original checklist were confined to the wording of the item anchors, which were re-specified in experimental rather than clinical terms—“participants” was replaced by “experimental models”, “follow-up” by completeness of experimental observation, and “exposure” by intervention implementation—and to the addition of a Not Applicable option for items that cannot apply to purely in vitro designs; no item was deleted and no item was reweighted. The domain anchors as applied are given in full in Table 1. Because the checklist does not interrogate reporting practices specific to preclinical research, three further domains drawn from SYRCLE [21] and the ARRIVE 2.0 guidelines [22] were recorded descriptively for each experimental study alongside the nine JBI items: whether the number of independent biological replicates was stated, whether randomization or blinding of outcome assessment was reported for in vivo experiments, and whether the KRAS genotype of every model used was independently documented. These three domains were considered and noted for transparency; they were not incorporated into the overall JBI judgement, and no numerical weighting was assigned to them, because the checklist has not been validated with additional items and any weighting would have been arbitrary. It must be emphasized that this instrument, like any risk-of-bias instrument, evaluates the internal validity of an experiment and cannot establish that the experimental model adequately represents human PDAC; model validity is assessed separately, as described next.

The risk of bias in the eligible clinical studies was assessed using the ROBINS-I tool [23]. Risk of bias was evaluated across seven domains: confounding (D1), selection of participants (D2), classification of interventions (D3), deviations from intended interventions (D4), missing outcome data (D5), measurement of outcomes (D6), and selection of the reported result (D7). Each domain was judged as low, moderate, serious, critical, or no information according to the highest level of bias identified within that domain, and an overall judgement was assigned accordingly. The domain anchors applied are given in Table 2 and the item-level judgements in Figure 2 and Figure 3. All four included early-phase trials [24,25,26,27] were appraised under ROBINS-I on the same basis. The retrospectively assembled KRAS G12C PDAC cohort embedded in Dilly et al. [28], which contributes to the pooled disease control rate, was appraised separately under ROBINS-I and is displayed alongside the trials in Figure 2 and Figure 3; the full matrix is given in Supplementary File S2, Table 2.

As shown in Table 3, all 20 experimental studies satisfied the modified JBI criteria and were retained. Most studies adequately described the intervention, control groups, outcome measurements, and statistical analyses, although several reported baseline comparability of the experimental models and repeated outcome assessment unclearly. Three further reporting domains specific to preclinical research—whether the number of independent biological replicates was stated, whether randomization or blinding of outcome assessment was reported for in vivo experiments, and whether the KRAS genotype of every model used was independently documented—were considered alongside the JBI items and noted during extraction. They are reported descriptively and were deliberately not folded into the overall judgement, for the reason given in the Methods.

### 3.6. Classification of Experimental Model Validation

Because internal methodological quality and biological model validity are distinct properties, each experimental study was additionally classified, independently and in duplicate, by the highest level of model validation it achieved. This classification, and the appraisal of pharmacological achievability described below, were added in response to peer review; they are post hoc analyses rather than amendments to the a priori protocol, and are identified as such here and under Protocol and Registration. The hierarchy was specified in full before any study was classified and was applied uniformly to all 20 studies. Ordered by increasing reproduction of the human tumor context, it was as follows: (i) conventional two-dimensional cell lines only; (ii) cell lines with three-dimensional spheroid culture; (iii) patient-derived material in vitro (patient-derived primary cultures or patient-derived organoids) without an in vivo component; (iv) cell-line-derived xenograft, syngeneic or orthotopic in vivo models without patient-derived material; (v) patient-derived xenograft models; and (vi) multi-model validation, defined as the use of at least three complementary systems including both patient-derived material and an in vivo model. A second, independent judgement recorded whether the principal finding of each study was reproduced across at least two independent classes of model system (Yes/Partly/No). Disagreements were resolved by discussion with a third reviewer. The classification was applied to the study as a whole and is reported in Section 4.3; the complete per-study matrix, including the model systems on which each judgement rests, is provided in Supplementary File S5. The stratified reanalysis of the principal conclusions of the review by this classification is reported in Section 4.4. Cell-line-only evidence was analysed separately rather than excluded, and that choice is methodological rather than presentational. Excluding it would discard the mechanistic experiments that establish target engagement and allele selectivity in the first place, and would do so unevenly across alleles, because the alleles at the earliest stage of clinical development are precisely those whose literature is most heavily cell-line based; the resulting synthesis would be biased toward the best-resourced allele rather than made more reliable. Separating it instead converts model validity into a sensitivity analysis: each principal conclusion of the review is re-derived from the subset of evidence that reproduces the stromal, immune and pharmacokinetic context absent from monolayer culture, so that a conclusion which survives that restriction is distinguishable from one which does not (Section 4.4). Conventional two-dimensional cell lines cannot reproduce the desmoplastic stroma, the T-cell-excluded microenvironment, the perfusion constraints on drug delivery or the intratumoral heterogeneity that characterize PDAC, and that limitation is the reason the hierarchy exists; its consequences are examined in the Discussion.

### 3.7. Appraisal of Pharmacological Achievability

This appraisal was added in response to peer review and is a post hoc analysis. For every quantitative in vitro potency value reported in the included studies (IC50, EC50, or the concentration at which a stated effect was observed), the tested concentration, the duration of exposure and, where reported, evidence of pharmacodynamic target engagement were extracted. Each value was then compared with the systemic exposure achievable in patients at the licensed or clinically evaluated dose of the same agent, derived from regulatory pharmacokinetic data, with plasma protein binding taken into account. Concentrations were classified as plausibly achievable, of uncertain achievability, or substantially above achievable exposure. Where no human pharmacokinetic data exist for the compound tested—which is the case for the KRAS G12D inhibitor that underpins most of the G12D preclinical literature—achievability was recorded as not assessable rather than assumed. The full appraisal is reported in Supplementary File S5 and summarized in the Discussion.

### 3.8. Data Extraction

Data were extracted independently by two reviewers using a standardized data extraction form. Discrepancies were resolved through discussion, and where consensus could not be reached, a third reviewer was consulted. The following data were extracted from the included studies: study ID, country, study design, sample characteristics, cell lines and other model systems, intervention, target allele, outcome measures, and findings. For experimental studies, the complete inventory of model systems, the drug concentrations and exposure durations tested, the reported potency values, the synergy metric used and its numerical value, and whether each principal finding was reproduced in a higher-order model were extracted in addition, to support the model validation classification and the achievability appraisal described above. No automation tools were used at the extraction stage, and study investigators were not contacted to obtain or confirm data; all values were taken from the published reports. The extraction template is reproduced in Supplementary File S4.

### 3.9. Data Synthesis and Analysis

A structured thematic analysis method was used to synthesize qualitative data. This followed a step-by-step process, including familiarizing with the data, generating initial codes, grouping into possible themes, polishing and justifying the themes, naming each theme, and writing a narrative synthesis [48].

Experimental and clinical evidence were synthesized separately and are reported in separate subsections throughout the Results. No experimental result was pooled with, or used to support, any clinical estimate. Within the experimental evidence, studies were grouped by target KRAS allele and, within allele, by whether the agent was given as monotherapy or in combination; the synthesis was then repeated with the studies stratified by level of model validation, and the conclusions from the two syntheses are compared in Section 4.4. Within the clinical evidence, meta-analyses were performed for outcomes reported by at least two clinically comparable trials evaluating agents of the same class in the same allele. Single-arm proportions (ORR, DCR and grade ≥ 3 TRAEs) were pooled on the logit scale using an inverse-variance random-effects model (DerSimonian–Laird estimator of τ^2^) with Hartung–Knapp adjustment of the confidence intervals, and were back-transformed to percentages with 95% CIs. Individual study proportions with exact (Clopper–Pearson) 95% confidence intervals, and their weights are displayed alongside every pooled estimate in the corresponding forest plot and are stated in the text, so that each pooled value can be read against the trials that produce it. Statistical heterogeneity was quantified using Cochran’s Q test, the Higgins I^2^ statistic and τ^2^. Forest plots were generated to summarize pooled estimates, traffic-light and summary plots to display the ROBINS-I judgements, and leave-one-out sensitivity analyses were performed to evaluate the robustness of pooled results. Analyses were conducted with Comprehensive Meta-Analysis v3 [49], and all pooled estimates were independently reproduced in R 4.5.2 using the meta package (Supplementary File S4). Prediction intervals were pre-specified but are not reported, because with three contributing studies per outcome, they span essentially the whole parameter space. For the same reason, subgroup analysis and meta-regression were not estimable and were not attempted. Time-to-event outcomes were not pooled and are presented as the medians and 95% confidence intervals reported by each trial. The analytic code is provided in Supplementary File S4.

Funnel plots were not produced and no formal test of funnel plot asymmetry was applied. With three studies contributing to each pooled outcome, neither visual inspection nor a statistical test can distinguish asymmetry from chance, and a symmetric-appearing plot would carry no evidential weight; the conventional minimum of ten studies was not approached for any outcome. Publication bias in the clinical literature therefore could not be assessed and is treated as an unresolved limitation rather than as having been excluded. Publication bias in the preclinical literature is discussed separately in the Discussion. Statistical significance was defined as a two-tailed *p*-value < 0.05. Outcomes that were not reported consistently or lacked sufficient quantitative data were synthesized qualitatively. The certainty of the evidence for each quantitatively synthesized outcome was rated using the GRADE approach [50] across the domains of risk of bias, inconsistency, indirectness, imprecision and publication bias; the resulting summary-of-findings table is provided in Supplementary File S3.

## 4. Results

### 4.1. Study Selection and Screening

The original database search identified 275 records from four electronic databases. After removing 3 duplicate records and 62 records excluded by automation tools, 210 records underwent title and abstract screening. Of these, 166 were excluded, leaving 44 full-text reports for eligibility assessment; 21 were excluded at this stage (3 inappropriate study design, 3 overlapping patient populations, 15 reporting irrelevant outcomes), and 23 studies met the eligibility criteria. The pre-specified updated search identified 13 further records; after removal of 2 duplicates of already-included studies, 11 were screened, 6 were excluded and 5 full-text reports were assessed, of which 1 met the eligibility criteria: the KRAS G12C trial of garsorasib [27]. Four reports were excluded at this stage and the reasons are recorded in Figure 1. Two of them were the daraxonrasib trials [51,52]: these were excluded from the synthesis because daraxonrasib is a multi-selective RAS(ON) inhibitor active across RAS G12 variants rather than an allele-selective agent, so it cannot contribute to an allele-stratified comparison, and because pooling a randomized comparison as a single-arm proportion would discard its concurrent control arm; they are, nevertheless, discussed in the Discussion because they materially reframe the field. A third, the D3S-001 study, was excluded because its PDAC subset comprised only 4 patients, below the minimum considered informative; it is used solely in a post hoc sensitivity analysis, reported as such.

Twenty-four studies were therefore included in the review: 20 experimental studies [28,29,30,31,32,33,34,35,36,37,38,39,40,41,42,43,44,45,46,47] and 4 early-phase, single-arm clinical trials [24,25,26,27] (Figure 1). Garsorasib is counted, appraised, tabulated and pooled on exactly the same basis as the other three trials; it is identified as having been retrieved by the updated search only where that provenance is material. One of the experimental reports [28] additionally contained a retrospectively assembled clinical cohort that contributes to the pooled disease control rate; that cohort is a protocol amendment and is appraised and flagged separately wherever it appears, but it does not add to the study count, since the report is already counted among the 20 experimental studies.

### 4.2. Risk of Bias in the Included Studies

The ROBINS-I assessment demonstrated that all four included early-phase clinical trials had an overall moderate risk of bias (Figure 2 and Figure 3). All four studies were judged at low risk of bias in the domains of participant selection (D2), classification of interventions (D3), deviations from intended interventions (D4), missing outcome data (D5), and measurement of outcomes (D6). The judgement for D4 is stated explicitly here because it was omitted from the narrative of the previous version while appearing in the corresponding figure; the two are now harmonized, and D4 is judged low for all four trials, reflecting protocol-defined dosing with dose modifications specified in advance in each report. Moderate risk of bias was observed in the domains of confounding (D1) and selection of the reported result (D7), reflecting the single-arm, non-randomized design of the included early-phase clinical trials [24,25,26,27]. The retrospectively assembled KRAS G12C PDAC cohort embedded in Dilly et al. [28], which contributes to the pooled disease control rate, was rated at serious overall risk of bias: it was not enrolled prospectively, disease control was not defined against a protocol-specified assessment window, and partial overlap with the sotorasib population of CodeBreaK 100 [26] cannot be excluded from the published reports.

These judgements concern internal methodological quality alone. They indicate that the experiments were, on the whole, competently designed, controlled and analyzed; they do not indicate that the models used adequately represent human PDAC, and they should not be read as evidence of translational validity. A carefully controlled experiment in an established PDAC cell line can have good internal validity while having limited external validity, because a monolayer culture reproduces neither the desmoplastic stroma and extracellular matrix that dominate PDAC histology, nor its immune and vascular context, nor its intratumoral heterogeneity, nor the drug-exposure conditions of a treated patient [16,17]. The separate assessment of model validity is reported in the following subsection, and the two assessments are kept distinct throughout.

### 4.3. Overview of Study Characteristics

The four non-randomized, single-arm early-phase clinical trials included were conducted in the United States, China, Japan, Australia and the Republic of Korea, and reported PDAC-specific cohorts of 22 to 38 patients. Because each trial reported a PDAC cohort nested within a broader solid-tumor or multi-cohort study, the PDAC cohort size rather than the parent-trial enrolment is given throughout, and every pooled estimate uses PDAC-specific numerators and denominators, with two documented exceptions, each flagged where it arises. The glecirasib grade ≥ 3 adverse-event denominator is the broader KRAS G12C solid-tumor safety population of that trial rather than its PDAC efficacy subset. In the garsorasib trial the safety and response denominators differ from one another: 24 patients received at least one dose and form the safety population, while 22 were efficacy-evaluable. The trials enrolled patients with advanced or metastatic PDAC harboring KRAS G12C or KRAS G12D mutations who were receiving allele-specific RAS inhibitors. The interventions evaluated were sotorasib, glecirasib, garsorasib and setidegrasib, all administered as monotherapy. Clinical outcomes included ORR, DCR, PFS, OS, DoR and treatment-related safety (Table 4).

The 20 included experimental studies [28,29,30,31,32,33,34,35,36,37,38,39,40,41,42,43,44,45,46,47] were conducted in the United States, China, Germany, Japan, Slovenia, Hungary, India, and Argentina. They evaluated KRAS G12C, G12D, G12V, G12R, Q61H and broader RAS-mutant PDAC using allele-specific, degrader and pan-RAS agents administered as monotherapy or in combination with chemotherapy, immune checkpoint inhibitors, or other targeted therapies. Outcomes were cell viability, proliferation, apoptosis, colony formation, migration, tumor growth and regression, signaling inhibition, survival of tumor-bearing animals, and mechanisms of therapeutic resistance (Table 5).

The model systems used were markedly heterogeneous, and quantifying that heterogeneity is a precondition for interpreting the evidence. Classified by the highest level of model validation achieved, 4 of the 20 studies (20%) [33,36,43,46] used conventional two-dimensional cell lines alone; 1 (5%) [39] added three-dimensional spheroid culture but no in vivo work; 1 (5%) [29] used patient-derived primary cultures without an in vivo component; 6 (30%) [30,32,38,42,45,47] combined cell lines with a cell-line-derived xenograft, syngeneic or orthotopic in vivo model but used no patient-derived material; 1 (5%) [31] extended to patient-derived xenografts; and 7 (35%) [28,34,35,37,40,41,44] achieved multi-model validation, defined as at least three complementary systems including both patient-derived material and an in vivo model. Aggregated differently, 14 of 20 studies (70%) included an in vivo component, 9 (45%) [28,29,31,34,35,37,40,41,44] used patient-derived material of any kind, and 7 (35%) [28,30,34,35,37,41,42] used immunocompetent genetically engineered, syngeneic or orthotopic models in which the stromal and immune contributions to drug response can be observed at all. The principal finding was reproduced across at least two independent classes of model system in 14 studies (70%), partly in 2 (10%) and not at all in 4 (20%). Per-study classifications are given in the final two columns of Table 5, and the model systems underlying each judgement in Supplementary File S5.

### 4.4. Stratified Reanalysis of the Experimental Evidence by Level of Model Validation

The classification above was not applied descriptively. Each principal conclusion of the review was re-examined after the evidence supporting it had been separated into evidence derived exclusively from conventional cell lines and evidence reproduced in patient-derived or in vivo systems; the result is given in Table 6.

Three conclusions were unaffected by the stratification. The allele-selective single-agent activity of KRAS G12D inhibition rests on 12 studies, of which 10 include an in vivo component and 5 use patient-derived material; tumor regression was demonstrated in patient-derived xenografts [31,34,35] and in genetically engineered and orthotopic models [34,37], so the conclusion stands without the cell-line evidence. The corresponding conclusion for KRAS G12C rests on fewer studies but is likewise supported in patient-derived organoids and in vivo [28,41,44]. The observation that sensitivity is heterogeneous within an allele—that homozygous lines respond where heterozygous lines do not [39], and that some models are intrinsically resistant—is reproduced in patient-derived organoids [28,40,44], which is the system in which it matters most, since organoid panels are the closest available surrogate for between-patient variation [16,17].

Two conclusions are materially weakened. The first is the claim that combination strategies enhance activity and delay resistance. Seventeen studies reported at least one combination. In 11 [30,32,34,35,37,38,40,41,42,44,47] the combination was tested in at least one higher-order system—a patient-derived organoid, a xenograft, a patient-derived xenograft or an immunocompetent in vivo model—and in these the conclusion is supported. In the remaining 6 [29,33,36,39,43,46], it was not: of these, one [29] used patient-derived primary cultures and one [39] included three-dimensional spheroids, while four [33,36,43,46] rested on conventional two-dimensional monolayer assays alone. The combinations supported only by monolayer evidence are sotorasib with electrochemotherapy [33], BI-3406 with trametinib or buparlisib [36], sotorasib with camptothecin [43], and MRTX1133 with 5-FU or ONC212 [46]; each is hypothesis-generating and none should be read as evidence of a clinically useful combination. The second conclusion so affected concerns antimigratory activity: it is supported in vivo in a liver metastasis model [45], whereas the scratch-assay evidence [43] is a monolayer observation that does not of itself establish an antimetastatic effect.

One conclusion is available only at the higher tiers. The immune-dependent component of KRAS G12D inhibition—CD8+ T-cell-mediated killing and remodeling of the tumor microenvironment—is observable only in immunocompetent models and is reported in 3 studies [35,37,41]; it has no cell-line-derived counterpart, and its absence from the cell-line literature reflects the limits of that literature rather than a negative result.

Taken together, the stratification leaves the central allele-dependent claim of this review intact while narrowing the combination-therapy claim substantially. It also identifies where the preclinical evidence is thinnest: the KRAS G12V and G12R alleles, which together account for a large share of PDAC, are represented predominantly by cell-line data [36,46], with higher-tier evidence confined to two studies in which G12V models appear within a broader design [31,41]. No conclusion about these alleles rests on more than a single higher-tier study, and none is supported by an immunocompetent model.

### 4.5. Clinical Results

#### 4.5.1. KRAS G12C Treatment Response 

Among 38 patients with advanced PDAC, sotorasib achieved a confirmed partial response rate of 21%, a median time to response of 1.5 months, a DCR of 84%, and tumor shrinkage in 79% of patients [26]. Among 53 efficacy-evaluable patients receiving glecirasib the ORR was 50.9%; within the 32 patients with PDAC in that cohort the ORR was 46.9% and the DCR 93.8% [24]. Among 22 patients with KRAS G12C-mutant PDAC, garsorasib achieved an ORR of 45.5%, with grade ≥ 3 treatment-related adverse events in 25% [27]. Separately, in the retrospectively assembled KRAS G12C PDAC cohort embedded in Dilly et al., 18 of 22 patients (81.8%) receiving adagrasib or sotorasib achieved a partial response or stable disease [28]; because that cohort was not enrolled prospectively it is reported and appraised separately from the four trials throughout.

#### 4.5.2. Survival Outcomes 

For patients with advanced PDAC treated with sotorasib, the median PFS was 4.0 months, the median duration of response was 5.7 months, and the median OS was 6.9 months [26]. For PDAC patients treated with glecirasib, the median PFS was 5.5 months and the median OS was 10.8 months [24]. These outcomes are set out in Table 7. Time-to-event outcomes were not pooled, because the two trials report them on different follow-up schedules and no individual participant data are available.

#### 4.5.3. KRAS G12D Treatment Response

Among 21 PDAC patients receiving setidegrasib (ASP3082) as a second- or third-line treatment, the ORR was 24%, while the ORR across all 31 PDAC patients was 19% [25]. For PDAC patients receiving setidegrasib, the median PFS was 3.0 months and the median OS was 10.3 months (Table 8) [25]. Being the only KRAS G12D trial identified, these results could not be pooled or compared with a second trial of the same allele.

### 4.6. Experimental Results

#### 4.6.1. KRAS G12C Monotherapy: In Vitro Activity 

Throughout this subsection, and in Table 9 and Table 10, the term activity is used in preference to efficacy, because the underlying measurements are of cell viability, proliferation, apoptosis, colony formation or signaling in culture, and these do not measure clinical effect. Sotorasib had little activity in the KRAS G12C-negative PANC-1 line, where the reported IC50 of 20 micromolar lies well above achievable clinical exposure and no cytotoxicity was observed across the clinically relevant range, whereas it was active in the KRAS G12C-mutant MIA PaCa-2 line, with IC50 values spanning 0.04 to 2.5 micromolar across studies (Table 9) [33,41,43]. Consistent with KRAS G12C selectivity, sotorasib reduced proliferation and biomass most strongly in MIA PaCa-2 cells, had minimal effect on KRAS Q61H (T3M4) cells, and produced only modest reductions in wild-type and KRAS G12V lines, with ASPC-1 and COLO357 cells showing intermediate responses (Table 10) [36]; these comparisons were made at 10 micromolar, a concentration discussed under pharmacological achievability in the Discussion. Acquired resistance was pronounced: adagrasib (MRTX849)-resistant MIA PaCa-2R cells showed a marked increase in IC50 relative to parental cells, and colony formation was unaffected by treatment (Table 9) [44]. Of these observations, the resistance findings are the ones reproduced beyond monolayer culture: in patient-derived organoids, single-agent MRTX849 or SRC inhibition did not affect HCC4300-PDX-ORG growth but inhibited HCC4285-PDX-ORG, indicating that heterogeneous single-agent sensitivity is a property of patient-derived material and not an artifact of established lines [44].

#### 4.6.2. Combination Therapy: In Vitro and In Vivo Activity

In MIA PaCa-2 cells, sotorasib combined with camptothecin gave a combination index (CI) below 1 at all tested concentrations (CI = 0.615). Compared with either monotherapy, the combination suppressed colony formation, reduced wound closure: 45% at 48 h versus 69% with sotorasib and 73% with camptothecin, indicating inhibition of migration—and increased apoptosis [43]. This corrects a statement in the previous version of this article, which described wound closure as increased; the source reports the opposite, and the direction of effect is consistent with an antimigratory rather than a promigratory action. This evidence is confined to two-dimensional monolayer culture. Dasatinib plus MRTX849 achieved a higher synergy score than palbociclib plus MRTX849, producing greater growth inhibition and reduced colony formation across 11 mutant cell lines than either agent alone, and sotorasib plus palbociclib reduced proliferation in MIA PaCa-2 cells [41]; the palbociclib combination was subsequently confirmed in patient-derived organoids and in vivo [41]. MRTX849 combined with SRC inhibitors significantly reduced proliferation in HCC4300-PDX-ORG, a patient-derived organoid [44]. The three-drug combination of sotorasib, trametinib and fedratinib achieved a synergistic CI of 0.34 in culture [38] and was subsequently examined in xenografts [38]. Synergy metrics are not interchangeable and are reported here with the metric named in each case: the Chou–Talalay combination index (values below 1 denoting synergy) [38,41,43], the Bliss independence score [47], and the Loewe additivity score [39]. Because these metrics rest on different null models of additivity, numerical values are not comparable between studies, and no combination is ranked against another on the basis of its score.

#### 4.6.3. KRAS G12D Monotherapy: In Vitro Activity

MRTX1133 inhibited the viability of KRAS G12D lines at low-nanomolar concentrations, with substantially weaker activity against non-mutant cells (Table 10) [31]. Sensitivity was heterogeneous across models: ASPC1, HPAF-II, and 828 cells were sensitive, whereas 827, 1222, 3226, and 1229 cells were largely resistant, although MRTX1133 still inhibited proliferation in the 2D-resistant lines [35]. Homozygous SW1990 and ASPC1 cells were highly sensitive, whereas heterozygous PANC-1 cells were resistant and plateaued before reaching 50% viability inhibition (Table 10) [39]. Potency spanned roughly two orders of magnitude across lines, from the most sensitive lines to PANC-1 (Table 10) [46], and PANC-1 and SW1990 cells were less responsive than Panc 04.03 and HPAC cells [32]. In ASPC-1 cells, MRTX1133 was cytotoxic, whereas the conjugate MDP5 was substantially less potent (Table 10) [30]. Patient-derived organoids spanned a range from dose-dependent sensitivity to near-complete resistance [28]. The resistant PF405 line was insensitive to MRTX1133 but sensitive to the M1C antibody–drug conjugate (ADC), while the remaining lines showed variable sensitivity to MRTX1133, the M1C ADC, or both (Table 10) [40]. The pattern of allele-selective potency is reproduced in patient-derived organoids and in vivo [28,31,34,37,40], and does not depend on the cell-line evidence alone. All of these values were generated with MRTX1133, a compound for which no human pharmacokinetic data exist; the implications are set out under pharmacological achievability in the Discussion.

#### 4.6.4. KRAS G12D Combination Therapy: In Vitro and In Vivo Activity

In PANC-1 cells, sotorasib plus camptothecin gave a CI below 1 at all points (CI = 0.621); compared with monotherapy the combination suppressed colony formation, reduced wound closure to 42% at 48 h versus 60% and 74% for the single agents, and increased apoptosis [43]. Pharmacological combinations with mTOR inhibitors (everolimus) or EGFR inhibitors (gefitinib) induced a durable arrest [35]. Tipifarnib and high-dose lonafarnib showed synergy with MRTX1133 by the Loewe additivity method, tipifarnib achieving a Loewe score of 12 in 2D and 11.5 in 3D conditions and lonafarnib an overall average of 6.182; this evidence is confined to culture [39]. Chemotherapeutic synergy with MRTX1133 was observed with 5-FU and ONC212 across multiple cell lines, again in monolayer culture only [46].

Cisplatin combined with MRTX1133 demonstrated synergy (Bliss scores > 15) across AsPC-1, PANC-1, and SW1990 cells, whereas combinations with gemcitabine or paclitaxel yielded minimal or no synergy (Bliss scores < 10) [47]. In another study, MRTX1133 or sotorasib (AMG-510) combined with gemcitabine significantly decreased the AUC in KRAS G12C/G12D cells compared with WT/G12R cells, in patient-derived primary cultures [29]. Autophagy inhibitors (spautin-1, chloroquine and bafilomycin A1) increased the anticancer activity of MRTX1133 in PANC-1 and SW1990 cells, with confirmation in mouse models [32]. MRTX1133 combined with GO-203 significantly enhanced sensitivity in AsPC-1 and PANC-1 cells, and GO-203 also showed synergy with MRTX1133 in HPAF-II cells [40]. MRTX1133 combined with the M1C ADC enhanced cell killing at lower concentrations, whereas unconjugated antibodies had no apparent effect [40]. MRTX1133 combined with olaparib produced synergy (Bliss scores > 5) and significantly reduced clonogenic survival in mutant cell lines while inducing moderate growth inhibition in non-transformed cells, with confirmation in xenografts [47]. MRTX1133 combined with trametinib and fedratinib achieved a synergistic CI below 0.5 in KP-4 cells [38].

MRTX1133 plus palbociclib diminished proliferation, as did RMC-7977 plus palbociclib [41]. BET inhibitors (JQ1 and OTX-015) combined with MRTX1133 suppressed proliferation in 2D cultures and specifically in 3D PANC1K cultures [42]. The combination of MRTX1133 and MDP5 inhibited proliferation and achieved a synergistic CI below 1 in Panc-1 and AsPC-1 cells, with reduced tumor burden in vivo [30]. Six of seven patient-derived organoids were sensitive to the M1C ADC, and four of seven exhibited synergy with MRTX1133; the combination demonstrated synergistic killing in the resistant PF405 line [40]. MRTX1133 combined with palbociclib induced apoptosis and long-term cell-cycle arrest in patient-derived organoids [41]. The combinations in this paragraph are those for which confirmation in a patient-derived or in vivo system is available, and they are correspondingly the ones on which the combination-therapy conclusion of this review rests.

#### 4.6.5. KRAS G12V: In Vitro Activity

MRTX1133 retained activity against KRAS G12V cell lines and against NCI-H727 cells, although at micromolar rather than nanomolar concentrations, that is, roughly two orders of magnitude above its potency in G12D lines (Table 10) [31,46]. The KRAS::SOS1 inhibitor BI-3406 produced only modest reductions in proliferation and biomass in the G12V lines CAPAN-1 and PATU8902 (Table 10) [36], and sotorasib likewise achieved only limited biomass reduction in KRAS G12V lines (Table 10) [36]. Higher-tier evidence for this allele is confined to two studies: Hallin et al. [31], in which G12V lines were tested alongside patient-derived xenograft work directed at G12D, and Paulsohn et al. [41], in which KRAS G12V models were evaluated within a multi-model design including patient-derived organoids and immunocompetent mice. The remainder of the KRAS G12V evidence in this review derives from conventional two-dimensional cell lines, and the BI-3406 and sotorasib comparisons were made at 10 micromolar, above achievable clinical exposure. These findings are therefore largely hypothesis-generating, which is notable given that KRAS G12V is among the most common alleles in PDAC.

#### 4.6.6. Combination Therapy: In Vitro Activity

MRTX1133 combined with 5-FU yielded a CI between 0.1 and 0.8 across the Capan-2, LS513, SNUC2B, and HCT-116 cell lines, and MRTX1133 combined with ONC212 achieved a synergistic CI of 0.03–0.85 in Capan-2 cells [46]. Both observations are confined to monolayer culture and, in part, to non-PDAC lines.

#### 4.6.7. Broader RAS Activity: In Vitro Activity

Across broader RAS contexts, BI-3406 produced modest single-agent reductions in proliferation and biomass in wild-type BxPC-3 cells and larger reductions across KRAS G12D (ASPC-1, COLO357), KRAS G12C (MIA PaCa-2), and KRAS Q61H (T3M4) lines at the highest tested dose (Table 10) [36]. Sotorasib produced only limited reductions in wild-type lines (Table 10) [36], and wild-type cells were resistant to MRTX1133-induced viability loss, with BxPC-3 showing an IC50 above the tested range (Table 10) [46]. As with the G12V evidence, these comparisons rest on two-dimensional culture at supratherapeutic concentrations and establish selectivity rather than clinical activity.

### 4.7. Objective Response Rate (ORR) 

Quantitative pooling was restricted to the clinical evidence. It was further restricted to single-arm early-phase trials of KRAS G12C inhibitors reporting the same dichotomous outcome, because these are the only clinically comparable datasets available: the experimental studies were synthesized narratively and contribute to no pooled estimate, and the single KRAS G12D trial [25] has no comparator trial with which it could be pooled. Three studies contribute to each pooled outcome. This number is not a choice but the complete set of eligible datasets: for objective response and grade ≥ 3 adverse events these are the sotorasib [26], glecirasib [24] and garsorasib [27] trials, which are all the published PDAC-specific KRAS G12C trials meeting the eligibility criteria; the fourth trial [25] targets a different allele. For disease control, garsorasib did not report the outcome for the PDAC cohort, so the sotorasib and glecirasib trials were joined by the RECIST-assessed KRAS G12C PDAC cohort embedded in Dilly et al. [28]. Admitting that cohort is the second documented departure from the a priori protocol, which had restricted quantitative synthesis to early-phase trials; it was admitted so that the outcome had three contributors rather than two, since the Hartung-Knapp confidence interval is uninformative at two studies. That cohort was assembled retrospectively and is appraised under ROBINS-I at serious overall risk of bias (Supplementary File S2, Table 2). Because three studies are at the lower bound of what can be meaningfully pooled, every pooled estimate below is reported together with the individual trial proportions and their exact 95% confidence intervals, and all three are to be read as exploratory descriptions of observed activity in small, selected, early-phase populations rather than as comparative estimates of treatment efficacy. Under GRADE, the certainty of the evidence was rated very low for all three pooled outcomes (Supplementary File S3).

The objective response was reported for the PDAC cohorts of three single-arm trials: 8 of 38 patients with sotorasib (21.1%; exact 95% CI, 9.6–37.3%) [26], 15 of 32 with glecirasib (46.9%; 29.1–65.3%) [24], and 10 of 22 with garsorasib (45.5%; 24.4–67.8%) [27]. The random-effects pooled objective response rate was 36.9% (95% CI, 9.8–75.7%), with substantial between-study heterogeneity (I^2^ = 66.2%, τ^2^ = 0.30, Cochran’s Q = 5.92, df = 2, *p* = 0.052) and near-equal weights (33.1%, 35.6% and 31.4%) (Figure 4). The confidence interval spans response rates from below one in ten to above seven in ten, and on that basis the pooled value does not support any statement about the expected response rate to a KRAS G12C inhibitor in PDAC beyond the observation that responses occur. The heterogeneity is driven by the lower response to sotorasib in the PDAC-specific trial [26] relative to the glecirasib [24] and garsorasib [27] cohorts, and with three studies its source cannot be investigated by subgroup analysis or meta-regression. A leave-one-out analysis yielded pooled estimates from 31.8% to 46.3%; omitting the sotorasib cohort raised the pooled estimate to 46.3% (37.6–55.2%) and removed the heterogeneity (I^2^ = 0%), which localizes the inconsistency to that one trial rather than resolving it. A post hoc sensitivity analysis adding the D3S-001 PDAC cohort (3 of 4 patients), which was excluded from the review for insufficient sample size, produced a comparable pooled estimate of 40.3% (15.6–71.2%; I^2^ = 61.7%).

### 4.8. Disease Control Rate (DCR)

Disease control was reported for three KRAS G12C datasets: 32 of 38 patients with sotorasib (84.2%; exact 95% CI, 68.7–94.0%) [26], 30 of 32 with glecirasib (93.8%; 79.2–99.2%) [24], and 18 of 22 in the retrospectively assembled cohort receiving adagrasib or sotorasib embedded in Dilly et al. (81.8%; 59.7–94.8%) [28]. The random-effects pooled disease control rate was 85.9% (95% CI, 62.1–95.8%), with no detectable between-study heterogeneity (I^2^ = 0%, τ^2^ = 0, Q = 1.91, df = 2, *p* = 0.384); weights were 49.5%, 18.4% and 32.1% (Figure 5). The third dataset contributes almost a third of the weight and is at serious risk of bias, which is the principal reason the certainty of this estimate is rated very low despite the apparent consistency; the absence of heterogeneity across three small single-arm datasets should not be read as evidence of a stable underlying rate.

### 4.9. Leave-One-Out Sensitivity Analysis of DCR

Leave-one-out sensitivity analysis showed that sequential exclusion of individual datasets did not materially alter the pooled point estimate, which ranged from 83.3% to 88.4% [24,26,28] (Figure 6). The confidence intervals of the two-study pools are extremely wide because the Hartung-Knapp adjustment is uninformative at two studies; the point estimates, not the intervals, are the informative quantity here. Omitting the retrospectively assembled cohort [28] leaves a pooled estimate of 88.4% from the two prospective trials, so the pooled value is not an artifact of that cohort, although it remains dependent on it for the third contributor.

### 4.10. Safety (Grade ≥ 3 Treatment-Related Adverse Events)

Grade ≥ 3 treatment-related adverse events were reported in 6 of 38 patients with sotorasib (15.8%; exact 95% CI, 6.0–31.3%) [26], 15 of 54 with glecirasib (27.8%; 16.5–41.6%) [24], and 6 of 24 with garsorasib (25.0%; 9.8–46.7%) [27]. The random-effects pooled rate was 23.8% (95% CI, 11.2–43.5%), with no detectable between-study heterogeneity (I^2^ = 0%, Q = 1.80, df = 2, *p* = 0.406) and weights of 24.8%, 53.1% and 22.1% (Figure 7). One denominator requires explicit qualification. The glecirasib safety population of 54 is the broader KRAS G12C solid-tumor safety cohort of that trial and not the 32-patient PDAC efficacy subset, because a PDAC-specific grade ≥ 3 rate was not reported; it is also the most heavily weighted dataset in this pool. The estimate is therefore not strictly PDAC-specific. To quantify the consequence, the analysis was repeated with the glecirasib event count rescaled to the 32-patient PDAC subset at the observed rate, which gave a pooled estimate of 22.9% (10.1–43.8%; I^2^ = 0%); removing the glecirasib dataset entirely left 19.7% from two trials, with an uninformative interval. The pooled safety estimate is thus insensitive to the denominator used, but it remains an estimate of the toxicity of KRAS G12C inhibitors as a class in a partly non-PDAC population, and should be read as such.

## 5. Discussion

This study evaluated the allele-stratified activity of RAS inhibitors in PDAC, synthesizing the experimental and the clinical evidence separately and stratifying the experimental evidence by the level of model validation on which it rests. Activity varied by the underlying KRAS mutation, and this pattern survived the removal of cell-line-only evidence. KRAS G12C inhibitors produced disease control in early-phase trials, while KRAS G12D-targeted agents have shown encouraging activity in preclinical models and in a single first-in-human study [25,27,53]. Read together with the classification in Table 5 and Table 6, these findings support molecular stratification as a principle for the design of future trials [54], while indicating that the strength of the evidence differs sharply between alleles and between monotherapy and combination strategies.

### 5.1. Asymmetry of Clinical Maturity Between KRAS G12C and KRAS G12D

The two alleles examined clinically in this review are not at comparable stages of development, and the size of the preclinical literature is a poor guide to which is better validated. KRAS G12C-directed therapy is supported by three early-phase trials of three agents in PDAC-specific cohorts, from which pooled estimates of response, disease control and toxicity could be derived, however exploratory. KRAS G12D-directed therapy is supported by a single first-in-human trial of one agent, setidegrasib, reporting an objective response rate of 19% across 31 patients with PDAC and 24% in the second- and third-line subgroup [25]. That single trial cannot be pooled, cannot be checked against a second trial of the same allele, and cannot support any estimate of a G12D response rate. The preclinical literature reverses this ordering: 17 of the 20 experimental studies in this review include a KRAS G12D model and only 7 include a KRAS G12C model, several addressing both. It would be a serious misreading of this review to allow the weight of that preclinical literature to suggest that clinical evidence for G12D approaches that for G12C. It does not, and the gap is compounded by a compound-level discontinuity: almost all the G12D preclinical evidence was generated with MRTX1133, whereas the agent tested clinically is setidegrasib, a targeted protein degrader of a different chemotype. The preclinical and clinical G12D literatures therefore do not concern the same molecule, and the former does not validate the latter.

### 5.2. Translational Limitations of Conventional Cell-Line Models in PDAC

PDAC is distinguished among solid tumors by a dense desmoplastic stroma that can constitute the majority of the tumor mass, by an immunosuppressive and largely T-cell-excluded microenvironment, by poor vascular perfusion that limits drug delivery, and by substantial intratumoral and interpatient heterogeneity [16,17]. None of these features are present in a two-dimensional monolayer of an established cell line. A monolayer experiment can establish that an agent engages its target and that target engagement reduces the viability of cells bearing a given allele; it cannot establish that the same is true where the drug must first traverse a fibrotic stroma, where an immune compartment may either contribute to or oppose the response, and where the tumor comprises subclones of differing genotype. Established lines are further selected for growth in culture and drift genetically and phenotypically over passage, so they under-represent the diversity of patient tumors; organoid panels were developed precisely to recover that diversity [16,17]. The distinction this implies is not between good and bad experiments but between experiments that establish a mechanism and experiments that show the mechanism to be robust in a physiologically relevant tumor context. In this review, 7 studies [28,34,35,37,40,41,44] provide the second kind of evidence through multi-model validation; 7 further studies contribute in vivo evidence at a single tier, one in patient-derived xenografts [31] and six in cell-line-derived, syngeneic or orthotopic models [30,32,38,42,45,47]; and 6 provide no in vivo evidence at all [29,33,36,39,43,46], of which 4 rest on conventional monolayer culture alone. Readers seeking evidence that a strategy is likely to work in patients should weight the first group accordingly; the classification in Table 5 is provided so that this can be done directly.

### 5.3. Pharmacological Achievability of the In Vitro Concentrations

A related question is whether the concentrations at which activity was observed are attainable in patients. For sotorasib, the comparison can be made: at the licensed dose of 960 mg once daily the steady-state apparent clearance is 26.2 L/h [55], corresponding to an average steady-state total plasma concentration of approximately 1.5 micrograms per milliliter, or about 2.7 micromolar, of which approximately 11% is unbound given plasma protein binding of 89% [55]. The sotorasib IC50 values of 0.04 and 0.25 micromolar reported in MIA PaCa-2 cells [33,43] are therefore within the plausible clinical range, and the value of 2.5 micromolar [41] sits at its upper edge. By contrast, the 10-micromolar exposures used for the cross-allele comparisons [36] exceed average total steady-state exposure by about four-fold and unbound exposure by about thirty-fold, and the 20-micromolar IC50 reported in PANC-1 [43] exceeds them by about seven-fold and about seventy-fold respectively; conclusions resting on either should be treated as statements about pharmacology at supratherapeutic concentrations. The same applies to the 60.2-micromolar IC50 reported in adagrasib-resistant cells [44], which is best read as a qualitative demonstration of resistance rather than as a quantitative potency estimate; adagrasib is additionally 98% protein bound [56], so its unbound exposure is a small fraction of the total. For MRTX1133 the comparison cannot be made at all. The compound is not orally bioavailable and was administered parenterally in the source studies [57]; no human pharmacokinetic data have been published, and a first-in-human study has only recently opened. Its potency of 5 to 50 nanomolar in sensitive G12D lines is, therefore, not translatable into a statement about achievable exposure, and neither is the synergy observed when it is combined with other agents. This is not a criticism of those studies, which were designed to establish mechanism; it is a limit on what may be inferred from them clinically, and it applies to the majority of the KRAS G12D combination literature synthesized here. The same limitation applies to BI-3406, evaluated at 10 micromolar [36], which has not progressed to clinical use in PDAC. The full appraisal, value by value, is given in Supplementary File S5.

### 5.4. Publication Bias in the Preclinical Literature

Publication bias is generally more severe in preclinical than in clinical research, because there is no registration requirement, no protocol to compare results against, and little incentive to report a drug combination that failed to synergize or a cell line that failed to respond; in the one field where it has been measured systematically, the effect was large enough to overstate efficacy substantially [58]. Every mechanism that produces it is present in the literature synthesized here. Negative combination experiments are almost absent from the 20 included studies, and the few reported negatives—the absence of synergy between MRTX1133 and gemcitabine or paclitaxel [47], and the resistance of PANC-1 to sotorasib [43]—appear as incidental comparators within reports whose principal finding was positive, never as the primary result of a study. It is therefore likely that the preclinical picture presented here, and in the field generally, is more favorable than the underlying reality, and that the true proportion of RAS-inhibitor combinations showing no useful interaction is higher than the published record suggests. No quantitative method available to us can correct for this: funnel-plot and regression-based methods require pooled effect sizes, and the experimental evidence in this review was synthesized narratively because the outcomes are not commensurable. The bias is, therefore, acknowledged as a limitation on the direction of the preclinical conclusions and is one further reason for weighting multi-model-validated findings more heavily than single-tier ones.

KRAS G12C inhibitors have the most mature clinical evidence, with objective responses and disease control observed in heavily pretreated patients [11,53], although as set out above these observations come from small single-arm cohorts. Individual KRAS alleles differ in intrinsic GTPase activity, downstream signaling and interaction with regulatory proteins, producing distinct therapeutic vulnerabilities [59]. Although KRAS G12C accounts for only a small proportion of PDAC cases, its clinical development has established proof of concept for direct KRAS inhibition. The much higher prevalence of KRAS G12D and G12V correspondingly highlights the need for allele-specific inhibitors capable of overcoming adaptive resistance [60]. Combination strategies with chemotherapy, immunotherapy, SHP2 inhibitors, EGFR inhibitors or MEK pathway inhibition enhanced activity and delayed resistance relative to monotherapy in preclinical models [61]; as the stratified reanalysis shows, that conclusion is supported by patient-derived or in vivo evidence for 11 of the 17 studies reporting a combination, while the remaining 6 have no confirmation in any higher-order system and 4 of those rest on monolayer culture alone; no combination has yet been evaluated clinically in PDAC.

This study extends the current evidence in two respects. It synthesizes therapeutic activity by specific KRAS allelic subtype rather than treating RAS inhibitors as a single class, and it grades the experimental evidence by the level of model validation achieved, so that the conclusions of the review can be read separately according to the strength of the systems that support them. Previous reviews have summarized individual RAS inhibitors or focused on clinical activity without distinguishing mutation-specific responses, and none to our knowledge has re-examined its own conclusions after separating cell-line evidence from evidence validated in patient-derived or in vivo models. These findings contribute to the evidence supporting precision medicine approaches in PDAC and identify where further clinical development is most needed.

The therapeutic landscape in RAS-mutant PDAC changed materially during the preparation of this review. Two trials of daraxonrasib (RMC-6236), an orally bioavailable RAS(ON) multi-selective inhibitor, reported outcomes in previously treated metastatic disease. In the phase 1/2 RMC-6236-001 study, the objective response rate among the 26 patients with RAS G12 mutations treated at 300 mg in the second line was 35%, with a median duration of response of 8.2 months, a median progression-free survival of 8.5 months and a median overall survival of 13.1 months [51]. In the randomized phase 3 RASolute 302 trial, daraxonrasib was compared with investigator’s choice of chemotherapy; among patients with RAS G12-mutant disease, median overall survival was 13.2 months versus 6.6 months (hazard ratio 0.40) and median progression-free survival 7.3 versus 3.5 months (hazard ratio 0.45), with objective response rates of 33.2% and 11.8% respectively. Grade ≥ 3 treatment-related adverse events occurred in 43.6% of patients receiving daraxonrasib and 57.5% of those receiving chemotherapy, and discontinuation for toxicity in 1.2% and 11.2% [52]. Both trials were identified by the updated search and were formally excluded at the eligibility stage, with the reason recorded in Figure 1: daraxonrasib acts across RAS G12 variants rather than on a single allele, so it cannot contribute to an allele-stratified comparison, and pooling a randomized comparison as a single-arm proportion would discard its concurrent control arm. They are discussed here rather than passed over because they constitute the first randomized phase 3 evidence of an overall-survival benefit from RAS inhibition in PDAC and they reframe the allele-selective findings reported above. Allele-selective and multi-selective RAS inhibition are now parallel strategies supported by markedly different levels of evidence: the allele-specific agents retain a mechanistic rationale for sparing wild-type RAS, whereas the multi-selective approach currently has by far the stronger clinical data, a randomized survival benefit, against which the exploratory single-arm estimates reported in this review should be read. Whether allele-selective inhibitors can achieve a comparable survival benefit, and whether the two approaches are best sequenced or combined, are questions for the next generation of trials.

### 5.5. Study Strengths and Limitations

This review has several methodological strengths. Eligibility was defined a priori using the PICOS framework; screening and data extraction were performed independently and in duplicate with third-reviewer adjudication; and design-specific appraisal tools were applied to the experimental and the clinical literature separately. The experimental and clinical evidence were synthesized separately rather than under a single efficacy framework, so that no preclinical observation contributes to a clinical estimate. Beyond conventional risk-of-bias appraisal, each experimental study was independently classified by the level of model validation it achieved, and the principal conclusions of the review were then re-derived under that stratification, which allows readers to separate hypothesis-generating findings from findings with translational support. The concentrations at which in vitro activity was observed were appraised against achievable clinical exposures. Every pooled estimate is reported with the individual trial proportions and exact confidence intervals that produce it, certainty was rated using GRADE, and the study-level dataset and analytic code reproducing every estimate and figure are provided in Supplementary File S4.

Several limitations qualify these findings. Most clinical evidence originated from early-phase, single-arm trials with small, selected PDAC cohorts, which precludes comparative effectiveness assessment; quantitative pooling rested on three datasets per outcome, so between-study heterogeneity statistics are unstable, subgroup analysis and meta-regression were not estimable, and the sources of the residual inconsistency in objective response could be explored only by leave-one-out analysis. Publication bias in the clinical literature could not be assessed, because with three studies per outcome no funnel-plot or asymmetry-based method is interpretable; it is, therefore, unexamined rather than excluded. The certainty of the evidence for all three pooled outcomes is very low, and the pooled estimates should be read as exploratory rather than as effect estimates suitable to guide practice. One pooled outcome depends on a retrospectively assembled cohort at serious risk of bias, and the pooled safety estimate rests partly on a non-PDAC-specific denominator, although a sensitivity analysis showed the estimate to be insensitive to that choice. The experimental evidence carries its own limitations, which the stratified reanalysis makes explicit rather than resolves: the included models are heterogeneous in kind and in translational validity, 6 of 20 studies contributed no in vivo evidence at all, several conclusions—particularly regarding combination therapy—rest partly or wholly on conventional two-dimensional culture, a substantial number of in vitro observations were made at concentrations above achievable clinical exposure or with a compound for which no human pharmacokinetic data exist, and preclinical publication bias is likely to have rendered the picture more favorable than the underlying reality. Outcome definitions and reporting conventions differed widely across experimental studies, which is why they were synthesized narratively and not pooled. Further, many emerging KRAS inhibitors remain in early clinical investigation, so long-term survival data are scarce; the review was restricted to English-language publications; and it was not prospectively registered. Finally, the pooled clinical estimates must not be extrapolated to the preclinical findings, nor the preclinical findings to patients.

### 5.6. Study Implications

The findings support molecular stratification as a fundamental component of therapeutic decision-making in PDAC. Allele-specific RAS inhibition has the potential to improve treatment selection, optimize clinical trial design, and facilitate individualized precision oncology strategies. The activity observed with combination approaches supports the continued development of rational therapeutic combinations to overcome intrinsic and acquired resistance to RAS inhibition, with the qualification established by the stratified reanalysis: that support is strongest for the combinations confirmed in patient-derived or in vivo systems, and no combination has yet been evaluated clinically in PDAC.

### 5.7. Future Research Directions

Future research should prioritize adequately powered multicenter clinical trials evaluating emerging allele-specific RAS inhibitors, particularly targeting KRAS G12D, G12V, and G12R mutations, which account for the majority of PDAC cases. Standardized reporting of efficacy outcomes, biomarker-driven patient selection, and resistance mechanisms would improve comparability across studies. Further investigations evaluating long-term survival, quality of life, treatment sequencing, and cost-effectiveness will be important for defining the optimal clinical role of RAS-targeted therapies in PDAC.

## 6. Conclusions

The evidence synthesized here is consistent with allele-dependent activity of RAS-targeted therapies in PDAC, and this conclusion survives the separation of evidence derived exclusively from conventional cell lines from evidence validated in patient-derived organoids, patient-derived xenografts, genetically engineered, syngeneic and orthotopic models: the allele-selective pattern of single-agent activity is reproduced in higher-order systems for both KRAS G12C and KRAS G12D, as is the heterogeneity of response within an allele. The conclusion that combination strategies enhance activity and delay resistance is more qualified after stratification: it is supported by patient-derived or in vivo evidence for 11 of the 17 studies reporting a combination, while for the remaining 6 there is no confirmation in any higher-order system and 4 of those rest on conventional monolayer culture alone, so it should be regarded as established in preclinical models of adequate validity for specific combinations rather than as a general property of the class. No combination has been tested clinically in PDAC. Clinically, KRAS G12C inhibitors showed disease control with an acceptable safety profile in exploratory pooled analyses of small single-arm trials, at very low certainty; KRAS G12D-targeted therapy has reached first-in-human testing with encouraging early activity but remains at a substantially earlier stage of clinical validation, and its large preclinical literature was generated predominantly with a compound that is not the agent now in trials. These findings support biomarker-guided treatment selection and allele-specific therapeutic strategies, while indicating that the principal need is for prospective trials in the alleles that dominate PDAC, KRAS G12D, G12V and G12R, and for clinical evaluation of the combinations whose preclinical support is strongest. Finally, the emergence during this review of randomized phase 3 evidence for multi-selective RAS(ON) inhibition indicates that allele-selective and multi-selective strategies will need to be compared directly rather than assessed in isolation.

## Figures and Tables

**Figure 1:**
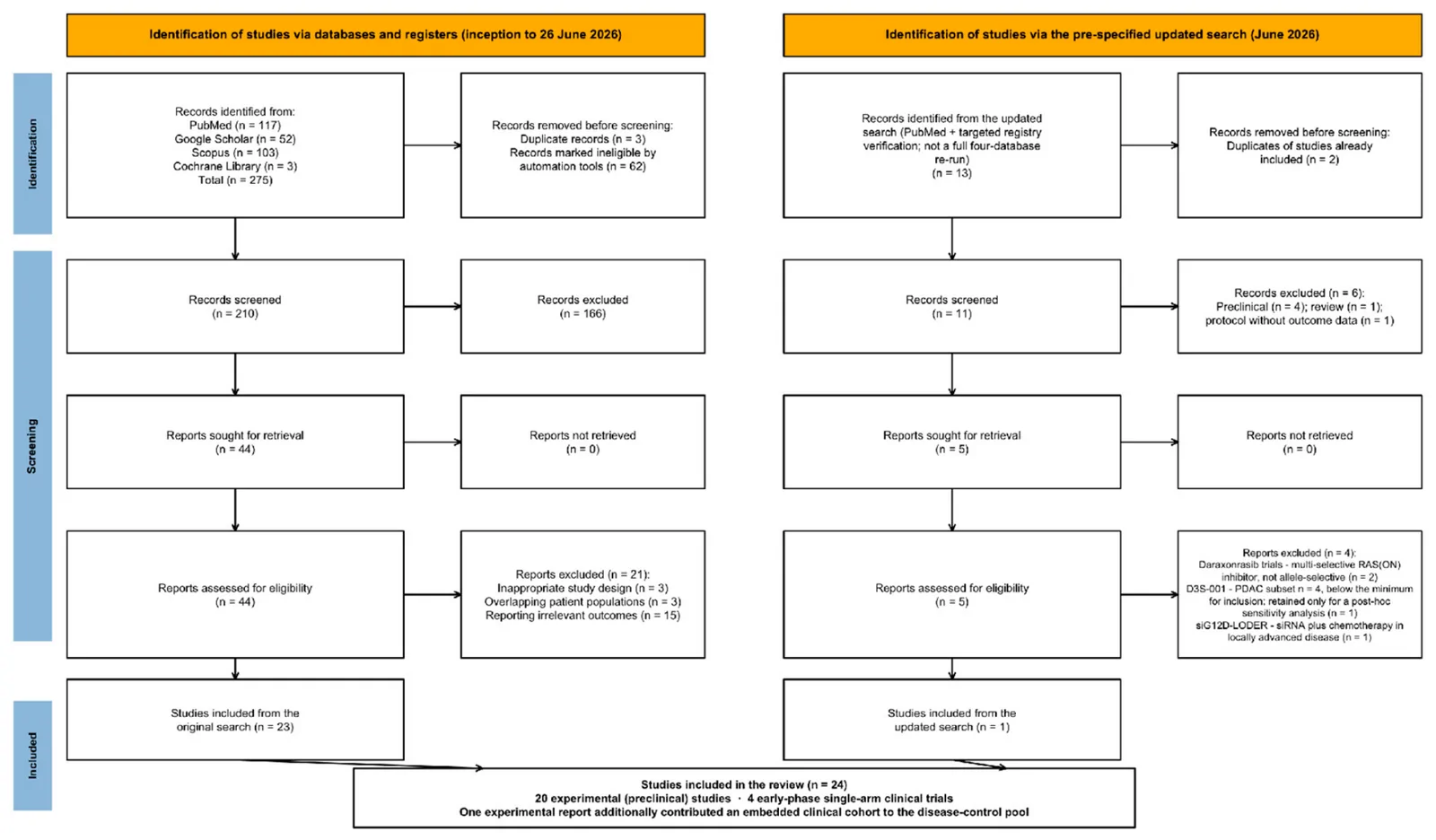
PRISMA 2020 flow diagram showing the two identification streams—the original four-database search and the pre-specified updated search—and the reports excluded at eligibility with reasons [18].

**Figure 2:**
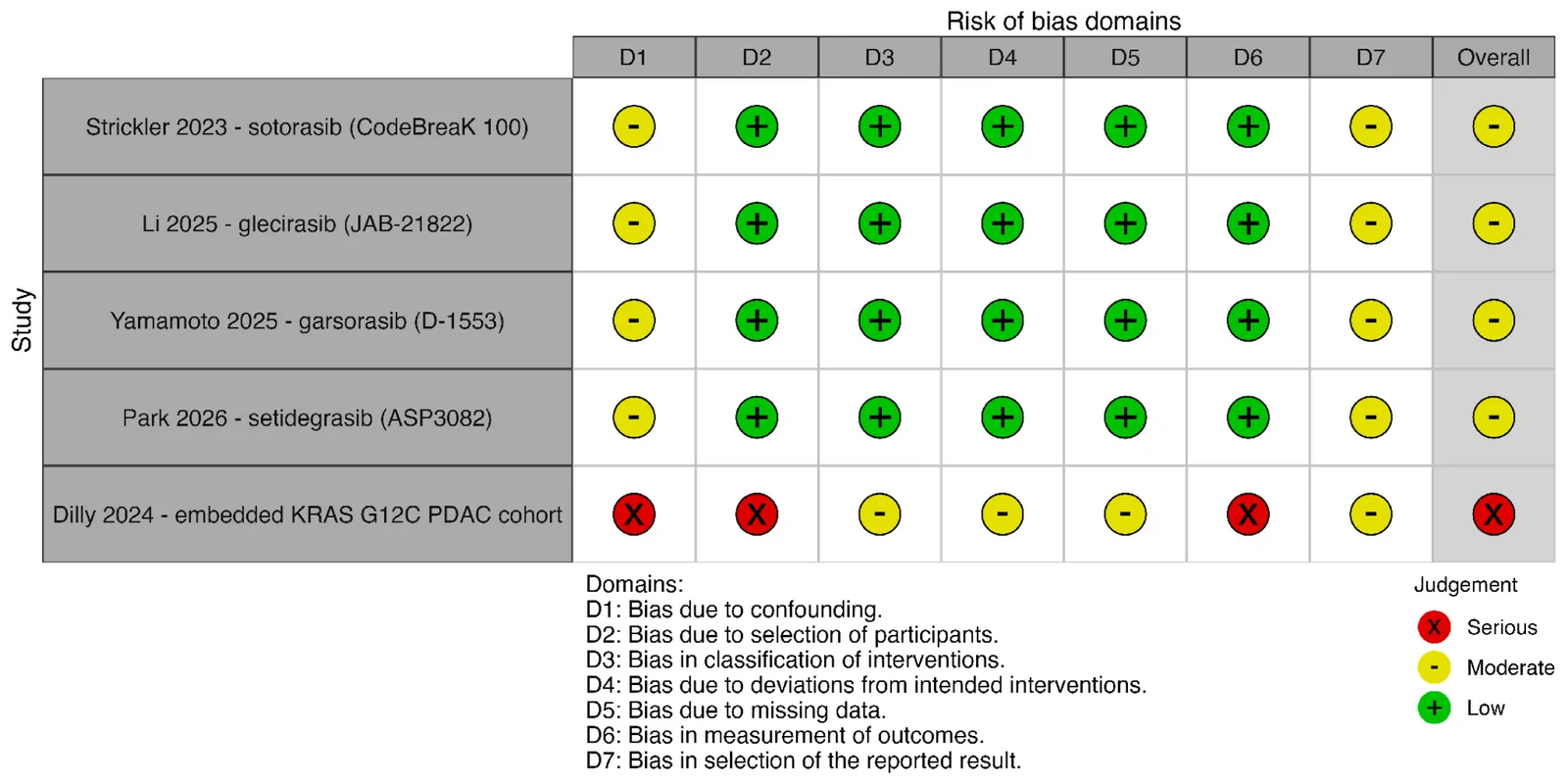
Traffic light plot of the ROBINS-I risk-of-bias assessment for the four included early-phase clinical trials and for the retrospectively assembled clinical cohort embedded in Dilly et al. [24,25,26,27,28].

**Figure 3:**
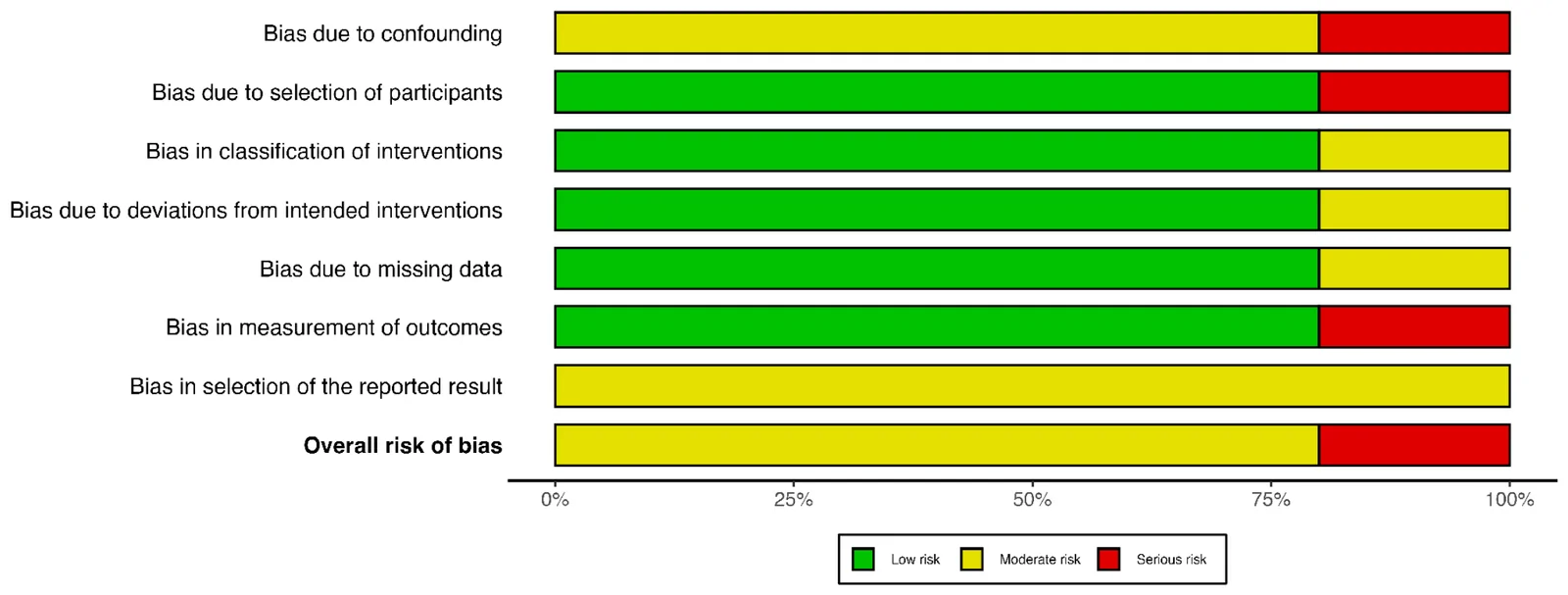
Summary plot of the ROBINS-I risk-of-bias assessment [24,25,26,27,28].

**Figure 4:**
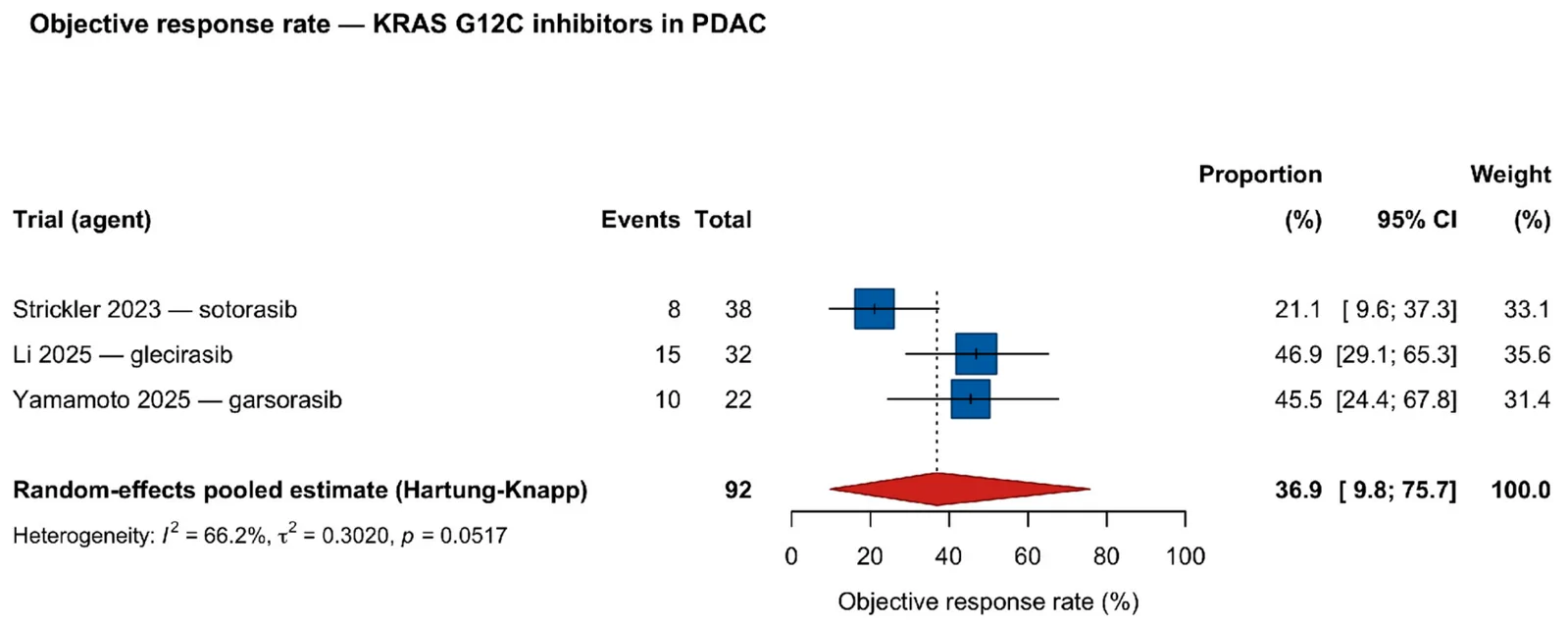
Forest plot of the objective response rate among patients with PDAC receiving KRAS G12C inhibitors, showing individual trial proportions with exact 95% confidence intervals and weights alongside the random-effects pooled estimate [24,26,27]. Blue squares are the individual trial proportions, drawn in proportion to the study weight; horizontal lines are exact 95% confidence intervals; the red diamond is the random-effects pooled estimate with its 95% confidence interval.

**Figure 5:**
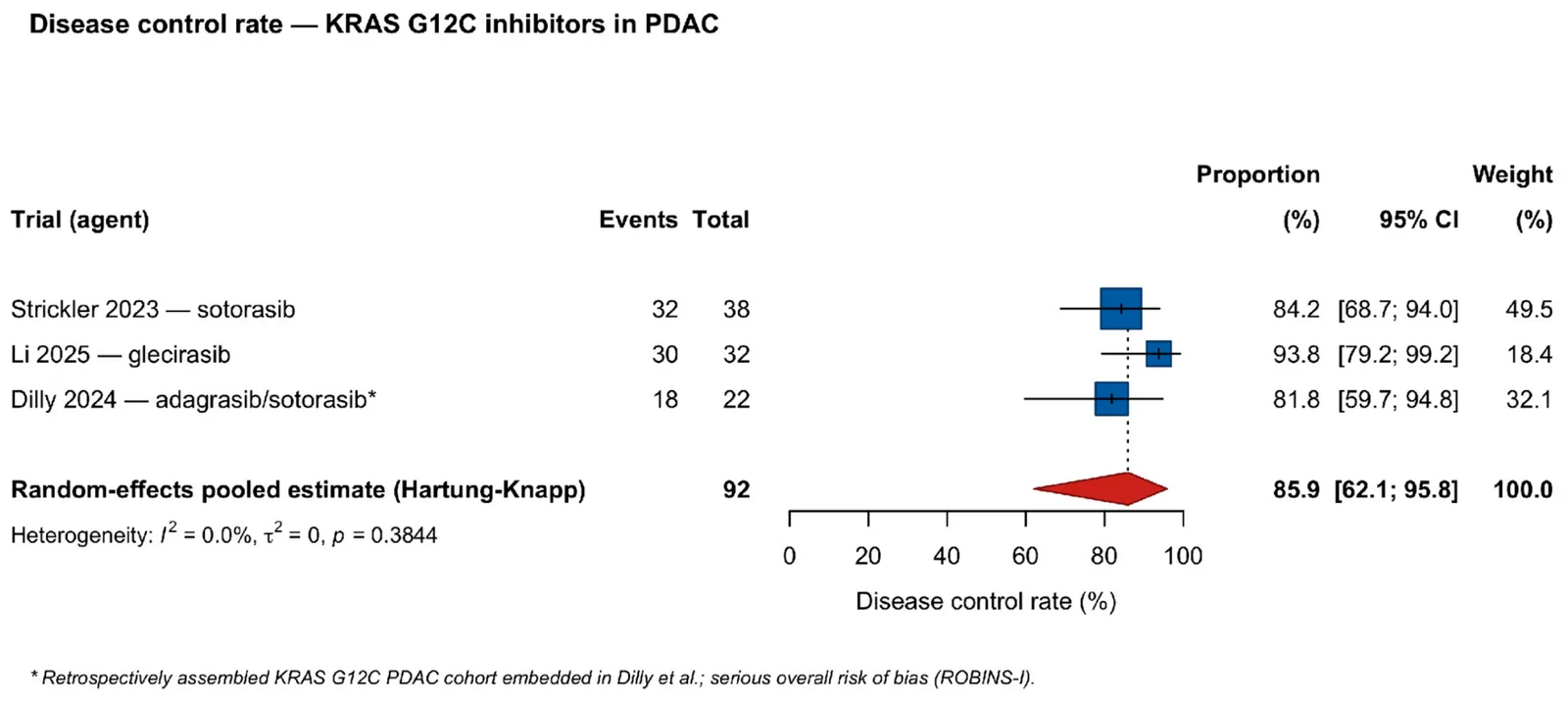
Forest plot of the disease control rate among patients with PDAC receiving KRAS G12C inhibitors, showing individual proportions with exact 95% confidence intervals and weights alongside the random-effects pooled estimate. The Dilly cohort is a retrospectively assembled dataset at serious overall risk of bias [24,26,28]. Blue squares are the individual proportions, drawn in proportion to the study weight; horizontal lines are exact 95% confidence intervals; the red diamond is the random-effects pooled estimate with its 95% confidence interval.

**Figure 6:**
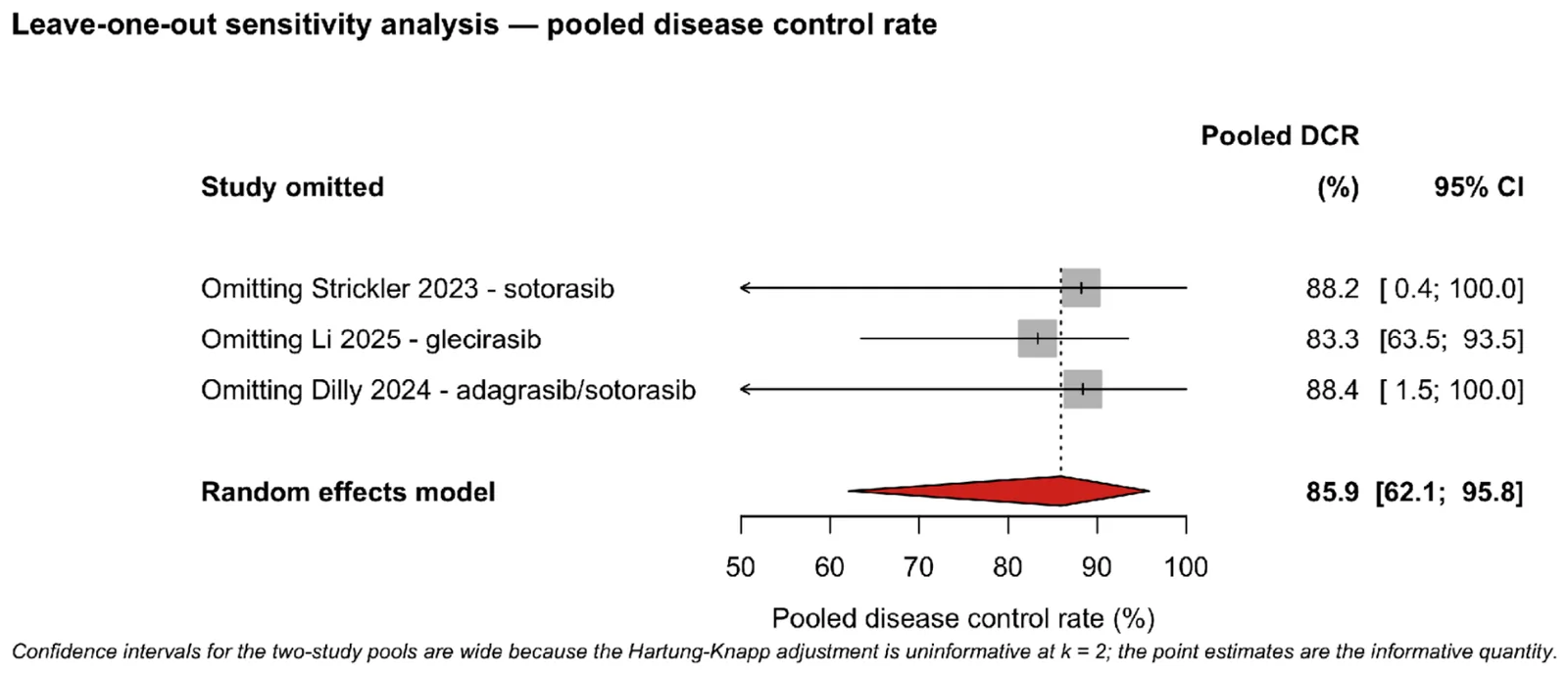
Leave-one-out sensitivity analysis for the pooled disease control rate [24,26,28]. Grey squares are the pooled estimates recomputed with the named dataset omitted; the red diamond is the pooled estimate obtained from all three datasets.

**Figure 7:**
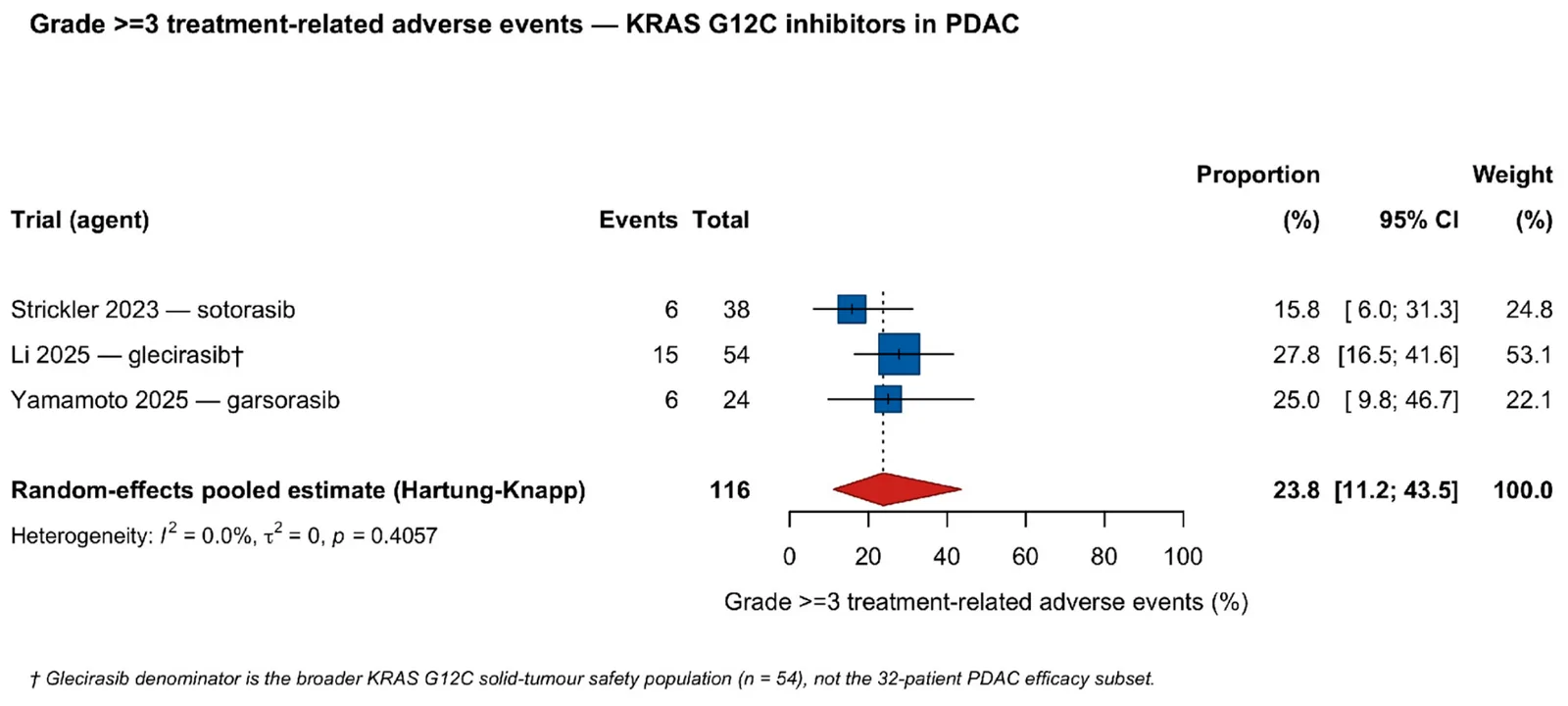
Forest plot of the incidence of grade ≥ 3 treatment-related adverse events among patients receiving KRAS G12C inhibitors, showing individual proportions with exact 95% confidence intervals and weights alongside the random-effects pooled estimate. The glecirasib denominator is the broader KRAS G12C solid-tumor safety population [24,26,27]. Blue squares are the individual proportions, drawn in proportion to the study weight; horizontal lines are exact 95% confidence intervals; the red diamond is the random-effects pooled estimate with its 95% confidence interval.

**Table 1:** JBI for quasi-experiment item-level judgment.

Domain	Yes	No	Unclear	Not Applicable (NA)
Q1. Is it clear in the study what the “cause” is and what the “effect” is (i.e., there is no confusion about which variable comes first)?	Intervention clearly preceded outcome assessment.	Temporal relationship absent or inappropriate.	Temporal sequence inadequately described.	Not applicable.
Q2. Were the experimental models similar before the intervention?	Cell lines, organoids, or animal models were comparable at baseline.	Important baseline differences existed between groups.	Baseline comparability inadequately described.	Not applicable.
Q3. Were appropriate control groups included?	Appropriate untreated, vehicle, or comparator controls were included.	No appropriate control group was used.	The control group was inadequately described.	Not applicable.
Q4. Were there multiple measurements of the outcome before and after the intervention?	Repeated or complementary outcome assessments were performed where appropriate.	Outcomes measured only once without validation.	Measurement schedule inadequately described.	Applicable when repeated measurements were not expected.
Q5. Was follow-up complete and, if not, were differences adequately described and analyzed?	Experimental follow-up was complete or adequately accounted for.	Significant missing observations not addressed.	Completeness of follow-up unclear.	Purely in vitro studies without longitudinal follow-up.
Q6. Were outcomes measured in the same way for all experimental groups?	Identical laboratory methods were used across all groups.	Different outcome assessment methods were applied.	Outcome assessment methods inadequately described.	Not applicable.
Q7. Were outcomes measured in a reliable way?	Standardized and validated laboratory techniques were used.	Outcome measurement methods were unreliable.	Reliability of measurements unclear.	Not applicable.
Q8. Was appropriate statistical analysis used?	Appropriate statistical analyses were performed and reported.	Inappropriate or absent statistical analysis.	Statistical methods inadequately described.	Not applicable.
Q9. Was the intervention clearly described and consistently implemented?	Drug(s), dose(s), treatment duration, and procedures clearly reported.	Intervention inconsistently applied or poorly described.	Important intervention details missing.	Not applicable.
Overall appraisal	Include	Exclude	Seek Further Information	—

**Table 2:** ROBINS-I tool item-level judgement for clinical studies.

Domain	Low Risk of Bias	Moderate Risk of Bias	Serious Risk of Bias	Critical Risk of Bias	No Information (NI)
D1: Bias due to Confounding	Important prognostic factors (e.g., ECOG, prior therapy, disease stage, co-mutations) appropriately considered or adjusted.	Some residual confounding likely but unlikely to substantially alter efficacy estimates.	Major prognostic factors not considered or inadequately addressed.	Confounding is severe and results are unlikely to represent the intervention effect.	Insufficient information regarding important confounders.
D2: Bias in Selection of Participants	Eligibility criteria clearly defined and participants appropriately enrolled.	Minor concerns regarding participant selection or enrollment procedures.	Selection methods likely introduced systematic differences between participants.	Selection bias severely compromises study validity.	Participant selection procedures inadequately described.
D3: Bias in Classification of Interventions	KRAS/RAS mutation status and RAS inhibitor exposure accurately classified using validated molecular testing.	Minor concerns regarding intervention or molecular classification.	Misclassification of intervention or mutation status likely affected findings.	Intervention or mutation classification fundamentally unreliable.	Insufficient information regarding intervention or mutation classification.
D4: Bias due to Deviations from Intended Interventions	Intervention delivered as planned with minimal protocol deviations.	Minor protocol deviations unlikely to influence outcomes substantially.	Important deviations likely influenced efficacy or safety outcomes.	Major deviations severely compromise interpretation of intervention effects.	Information regarding protocol deviations not reported.
D5: Bias due to Missing Outcome Data	Missing outcome data minimal or appropriately addressed.	Moderate missing data with limited impact on study conclusions.	Missing outcome data likely introduced important bias.	Extensive missing outcome data severely compromises validity.	Missing outcome data not adequately reported.
D6: Bias in Measurement of Outcomes	Outcomes assessed using standardized RECIST criteria and validated safety assessments with minimal measurement bias.	Minor concerns regarding outcome assessment procedures.	Outcome assessment likely influenced by knowledge of intervention.	Outcome measurements unreliable or systematically biased.	Outcome assessment procedures insufficiently described.
D7: Bias in Selection of the Reported Result	Outcomes and analyses reported according to the prespecified protocol or trial registration.	Some concerns regarding selective reporting.	Evidence of selective reporting or multiple unplanned analyses.	Reporting bias seriously undermines study credibility.	Reporting methods inadequately described.
Overall ROBINS-I Judgment	All domains judged as low risk.	Low risk in most domains with at least one moderate concern.	At least one domain judged as serious risk.	At least one domain judged as critical risk.	Substantial lack of information across multiple domains.

**Table 3:** Modified JBI item-level appraisal of internal methodological quality for the 20 experimental studies. This appraisal concerns internal validity only; biological model validity and translational relevance are assessed separately [28,29,30,31,32,33,34,35,36,37,38,39,40,41,42,43,44,45,46,47].

Study ID	Q1	Q2	Q3	Q4	Q5	Q6	Q7	Q8	Q9	Overall
Abuelafia et al. (2025) [29]	Yes	Yes	Yes	Yes	NA	Yes	Yes	Yes	Yes	Include
Dilly et al. (2024) [28]	Yes	Unclear	Yes	Yes	Yes	Yes	Yes	Yes	Yes	Include
Ghanwatkar et al. (2025) [30]	Yes	Yes	Yes	Yes	Yes	Yes	Yes	Yes	Yes	Include
Hallin et al. (2022) [31]	Yes	Yes	Yes	Yes	Yes	Yes	Yes	Yes	Yes	Include
Han et al. (2024) [32]	Yes	Yes	Yes	Yes	Yes	Yes	Yes	Yes	Yes	Include
Jesenko et al. (2025) [33]	Yes	Yes	Yes	Unclear	NA	Yes	Yes	Yes	Yes	Include
Jiang et al. (2025) [34]	Yes	Yes	Yes	Yes	Yes	Yes	Yes	Yes	Yes	Include
Kumarasamy et al. (2024) [35]	Yes	Yes	Yes	Yes	Yes	Yes	Yes	Yes	Yes	Include
Ma et al. (2022) [36]	Yes	Unclear	Yes	Yes	NA	Yes	Yes	Yes	Yes	Include
Mahadevan et al. (2023) [37]	Yes	Yes	Yes	Yes	Yes	Yes	Yes	Yes	Yes	Include
Miyazaki et al. (2024) [38]	Yes	Yes	Yes	Yes	Yes	Yes	Yes	Yes	Yes	Include
Molnár et al. (2024) [39]	Yes	Unclear	Yes	Yes	NA	Yes	Yes	Yes	Yes	Include
Ozawa et al. (2025) [40]	Yes	Yes	Yes	Yes	Yes	Yes	Yes	Yes	Yes	Include
Paulsohn et al. (2025) [41]	Yes	Yes	Yes	Yes	Yes	Yes	Yes	Yes	Yes	Include
Principe et al. (2026) [42]	Yes	Yes	Yes	Yes	Yes	Yes	Yes	Yes	Yes	Include
Ramalingam et al. (2025) [43]	Yes	Yes	Yes	Unclear	NA	Yes	Yes	Yes	Yes	Include
Song et al. (2024) [44]	Yes	Yes	Yes	Yes	Yes	Yes	Yes	Yes	Yes	Include
Szigeti et al. (2026) [45]	Yes	Yes	Yes	Yes	Yes	Yes	Yes	Yes	Yes	Include
Tajiknia et al. (2024) [46]	Yes	Unclear	Yes	Yes	NA	Yes	Yes	Yes	Yes	Include
Xu et al. (2026) [47]	Yes	Yes	Yes	Yes	Yes	Yes	Yes	Yes	Yes	Include

**Table 4:** Characteristics of the four included early-phase clinical trials [24,25,26,27].

Study ID	Study Design	Country	Sample Characteristics	Tumor Molecular Characteristics	Intervention	Target Allele	Outcome Measures	Findings
(Li et al., 2025) [24]	Non-randomized	China	Advanced KRAS G12C-mutant PDAC patients	KRAS G12C	Glecirasib	G12C	ORR, DCR, PFS, OS, Safety	Glecirasib produced an ORR of 46.9% and a DCR of 93.8% in the 32 patients with KRAS G12C-mutant PDAC; grade ≥ 3 TRAEs in 27.8% of the broader KRAS G12C solid-tumor safety population.
(Strickler et al., 2023) [26]	Non-randomized	United States	Previously treated metastatic KRAS G12C-mutant PDAC patients	KRAS G12C	Sotorasib	G12C	ORR, DCR, PFS, OS, DoR, Safety	Sotorasib produced an ORR of 21.1% and a DCR of 84.2% in 38 patients with KRAS G12C-mutant PDAC; grade ≥ 3 TRAEs in 15.8%.
(Park et al., 2026) [25]	Non-randomized	United States	Advanced KRAS G12D-mutant PDAC patients	KRAS G12D	Setidegrasib	G12D	ORR, PFS, OS, Safety	Setidegrasib produced an ORR of 19% across 31 patients with KRAS G12D-mutant PDAC and 24% in the second- and third-line subgroup; early clinical activity in a first-in-human setting.
(Yamamoto et al., 2025) [27]	Non-randomized	Multinational (Japan, China, Australia, Republic of Korea)	Advanced KRAS G12C-mutant PDAC patients	KRAS G12C	Garsorasib (D-1553)	G12C	ORR, Safety	Garsorasib achieved an ORR of 45.5% in 22 patients with G12C-mutant PDAC; grade ≥ 3 TRAEs in 25%.

**Table 5:** Characteristics of the 20 included experimental studies, with the highest level of model validation achieved and whether the principal finding was reproduced across independent model systems [28,29,30,31,32,33,34,35,36,37,38,39,40,41,42,43,44,45,46,47].

Study ID	Study Design	Country	Sample Characteristics	Tumor Molecular Characteristics	Intervention	Target Allele	Outcome Measures	Findings	Highest Level of Model Validation Achieved	Principal Finding Reproduced Across Independent Model Systems
(Abuelafia et al., 2025) [29]	Experimental	Argentina	MiaPaCa-2, Panc-1, BxPC-3, PDAC001T, PDAC022T, PDAC024T, PDAC054T, PDAC064T	KRAS G12C, G12D, G12R, WT	Gemcitabine, MRTX1133, AMG-510	G12D, G12C	Cell viability, Senescence, ERK/AKT signaling	MRTX1133 and AMG-510 enhanced gemcitabine sensitivity in G12D/G12C PDAC cells.	Patient-derived primary cultures in vitro (cell lines plus PDAC001T-064T primary cultures); no in vivo component	Partly
(Dilly et al., 2024) [28]	Experimental	United States	PDAC patients, PDAC cell lines, organoids, KPC mice, PDX models	KRAS G12C, G12D	Adagrasib, Sotorasib, MRTX1133, Chemotherapy	G12C, G12D	Response, Resistance, Tumor regression	G12C PDAC initially responded to adagrasib/sotorasib but acquired resistance emerged.	Multi-model validation (cell lines, organoids, PDX, KPC GEMM, patient cohort)	Yes
(Ghanwatkar et al., 2025) [30]	Experimental	United States	PANC-1, AsPC-1, KPC/Y, UN-KPC-961	KRAS G12D	MRTX1133, MDP5 nanoparticles	G12D	Cell viability, Apoptosis, Tumor burden	MRTX1133 plus MDP5 improved G12D PDAC inhibition in PANC-1/AsPC-1 cells.	Cell lines plus syngeneic/orthotopic in vivo (KPC/Y, UN-KPC-961)	Yes
(Hallin et al., 2022) [31]	Experimental	United States	KRAS G12D cell lines, Xenografts, PDX models	KRAS G12D	MRTX1133	G12D	ERK inhibition, Tumor regression	MRTX1133 selectively inhibited G12D PDAC models with marked tumor regression.	Patient-derived xenograft (2D/3D cell lines, CDX and PDX)	Yes
(Han et al., 2024) [32]	Experimental	China	PANC-1, SW1990, Panc04.03, HPAC, Mouse models	KRAS G12D	MRTX1133, Chloroquine, Bafilomycin A1, Spautin-1	G12D	Autophagy, Apoptosis, Tumor growth	Autophagy blockade increased MRTX1133 activity in G12D PANC-1/SW1990 cells.	Cell lines plus cell-line-derived xenograft	Yes
(Jesenko et al., 2025) [33]	Experimental	Slovenia	MIA PaCa-2, PANC-1	KRAS G12C, G12D	Sotorasib, Bleomycin, Cisplatin	G12C	Cell viability, Cytotoxicity	Sotorasib enhanced cytotoxicity mainly in G12C MIA PaCa-2 cells.	Conventional 2D cell lines only	No
(Jiang et al., 2025) [34]	Experimental	China	Patient-derived organoids, Patient-derived xenografts, KC mice	KRAS G12D	MRTX1133, Pentagalloylglucose, F-127 nanocarrier	G12D	Tumor regression, Metabolism	MRTX1133 plus PPP inhibition improved response in G12D PDO/PDX models.	Multi-model validation (patient-derived organoids, PDX, KC GEMM)	Yes
(Kumarasamy et al., 2024) [35]	Experimental	United States	PDAC cell lines, Spheroids, PDXs, Syngeneic mice	KRAS G12D	MRTX1133, ITGB1 inhibition	G12D	Proliferation, Immune infiltration	ITGB1 inhibition enhanced MRTX1133 sensitivity in G12D PDAC models.	Multi-model validation (cell lines, spheroids, PDX, syngeneic in vivo)	Yes
(Ma et al., 2022) [36]	Experimental	Germany	BxPC-3, AsPC-1, MIA PaCa-2, Capan-1, COLO357, T3M4, PATU8902	KRAS WT, G12C, G12D, G12V, Q61H	BI-3406, Sotorasib, Trametinib, Buparlisib	Broader RAS	Cell viability, Proliferation, Apoptosis	BI-3406 combinations improved broader RAS-mutant PDAC pathway inhibition.	Conventional 2D cell lines only	No
(Mahadevan et al., 2023) [37]	Experimental	United States	Orthotopic models, PDXs, Organoids, Mouse models	KRAS G12D	MRTX1133, Immune checkpoint blockade	G12D	Tumor regression, Survival	MRTX1133 induced immune-mediated regression in G12D PDAC in vivo models.	Multi-model validation (organoids, PDX, orthotopic immunocompetent in vivo)	Yes
(Miyazaki et al., 2024) [38]	Experimental	Japan	MIA PaCa-2, SUIT-2, KP-4, PANC-1, PK-59, Xenografts	KRAS G12C, G12D	Sotorasib, MRTX1133, Trametinib, Fedratinib	G12C, G12D	Growth inhibition, Resistance	KRAS/MEK/JAK2 inhibition overcame resistance in G12C/G12D PDAC cells.	Cell lines plus cell-line-derived xenograft	Yes
(Molnár et al., 2024) [39]	Experimental	Hungary	PANC-1, AsPC-1, SW1990	KRAS G12D	MRTX1133, Farnesyl-transferase inhibitors	G12D	Cell viability, Signaling	Farnesyl-transferase inhibition enhanced MRTX1133 activity in G12D PDAC cells.	Cell lines in 2D and 3D spheroid culture; no in vivo component	Partly
(Ozawa et al., 2025) [40]	Experimental	United States	PDAC cell lines, Organoids, PDXs, Human PDAC tumors	KRAS G12D	MRTX1133, M1C ADC, GO-203	G12D	Resistance, Viability	M1C targeting restored MRTX1133 sensitivity in resistant G12D PDAC models.	Multi-model validation (cell lines, patient-derived organoids, PDX, human tumor specimens)	Yes
(Paulsohn et al., 2025) [41]	Experimental	Germany	MIA PaCa-2, 51T-2D, AsPC-1, KPC models, GöCDX models, Patient-derived organoids	KRAS G12C, G12D, G12V, WT	Sotorasib, MRTX1133, RMC-7977, Palbociclib	G12C, G12D, G12V, Pan-RAS	Cell viability, Apoptosis, Tumor growth	KRAS/CDK4 inhibition suppressed G12C, G12D, and G12V PDAC models.	Multi-model validation (cell lines, patient-derived organoids, GöCDX, KPC GEMM)	Yes
(Principe et al., 2026) [42]	Experimental	United States	PANC-1, AsPC-1, CD18, KPC-2138, KPC-3213, FC1245	KRAS G12D	MRTX1133, JQ1, OTX-015	G12D	Resistance, RNA-seq, Survival	BET inhibition restored MRTX1133 activity in resistant G12D PDAC cells.	Cell lines in 2D and 3D plus syngeneic in vivo	Yes
(Ramalingam et al., 2025) [43]	Experimental	India	MIA PaCa-2, PANC-1	KRAS G12C, G12D	Sotorasib, Camptothecin	G12C, G12D	IC50, Apoptosis, ROS	Sotorasib plus camptothecin synergized in G12C MIA PaCa-2 and G12D PANC-1.	Conventional 2D cell lines only	No
(Song et al., 2024) [44]	Experimental	United States	MIA PaCa-2, Calu1, Resistant cells, Patient-derived organoids, Mouse models	KRAS G12C	MRTX849, Dasatinib, Bosutinib, DGY-06-116	G12C	Resistance, Tumor growth	SRC inhibition overcame MRTX849 resistance in G12C MIA PaCa-2 models.	Multi-model validation (cell lines, patient-derived organoids, in vivo)	Yes
(Szigeti et al., 2026) [45]	Experimental	Hungary	ASPC1, SW1990, PANC1, Liver metastasis mouse model	KRAS G12D	MRTX1133	G12D	Proliferation, Migration, Metastasis	MRTX1133 inhibited migration and liver colonization in G12D PDAC models.	Cell lines plus in vivo liver-metastasis model	Yes
(Tajiknia et al., 2024) [46]	Experimental	United States	BxPC-3, AsPC-1, MIA PaCa-2, CRC cell lines	KRAS G12D, G12V, G13D, WT	MRTX1133, 5-FU, ONC212	G12D, Broader RAS	Cell viability, Synergy, Signaling	MRTX1133 combinations showed synergy across G12D and broader RAS-mutant cells.	Conventional 2D cell lines only	No
(Xu et al., 2026) [47]	Experimental	China	AsPC-1, PANC-1, SW1990, KPC1199, Xenografts	KRAS G12D	MRTX1133, Olaparib	G12D	DNA damage, Tumor regression, Survival	Olaparib enhanced MRTX1133 activity in G12D AsPC-1/PANC-1/SW1990 models.	Cell lines plus cell-line-derived xenograft	Yes

**Table 6:** Reanalysis of the principal experimental conclusions of this review after stratification by the highest level of model validation achieved.

Principal Conclusion of the Review	Contributing Studies (n)	Supported by Cell-Line-Only Evidence	Supported in Patient-Derived Models (PDO/PDX/Primary)	Supported in In Vivo or Immunocompetent Models	Does the Conclusion Survive When Cell-Line-Only Evidence Is Set Aside?
Single-agent KRAS G12D inhibition produces allele-selective loss of viability and tumor regression	12 [30,31,32,34,35,37,39,40,42,45,46,47]	Yes [39,46]	Yes [31,34,35,37,40]	Yes [30,31,32,34,35,37,40,42,45,47]	Yes—unchanged; regression shown in PDX and in genetically engineered and orthotopic models
Single-agent KRAS G12C inhibition produces allele-selective activity	8 [28,29,33,36,38,41,43,44]	Yes [33,36,43]	Yes [28,29,41,44]	Yes [28,38,41,44]	Yes—unchanged, but resting on fewer studies than the G12D conclusion
Sensitivity is heterogeneous within an allele (zygosity, intrinsic resistance)	7 [28,31,35,39,40,42,44]	Yes [39]	Yes [28,35,40,44]	Yes [31,35,42,44]	Yes—and strengthened, since patient-derived organoid panels are the system in which between-patient variation is observable
Acquired resistance to KRAS inhibition arises and can be reversed pharmacologically	5 [28,38,40,42,44]	Partly [38]	Yes [28,40,44]	Yes [28,38,40,42,44]	Yes—resistance and its reversal are reproduced in patient-derived organoids and in vivo
Combination strategies enhance activity and delay resistance	17 [29,30,32,33,34,35,36,37,38,39,40,41,42,43,44,46,47]	Yes, for 4 of 17 [33,36,43,46]; 2 further studies [29,39] provided no in vivo confirmation	Yes, for 7 [29,34,35,37,40,41,44]	Yes, for 11 [30,32,34,35,37,38,40,41,42,44,47]	Only in part—supported for the 11 combinations confirmed in a higher-order system; the remaining 6, of which 4 rest on monolayer culture alone, are hypothesis-generating
KRAS inhibition acts partly through the immune compartment and the tumor microenvironment	3 [35,37,41]	Not assessable in cell lines	Yes [35,37,41]	Yes [35,37,41]	Yes—this conclusion exists only at the higher tiers and has no cell-line-derived counterpart
KRAS G12D inhibition has antimigratory and antimetastatic activity	2 [43,45]	Yes [43]	No	Yes [45]	Only in part—established in an in vivo liver-metastasis model [45]; the scratch-assay evidence [43] does not of itself establish an antimetastatic effect
Activity extends to KRAS G12V, G12R and broader RAS-mutant contexts	4 [31,36,41,46]	Yes [36,46]	Partly [31,41]	Partly [31,41]	Only in part—the evidence is predominantly cell-line-derived; G12V models appear within a broader design in only two studies [31,41], and no conclusion here rests on more than a single higher-tier study

**Table 7:** Survival outcomes reported by the KRAS G12C trials [24,26].

Study	Drug (Trial)	Median Time to Response	Median PFS (Months)	PFS 95% CI	Median OS (Months)	OS 95% CI
(Strickler et al., 2023) [26]	Sotorasib (CodeBreaK 100)	1.5 months	4	2.8 to 5.6	6.9	5.0 to 9.1
(Li et al., 2025) [24]	Glecirasib (JAB-21822)	-	5.5	4.2 to 9.8	10.8	8.9 to 12.5

**Table 8:** Survival outcomes of setidegrasib (ASP3082) in KRAS G12D-mutant PDAC [25].

Study	Drug	Median PFS (Months)	PFS 95% CI	Median OS (Months)	OS 95%CI
(Park et al., 2026) [25]	Setidegrasib (ASP3082)	3	1.4 to 6.9	10.3	4.2 to 13.0

**Table 9:** In vitro activity of KRAS G12C-targeted agents in PDAC models [33,41,43,44].

Study	Drug	Human Cell Line	Metric	Value
(Ramalingam et al., 2025) [43]	Sotorasib	MIA PaCa-2	IC50	0.04 µM
(Jesenko et al., 2025) [33]	Sotorasib	MIA PaCa-2	IC50	0.25 µM
(Song et al., 2024) [44]	MRTX849 (Adagrasib)	MIA PaCa-2	IC50	0.13 µM
(Paulsohn et al., 2025) [41]	Sotorasib	MIA PaCa-2	IC50	2.5 µM
(Jesenko et al., 2025) [33]	Sotorasib	MIA PaCa-2	Viability at 0.05 µM	80%
(Song et al., 2024) [44]	MRTX849 (Adagrasib)	MIA PaCa-2R (resistant)	IC50	60.2 µM

**Table 10:** In vitro activity of RAS-targeted agents across PDAC models, by target allele. The table includes KRAS G12D-, G12C-, G12V- and Q61H-mutant and wild-type models and the comparator agent camptothecin, since several source studies report potency across alleles within a single experiment; rows are grouped by the allele of the model tested rather than by the nominal target of the agent [30,31,35,36,37,39,40,41,42,43,45,46,47].

Study	Drug	Human Cell Line	Metric	Value
(Ramalingam et al., 2025) [43]	Sotorasib	PANC-1	IC50	20 µM
(Ma et al., 2022) [36]	Sotorasib	ASPC-1	Proliferation/Biomass reduction	50%/41% at 10 µM
(Ma et al., 2022) [36]	Sotorasib	COLO357	Proliferation/Biomass reduction	37%/27% at 10 µM
(Ghanwatkar et al., 2025) [30]	MRTX1133	AsPC-1	IC50	3.22 µM
(Kumarasamy et al., 2024) [35]	MRTX1133	ASPC1	EC50	25–50 nM
(Kumarasamy et al., 2024) [35]	MRTX1133	HPAF-II	EC50	25–50 nM
(Kumarasamy et al., 2024) [35]	MRTX1133	828	EC50	25–50 nM
(Kumarasamy et al., 2024) [35]	MRTX1133	519, 827, 1222, 3226, 1229	EC50	>1 µM
(Mahadevan et al., 2023) [37]	MRTX1133	HPAC	IC50	6.076 nM
(Molnár et al., 2024) [39]	MRTX1133	ASPC1	IC50	21 nM
(Molnár et al., 2024) [39]	MRTX1133	SW1990	IC50	11 nM
(Molnár et al., 2024) [39]	MRTX1133	PANC1	IC50	>3.125 µM
(Szigeti et al., 2026) [45]	MRTX1133	ASPC1	IC50	5.6 nM
(Szigeti et al., 2026) [45]	MRTX1133	SW1990	IC50	31.3 nM
(Tajiknia, 2024) [46]	MRTX1133	HPAF-II	IC50	1.77 µM
(Tajiknia, 2024) [46]	MRTX1133	PANC-1	IC50	2.5 µM
(Hallin et al., 2022) [31]	MRTX1133	HPAC	IC50 (3D)	3.8 nM
(Hallin et al., 2022) [31]	MRTX1133	AsPC-1	IC50 (3D)	4.8 nM
(Hallin et al., 2022) [31]	MRTX1133	Panc 04.03	IC50 (3D)	7.4 nM
(Hallin et al., 2022) [31]	MRTX1133	SW1990	IC50 (3D)	4.7 nM
(Xu et al., 2026) [47]	MRTX1133	AsPC-1	IC50	~50 nM (from dose-response)
(Xu et al., 2026) [47]	MRTX1133	PANC-1	IC50	~50 nM (from dose-response)
(Xu et al., 2026) [47]	MRTX1133	SW1990	IC50	~50 nM (from dose-response)
(Principe et al., 2026) [42]	MRTX1133	PANC-1	IC50	~0.5–1 µM (parental)
(Principe et al., 2026) [42]	MRTX1133	AsPC-1	IC50	~0.5–1 µM (parental)
(Principe et al., 2026) [42]	MRTX1133	CD18	IC50	~0.5–1 µM (parental)
(Ramalingam et al., 2025) [43]	Camptothecin	MIA PaCa-2	IC50	0.5 µM
(Ramalingam et al., 2025) [43]	Camptothecin	PANC-1	IC50	0.3 µM
(Ghanwatkar et al., 2025) [30]	MDP5	AsPC-1	IC50	18.06 µM
(Paulsohn et al., 2025) [41]	MRTX1133	AsPC-1	IC50	~0.5 µM (from dose-response)
(Hallin et al., 2022) [31]	MRTX1133	Genotyped PDAC lines (pooled)	Median IC50 (3D/2D)	5.3/26 nM (non-mutant > 211 nM)
(Tajiknia, 2024) [46]	MRTX1133	LS513	IC50	39 nM
(Ozawa et al., 2025) [40]	MRTX1133	PF405	IC50	>1 µM
(Ozawa et al., 2025) [40]	M1C ADC	PF405	IC50	31 nM
(Tajiknia, 2024) [46]	MRTX1133	G12V lines (SW480, SW620, CAPAN-1, CAPAN-2, CFPAC-1)	IC50	1.2–4 µM
(Hallin et al., 2022) [31]	MRTX1133	NCI-H727	IC50 (2D/3D)	3/1 µM
(Tajiknia, 2024) [46]	MRTX1133	BxPC-3 (WT)	IC50	>10 µM
(Ma et al., 2022) [36]	Sotorasib	MIA PaCa-2	Proliferation/Biomass reduction	69%/60% at 0.05 µM
(Ma et al., 2022) [36]	Sotorasib	T3M4 (Q61H)	Proliferation/Biomass reduction	6%/0% at 10 µM
(Ma et al., 2022) [36]	Sotorasib	WT & G12V lines	Proliferation/Biomass reduction	25–38% at 10 µM
(Ma et al., 2022) [36]	BI-3406	CAPAN-1	Proliferation/Biomass reduction	11%/12% at 10 µM
(Ma et al., 2022) [36]	BI-3406	PATU8902	Proliferation/Biomass reduction	17%/21% at 10 µM
(Ma et al., 2022) [36]	BI-3406	BxPC-3 (WT)	Proliferation/Biomass reduction	15%/27% at 10 µM
(Ma et al., 2022) [36]	BI-3406	ASPC-1, COLO357 (G12D); MIA PaCa-2 (G12C); T3M4 (Q61H)	Proliferation/Biomass reduction	30–50% at highest dose

## Data Availability

All data supporting the findings of this review are available within the article and its Supplementary Materials; the underlying datasets are derived from the previously published studies cited herein. The study-level dataset used for every pooled analysis, the data extraction template and the analytic code reproducing all pooled estimates and figures are provided in Supplementary File S4; the item-level risk-of-bias matrices in Supplementary File S2, Section 2; and the model validation and pharmacological achievability matrices in Supplementary File S5.

## Supplementary Materials

The following supporting information can be downloaded at: https://www.mdpi.com/article/10.3390/cancers18193139/s1, File S1. The completed PRISMA 2020 checklist; File S2. The full electronic search strategies for both identification streams, the item-level risk-of-bias assessments under both tools, and the list of full-text reports excluded with reasons; File S3. The GRADE certainty-of-evidence table; File S4. The study-level dataset, the data extraction template and the analytic code reproducing every pooled estimate and forest plot; and File S5. The per-study model-validation matrix and the pharmacological-achievability appraisal of every in vitro concentration extracted.

## Abbreviations

The following abbreviations are used in this manuscript:

ADC	Antibody–Drug Conjugate
AEs	Adverse Events
AKT	Protein Kinase B
ARRIVE	Animal Research: Reporting of In Vivo Experiments
AUC	Area Under the Curve
CAFs	Cancer-Associated Fibroblasts
CBR	Clinical Benefit Rate
CDX	Cell-Line-Derived Xenograft
CI	Confidence Interval; combination index (in synergy analyses)
CMA	Comprehensive Meta-Analysis
CQ	Chloroquine
CSC	Cancer Stem Cell
ctDNA	Circulating Tumor DNA
DCR	Disease Control Rate
DoR	Duration of Response
EC_50_	Half-Maximal Effective Concentration
EGFR	Epidermal Growth Factor Receptor
EMT	Epithelial-Mesenchymal Transition
ERK	Extracellular Signal-Regulated Kinase
GEMMs	Genetically Engineered Mouse Models
GRADE	Grading of Recommendations, Assessment, Development and Evaluations
HR	Hazard Ratio
I^2^	Higgins Heterogeneity Statistic
IC_50_	Half-Maximal Inhibitory Concentration
ICIs	Immune Checkpoint Inhibitors
IFN	Interferon
JBI	Joanna Briggs Institute
MAPK	Mitogen-Activated Protein Kinase
MEK	Mitogen-Activated Protein Kinase Kinase
MeSH	Medical Subject Headings
mTOR	Mammalian Target of Rapamycin
NGS	Next-Generation Sequencing
NIH	National Institutes of Health
NR	Not Reported
ORR	Objective Response Rate
OS	Overall Survival
PD	Progressive Disease
PDAC	Pancreatic Ductal Adenocarcinoma
PDOs	Patient-Derived Organoids
PDX	Patient-Derived Xenograft
PFS	Progression-Free Survival
PI3K	Phosphatidylinositol 3-Kinase
PICOS	Population, Intervention, Comparator, Outcomes, Study Design
PR	Partial Response
PRISMA	Preferred Reporting Items for Systematic Reviews and Meta-Analyses
PROSPERO	International Prospective Register of Systematic Reviews
qRT-PCR	Quantitative Reverse Transcription Polymerase Chain Reaction
RAS	Rat Sarcoma
RoB	Risk of Bias
ROBINS-I	Risk Of Bias In Non-randomised Studies—of Interventions
ROS	Reactive Oxygen Species
SD	Stable Disease
SHP2	Src Homology 2 Domain-Containing Protein Tyrosine Phosphatase 2
SRC	Src Proto-Oncogene, Non-Receptor Tyrosine Kinase
SYRCLE	Systematic Review Centre for Laboratory Animal Experimentation
TRAEs	Treatment-Related Adverse Events
WT	Wild Type
Drug codes (generic name): adagrasib (MRTX849), sotorasib (AMG-510), setidegrasib (ASP3082), garsorasib (D-1553).	
