# Supplementary material for: Allele-Stratified Activity of RAS Inhibitors in Pancreatic Ductal Adenocarcinoma: A Systematic Review and Exploratory Meta-Analysis
Source: Cancers (Basel). 2026 Sep 28;18(19):3139. doi: 10.3390/cancers18193139 (PMC13664068; doi:10.3390/cancers18193139)
Supplement: Supplementary file 1 [file cancers-18-03139-s001.zip › Supplementary_File S1_PRISMA_2020_Checklist.pdf]

Supplementary File S1. PRISMA 2020 Checklist

Allele-stratified activity of RAS inhibitors in pancreatic ductal adenocarcinoma: a systematic review with model-validation-stratified synthesis of preclinical evidence and exploratory meta-analysis of early-phase trials

Manuscript ID: cancers-4500792

Page numbers refer to the revised manuscript, clean version (Revised_manuscript_clean_v7, 66 pages) as exported to PDF from Microsoft Word. Named sections, tables and figures are retained alongside the page numbers, because pagination will change at typesetting. “Suppl. S2/S3/S4/S5” refer to the corresponding supplementary files.

**Section**	**Item**	**Checklist item**	Location where item is reported (page no. of submitted PDF)
TITLE	1	Identify the report as a systematic review.	**p. 1 — Title. Reported: “… a systematic review with model-validation-stratified synthesis of preclinical evidence and exploratory meta-analysis of early-phase trials”.**
ABSTRACT	2	See the PRISMA 2020 for Abstracts checklist.	**pp. 1-3 — Simple Summary and Abstract (structured: Background, Objectives, Methods, Results, Conclusions). Databases, date limits, risk-of-bias tools, synthesis method, the number of included studies (24: 20 experimental and 4 early-phase clinical trials) and the principal pooled findings are all stated, as is the separate synthesis of the experimental and the clinical evidence.**
INTRODUCTION	3	Describe the rationale for the review in the context of existing knowledge.	**pp. 4-5 — Introduction, paragraphs 1-4.**
	4	Provide an explicit statement of the objective(s) or question(s) the review addresses.	**p. 5 — Introduction — “Objectives” subsection.**
METHODS	5	Specify the inclusion and exclusion criteria for the review and how studies were grouped for the syntheses.	**pp. 6-8; pp. 46-47 — Methodology — “Eligibility Criteria” (Inclusion Criteria, p. 7; Exclusion Criteria, p. 8), PICOS-based and applied separately to the clinical and the experimental literature. The grouping rule for synthesis — the experimental and the clinical evidence are synthesised separately, so that no preclinical observation contributes to a clinical estimate; within the clinical evidence studies are grouped by target KRAS allele, with quantitative pooling restricted to single-arm early-phase KRAS G12C trials reporting the same dichotomous outcome — is stated in “Data Analysis” and restated at the start of the ORR subsection of Results (p. 46).**
	6	Specify all databases, registers, websites, organisations, reference lists and other sources searched or consulted. Specify the date when each source was last searched or consulted.	**pp. 6-7 — Methodology — “Search Strategy for Identifying Studies”. Two identification streams are reported. The original search covered PubMed, Cochrane Library, Scopus and Google Scholar (first 200 records), plus ClinicalTrials.gov, WHO ICTRP and citation searching of eligible studies, all from inception to 26 June 2026. A pre-specified updated search using a gap-corrected drug block was run in June 2026 (PubMed with targeted registry verification, not a full four-database re-run). Both streams are shown separately in Figure 1 (p. 19) and reported in Supplementary File S2, Section 1.**
	7	Present the full search strategies for all databases, registers and websites, including any filters and limits used.	**Suppl. S2, Sections 1.1-1.6 — verbatim strings for PubMed (1.1), Cochrane Library (1.2) and Scopus (1.3); the keyword combinations and relevance limit applied in Google Scholar (1.4); the amended intervention block and the account of the second identification stream (1.5); and per-source yields with the date each source was last searched, for both streams (1.6).**
	8	Specify the methods used to decide whether a study met the inclusion criteria of the review, including how many reviewers screened each record and each report retrieved, whether they worked independently, and if applicable, details of automation tools used in the process.	**pp. 6-7; Figure 1, p. 19 — Methodology — “Study Selection”: two reviewers screened independently at title/abstract and full text; disagreements resolved by discussion, with a third reviewer as arbiter. Zotero 6.0.36 was used for**

			**deduplication, and its publication-type filters removed 62 records matching the pre-specified excluded publication types (reviews, editorials, letters, comments, conference abstracts and errata) before manual screening; all such exclusions were verified by a reviewer. This count is shown separately in the flow diagram (Figure 1). Records from both identification streams were screened under identical criteria.**
	9	Specify the methods used to collect data from reports, including how many reviewers collected data from each report, whether they worked independently, any processes for obtaining or confirming data from investigators, and if applicable, details of automation tools used in the process.	**p. 17; Suppl. S4, Section 1 — Methodology — "Data Extraction": duplicate independent extraction using a standardised form, third-reviewer arbitration, no automation tools, and no contact with study investigators. The extraction template is reproduced in Supplementary File S4, Section 1.**
	10a	List and define all outcomes for which data were sought. Specify whether all results that were compatible with each outcome domain in each study were sought, and if not, the methods used to decide which results to collect.	**pp. 7-8; p. 50 — Methodology — "Inclusion Criteria": primary outcomes ORR, DCR, PFS, OS, DoR, CBR; secondary outcomes grade ≥3 TRAEs, allele-specific antitumour activity, resistance mechanisms. Where a trial reported both a PDAC-specific and a broader solid-tumour denominator, the denominator used is stated explicitly in Results (Safety subsection, p. 50) and its consequence quantified by sensitivity analysis.**
	10b	List and define all other variables for which data were sought.	**pp. 10, 17 — Methodology — "Data Extraction": study ID, country, study design, sample characteristics, cell lines, intervention, target allele, outcome measures, findings. For the experimental studies, the model systems reported were additionally extracted to support the post-hoc classification of model validation described on p. 10 and tabulated in Supplementary File S5.**
	11	Specify the methods used to assess risk of bias in the included studies, including details of the tool(s) used, how many reviewers assessed each study and whether they worked independently, and if applicable, details of automation tools used in the process.	**pp. 8-10; Tables 1-2, pp. 12-16; Table 3, p. 22; Figures 2-3, pp. 20-21; Suppl. S2, Section 2 — Methodology — "Risk of Bias Assessment": ROBINS-I (non-randomised clinical studies, 7 domains) and a modified JBI Critical Appraisal Checklist for Quasi-Experimental Studies (experimental studies, 9 domains). Two independent assessors, third-reviewer arbitration, no automation tools. Domain anchors are given in Table 1 (JBI) and Table 2 (ROBINS-I); item-level judgements in Table 3 and Figures 2-3, with the complete matrices in Supplementary File S2, Section 2. Internal methodological quality and biological model validity are treated as distinct properties: the latter is appraised by the post-hoc model-validation classification (p. 10; Table 5; Supplementary File S5), not by the JBI checklist. The KRAS G12C clinical cohort embedded in Dilly et al. [22], which contributes to the disease-control synthesis, was additionally appraised under ROBINS-I (Supplementary File S2, Section 2.1).**
	12	Specify for each outcome the effect measure(s) (e.g. risk ratio, mean difference) used in the synthesis or presentation of results.	**pp. 17-18; Tables 7-8, pp. 37-38 — Methodology — "Data Analysis": single-arm proportions (ORR, DCR, grade ≥3 TRAEs) pooled on the logit scale and back-transformed to percentages with 95% CIs; each individual trial proportion is additionally reported with an exact (Clopper-Pearson) 95% CI. Time-to-event outcomes (PFS and OS) were not pooled and are presented as the medians and 95% CIs reported by each trial (Tables 7 and 8); duration of response is reported narratively as a median only, because no trial reported an interval for it.**
	13a	Describe the processes used to decide which studies were eligible for each synthesis (e.g. tabulating the study intervention characteristics and comparing against the planned groups for each synthesis).	**pp. 17-18; p. 46; Tables 4-6, pp. 24-36 — Methodology — "Data Analysis" (meta-analysis performed only for outcomes reported by ≥2 clinically comparable studies) and Results — opening paragraph of the ORR subsection (p. 46), which states that pooling was restricted to the clinical evidence and, within it, to KRAS G12C single-arm early-phase datasets reporting the same dichotomous outcomes, and identifies the embedded clinical cohort in Dilly et al. [22] as the third**

			**contributor to the disease-control pool. Study characteristics are tabulated in Table 4 (clinical) and Table 5 (experimental); Table 6 reports the experimental conclusions re-derived after stratification by level of model validation.**
	13b	Describe any methods required to prepare the data for presentation or synthesis, such as handling of missing summary statistics, or data conversions.	**pp. 8, 17-18 — Methodology — "Data Analysis": logit transformation of single-arm proportions with back-transformation for reporting. Overlapping patient populations were assessed for cohort overlap and duplicate data excluded from quantitative synthesis ("Exclusion Criteria"). No summary statistic was imputed; where a trial reported an outcome only for a broader safety population, that denominator is reported as such rather than rescaled, and the effect of rescaling is examined by sensitivity analysis (p. 50).**
	13c	Describe any methods used to tabulate or visually display results of individual studies and syntheses.	**p. 17; Tables 4-10, pp. 24-45; Figures 4-7, pp. 48-50 — Methodology — "Data Analysis": forest plots for pooled estimates, each showing the individual trial proportions with exact 95% CIs and weights alongside the random-effects estimate; ROBINS-I traffic-light and summary plots. Individual study results are tabulated in Tables 4-10. Funnel plots are not used (see item 14).**
	13d	Describe any methods used to synthesize results and provide a rationale for the choice(s). If meta-analysis was performed, describe the model(s), method(s) to identify the presence and extent of statistical heterogeneity, and software package(s) used.	**pp. 17-18; Suppl. S4, Section 3 — Methodology — "Data Analysis": inverse-variance random-effects model, DerSimonian-Laird estimator of τ^2, with Hartung-Knapp adjustment of confidence intervals. Heterogeneity quantified by Cochran's Q, I^2 and τ^2. Prediction intervals were pre-specified but are not reported, because with three contributing studies they span essentially the whole parameter space; this is stated in the Methods. Outcomes not amenable to pooling were synthesised narratively using thematic analysis. Analyses were conducted in Comprehensive Meta-Analysis v3 and independently reproduced in R (verified on R 4.5.2 with the meta package 8.5.0); the code is in Supplementary File S4, Section 3.**
	13e	Describe any methods used to explore possible causes of heterogeneity among study results (e.g. subgroup analysis, meta-regression).	**pp. 17-18; p. 47; p. 55 — Methodology — "Data Analysis" and Results — ORR subsection: leave-one-out sensitivity analysis was used to identify the source of heterogeneity in the ORR pool. Subgroup analysis and meta-regression were not performed; with three contributing studies neither is estimable. This is stated in Methods ("Data Analysis") and in Study Strengths and Limitations (p. 55). Heterogeneity in the experimental literature was addressed instead by the post-hoc stratification by level of model validation (Table 6, p. 35), which is a stratified re-derivation of the narrative conclusions and not a subgroup analysis of a pooled estimate.**
	13f	Describe any sensitivity analyses conducted to assess robustness of the synthesized results.	**pp. 17-18; pp. 47-50; Figure 6, p. 50 — Methodology — "Data Analysis" and Results. Four sensitivity analyses are reported: (i) leave-one-out for the ORR pool (range 31.8-46.3%, p. 47); (ii) leave-one-out for the DCR pool (range 83.3-88.4%, Figure 6); (iii) a post-hoc analysis adding the early-phase D3S-001 PDAC cohort to the ORR pool (40.3%, 15.6-71.2%, p. 47), declared as post-hoc; and (iv) two analyses of the glecirasib grade ≥3 TRAE denominator — rescaling the event count to the 32-patient PDAC subset (22.9%, 10.1-43.8%) and removing the dataset entirely (19.7%, k = 2) — reported on p. 50. All four are reproduced by the code in Supplementary File S4, Section 3.**
	14	Describe any methods used to assess risk of bias due to missing results in a synthesis (arising from reporting biases).	**p. 18; p. 55; Suppl. S3 — Methodology — "Data Analysis": funnel plots were not produced and no formal test of funnel-plot asymmetry was applied. With three studies contributing to each pooled outcome, neither visual inspection nor a statistical test can distinguish asymmetry from chance, and the conventional minimum of ten studies was not approached for any outcome. Risk of bias due to missing results in the clinical literature therefore could not be assessed and is**

			**reported as unexamined rather than as excluded (Study Strengths and Limitations, p. 55). Publication bias in the preclinical literature is discussed separately in the Discussion (p. 55).**
	15	Describe any methods used to assess certainty (or confidence) in the body of evidence for an outcome.	**p. 18; Suppl. S3 — Methodology — "Data Analysis". Certainty of evidence was assessed using GRADE [61] across the domains of risk of bias, inconsistency, indirectness, imprecision and publication bias; the resulting assessments are presented in Supplementary File S3. The experimental evidence was not GRADE-rated; it is stratified instead by level of model validation (Table 6; Supplementary File S5).**
RESULTS	16a	Describe the results of the search and selection process, from the number of records identified in the search to the number of studies included in the review, ideally using a flow diagram.	**p. 18; Figure 1, p. 19 — Results — "Study Selection and Screening" and Figure 1 (PRISMA 2020 flow diagram, two identification streams). Original search: 275 records identified; 3 duplicates and 62 automation-excluded records removed; 210 screened; 166 excluded; 44 full-text reports assessed; 21 excluded (3 inappropriate study design, 3 overlapping patient populations, 15 reporting irrelevant outcomes); 23 studies included. Pre-specified updated search: 13 records identified; 2 duplicates of already-included studies removed; 11 screened; 6 excluded; 5 full-text reports assessed; 4 excluded at eligibility with reasons; 1 study included (garsorasib [44]). Total included: 24 studies (20 experimental and 4 early-phase clinical trials).**
	16b	Cite studies that might appear to meet the inclusion criteria, but which were excluded, and explain why they were excluded.	**Figure 1, p. 19; Suppl. S2, Section 3; p. 54 — Figure 1 names the four reports excluded at the eligibility stage of the updated search with the reason for each: the two 2026 daraxonrasib trials [50,51] (multi-selective RAS(ON) inhibitor rather than an allele-selective agent, and a randomised design that cannot be pooled as a single-arm proportion), the D3S-001 trial (PDAC subset of 4 patients, below the minimum considered informative; retained only for a post-hoc sensitivity analysis) and the siG12D-LODER trial (an siRNA implant with chemotherapy in locally advanced disease). Supplementary File S2, Section 3 gives full citations and reasons for these and for the other full-text reports most likely to appear eligible to a reader. The daraxonrasib trials are additionally discussed in the Discussion (p. 54).**
	17	Cite each included study and present its characteristics.	**p. 23; Table 4, pp. 24-26; Table 5, pp. 27-34 — Results — "Overview of Study Characteristics"; Table 4 (the four included clinical trials) and Table 5 (the 20 included experimental studies, with the highest level of model validation each achieved). All 24 included studies are cited as references [21-44].**
	18	Present assessments of risk of bias for each included study.	**pp. 20-22; Figures 2-3, pp. 20-21; Table 3, p. 22; Suppl. S2, Section 2 — Results — "Risk of Bias Assessment"; Figures 2 and 3 (ROBINS-I traffic-light and summary plots for the four included trials and the embedded clinical cohort), Table 3 (JBI item-level judgements for the 20 experimental studies). Full item-level matrices are in Supplementary File S2, Section 2. The narrative and the figures are harmonised on domain D4, which is judged low for all four trials (p. 20).**
	19	For all outcomes, present, for each study: (a) summary statistics for each group (where appropriate) and (b) an effect estimate and its precision (e.g. confidence/credible interval), ideally using structured tables or plots.	**pp. 37-45; Tables 7-10; Figures 4, 5 and 7, pp. 48-50 — Results — clinical and in vitro subsections; Tables 7-10 and Figures 4, 5 and 7. Every forest plot displays each study's proportion with an exact 95% CI and its weight alongside the pooled estimate, and the same per-study proportions and intervals are quoted in the text (pp. 47-50).**
	20a	For each synthesis, briefly summarise the characteristics and risk of bias among contributing studies.	**p. 46; Table 4, pp. 24-26; pp. 20-22; Suppl. S3 — Results — opening paragraph of the ORR subsection (p. 46). The ORR and grade ≥3 TRAE pools draw on three single-arm early-phase trials [29,40,44], all at moderate overall risk of bias under**

			**ROBINS-I. The DCR pool substitutes the embedded clinical cohort of Dilly et al. [22] for the garsorasib trial; that cohort is at serious overall risk of bias, as stated in Results ("Risk of Bias Assessment", p. 20) and carried into the GRADE rating in Supplementary File S3.**
	20b	Present results of all statistical syntheses conducted. If meta-analysis was done, present for each the summary estimate and its precision (e.g. confidence/credible interval) and measures of statistical heterogeneity. If comparing groups, describe the direction of the effect.	**pp. 47-50 — Results. ORR: 36.9% (95% CI 9.8-75.7), I^2=66.2%, τ^2=0.30, Q=5.92, df=2, p=0.052 (Figure 4, p. 48). DCR: 85.9% (62.1-95.8), I^2=0%, Q=1.91, df=2, p=0.384 (Figure 5, p. 49). Grade ≥3 TRAEs: 23.8% (11.2-43.5), I^2=0%, Q=1.80, df=2, p=0.406 (Figure 7, p. 50). Each is reported with the contributing per-study proportions, their exact 95% CIs and their weights, and is described as an exploratory description of observed activity rather than a comparative estimate of efficacy.**
	20c	Present results of all investigations of possible causes of heterogeneity among study results.	**p. 47 — Results — ORR subsection: leave-one-out analysis attributed the ORR heterogeneity to the lower response in the sotorasib PDAC cohort; its exclusion raised the pooled ORR to 46.3% (37.6-55.2) and removed heterogeneity (I^2=0%). With three studies the source of heterogeneity could not be investigated by subgroup analysis or meta-regression.**
	20d	Present results of all sensitivity analyses conducted to assess the robustness of the synthesized results.	**pp. 47-50; Figure 6, p. 50 — Results — "Leave-One-Out Sensitivity Analysis of DCR" (range 83.3-88.4%; Figure 6); the post-hoc ORR sensitivity analysis adding the D3S-001 cohort (40.3%, 15.6-71.2; I^2=61.7%, p. 47); and the two safety analyses of the glecirasib denominator (22.9%, 10.1-43.8 when rescaled to the 32-patient PDAC subset; 19.7% when the dataset is removed, p. 50).**
	21	Present assessments of risk of bias due to missing results (arising from reporting biases) for each synthesis assessed.	**p. 18; p. 55; Suppl. S3 — Not assessed for any synthesis. Funnel plots were not produced and no asymmetry-based method was applied, because fewer than ten studies contribute to any pooled outcome; this is stated in Methods ("Data Analysis", p. 18), recorded as "not assessed" in the publication-bias column of the GRADE table (Supplementary File S3), and reported in Study Strengths and Limitations (p. 55) as unexamined rather than excluded.**
	22	Present assessments of certainty (or confidence) in the body of evidence for each outcome assessed.	**Suppl. S3; p. 46; p. 55 — Supplementary File S3 (GRADE summary-of-findings table). Certainty was rated very low for the objective response rate, the disease control rate and grade ≥3 treatment-related adverse events. The corresponding statement appears in Results (opening paragraph of the ORR subsection, p. 46) and in Study Strengths and Limitations (p. 55).**
DISCUSSION	23a	Provide a general interpretation of the results in the context of other evidence.	**pp. 50-55 — Discussion, including the asymmetry of clinical maturity between KRAS G12C and KRAS G12D (p. 51), the translational limitations of conventional cell-line models (p. 51), the pharmacological achievability of the in vitro concentrations (p. 52) and the comparison with the 2026 daraxonrasib trials (p. 54).**
	23b	Discuss any limitations of the evidence included in the review.	**p. 55 — Discussion — "Study Strengths and Limitations": predominance of early-phase single-arm trials with small samples; a pooled outcome dependent on a retrospectively assembled cohort at serious risk of bias; a partly non-PDAC-specific safety denominator; heterogeneity of the experimental models in kind and in translational validity, with 6 of 20 studies contributing no in vivo evidence; in vitro concentrations above achievable clinical exposure; and likely preclinical publication bias.**
	23c	Discuss any limitations of the review processes used.	**p. 55 — Discussion — "Study Strengths and Limitations": absence of prospective registration, restriction to English-language publications, the inability to conduct subgroup analysis or meta-regression at three studies per outcome, the inability to**

			**assess publication bias, and the exploratory status of the pooled estimates.**
	23d	Discuss implications of the results for practice, policy and future research.	**pp. 56-57 — Discussion — “Study Implications” and “Future Research Directions”; Conclusion (p. 57).**
OTHER INFORMATION	24a	Provide registration information for the review, including register name and registration number, or state that the review was not registered.	**pp. 5-6 — Methodology — “Protocol and Registration”: the review was not prospectively registered. The justification (a substantial preclinical component placing the review outside PROSPERO scope) is given in the same subsection, together with an explicit statement that references elsewhere to a “pre-specified” search or analysis refer to the internal protocol and not to a public registration.**
	24b	Indicate where the review protocol can be accessed, or state that a protocol was not prepared.	**pp. 5-6 — Methodology — “Protocol and Registration”: an a priori internal protocol specifying the PICOS criteria, search strategy, appraisal tools and planned synthesis was prepared and dated before screening began but not published; it is available from the corresponding author on request.**
	24c	Describe and explain any amendments to information provided at registration or in the protocol.	**p. 6; p. 46; Suppl. S2, Section 1.5 — Two amendments to the a priori protocol are documented, and are named individually in “Protocol and Registration”. Amendment 1: a pre-specified updated search using a gap-corrected drug block (June 2026), which identified one further eligible KRAS G12C trial, garsorasib [44]; the amended block and the resulting stream are given in Supplementary File S2, Section 1.5, and the stream is shown separately in Figure 1. Amendment 2: the protocol restricted pooling to early-phase trials, but the KRAS G12C PDAC cohort embedded in Dilly et al. [22] was admitted to the disease-control pool so that the outcome had three contributors rather than two, the Hartung-Knapp interval being uninformative at two studies; that cohort is retrospective and is rated at serious risk of bias accordingly. In addition, two analyses added in response to peer review — the classification of experimental model validation and the appraisal of pharmacological achievability — are declared as post-hoc analyses rather than protocol amendments, and are identified as such wherever they are described (pp. 6, 10, 35, 52). The post-hoc ORR sensitivity analysis adding the D3S-001 cohort is likewise labelled post-hoc (p. 47).**
	25	Describe sources of financial or non-financial support for the review, and the role of the funders or sponsors in the review.	**p. 58 — Funding statement: “This research received no external funding.”**
	26	Declare any competing interests of review authors.	**p. 58 — Conflicts of Interest statement: “The authors declare no conflicts of interest.”**
	27	Report which of the following are publicly available and where they can be found: template data collection forms; data extracted from included studies; data used for all analyses; analytic code; any other materials used in the review.	**p. 58; Suppl. S2, Section 2; Suppl. S4; Suppl. S5 — Data Availability Statement. The data extraction template and the study-level dataset used for every pooled analysis are in Supplementary File S4, Sections 1 and 2 (the dataset is also supplied as S4_study_level_dataset.csv); the R code reproducing every pooled estimate, per-study exact confidence interval, leave-one-out analysis, sensitivity analysis and forest plot is in Section 3 of the same file and is supplied as S4_analysis_code.R. The item-level risk-of-bias matrices are in Supplementary File S2, Section 2. The per-study model-validation matrix and the pharmacological-achievability appraisal are in Supplementary File S5. No other materials were used.**

All 27 items of the PRISMA 2020 checklist are addressed. Supplementary Files S2 (search strategies for both identification streams and item-level risk-of-bias assessments), S3 (GRADE certainty of evidence), S4 (study-level dataset, extraction template and analytic code) and S5 (per-study model-validation matrix and pharmacological-achievability appraisal) accompany this checklist.
