# Supplementary material for: Allele-Stratified Activity of RAS Inhibitors in Pancreatic Ductal Adenocarcinoma: A Systematic Review and Exploratory Meta-Analysis
Source: Cancers (Basel). 2026 Sep 28;18(19):3139. doi: 10.3390/cancers18193139 (PMC13664068; doi:10.3390/cancers18193139)
Supplement: Supplementary file 1 [file cancers-18-03139-s001.zip › Supplementary_File S2_Search_Strategies_and_RoB.pdf]

Supplementary File S2. Search Strategies and Item-Level Risk-of-Bias Assessments

Allele-stratified activity of RAS inhibitors in pancreatic ductal adenocarcinoma: a systematic review with model-validation-stratified synthesis of preclinical evidence and exploratory meta-analysis of early-phase trials — Manuscript ID cancers-4500792

1. Electronic search strategies

Records were identified through two streams, both of which are shown separately in Figure 1 of the main text. The original search (Sections 1.1–1.4) comprised three Boolean concept blocks combined with AND: (1) named RAS- and KRAS-targeted agents; (2) RAS codon substitutions; (3) pancreatic ductal adenocarcinoma, and was run in four databases from inception to 26 June 2026. The pre-specified updated search (Section 1.5) re-ran an amended intervention block in June 2026; it was a PubMed search with targeted registry verification rather than a full four-database re-run, and is reported as a distinct stream so that its narrower coverage is transparent. No language filter was applied at the search stage; non-English reports were excluded at screening. Studies identified by either stream were screened, appraised and counted under identical criteria.

1.1 PubMed

Block #1 — intervention:

```
("Sotorasib" OR "AMG 510" OR "Adagrasib" OR "MRTX849" OR "Olomorasib" OR
 "LY3537982" OR "Divarasib" OR "GDC-6036" OR "Glecirasib" OR "JAB-21822" OR
 "Zoldonrasib" OR "RMC-9805" OR "MRTX1133" OR "VS-7375" OR "GFH375" OR
 "Setidegrasib" OR "Daraxonrasib" OR "RMC-6236" OR "RMC-7977" OR "BI-2865" OR
 "BI-2493" OR "LY4066434")
```

Block #2 — allele:

```
(G12D OR G12V OR G12R OR G12C OR G12A OR G12S OR G13D OR Q61H OR Q61R OR
 Q61L OR K117N OR A146T OR A146V OR A146P)
```

Block #3 — disease:

```
("Pancreatic Ductal Adenocarcinoma" OR PDAC OR "Pancreatic Adenocarcinoma" OR
 "Pancreatic Cancer" OR "Pancreatic Carcinoma" OR "Pancreatic Neoplasm" OR
 "Pancreatic Tumor" OR "Pancreatic Malignancy")
```

Final query: #1 AND #2 AND #3. Block yields: #1 = 1,007; #2 = 6,218; #3 = 88,189; combined = 117 records.

1.2 Cochrane Library (Trials)

Searched in Title, Abstract and Keywords, restricted to the Trials register:

```
#1  ("Sotorasib" OR "AMG 510" OR "Adagrasib" OR "MRTX849" OR "Olomorasib" OR
     "LY3537982" OR "Divarasib" OR "GDC-6036" OR "Glecirasib" OR "JAB-21822" OR
     "Zoldonrasib" OR "RMC-9805" OR "MRTX1133" OR "VS-7375" OR "GFH375" OR
     "Setidegrasib" OR "Daraxonrasib" OR "RMC-6236" OR "RMC-7977" OR "BI-2865" OR
     "BI-2493" OR "LY4066434"):ti,ab,kw
#2  (G12D OR G12V OR G12R OR G12C OR G12A OR G12S OR G13D OR Q61H OR Q61R OR
     Q61L OR K117N OR A146T OR A146V OR A146P):ti,ab,kw
#3  ("Pancreatic Ductal Adenocarcinoma" OR PDAC OR "Pancreatic Adenocarcinoma" OR
     "Pancreatic Cancer" OR "Pancreatic Carcinoma" OR "Pancreatic Neoplasm" OR
     "Pancreatic Tumor" OR "Pancreatic Malignancy"):ti,ab,kw
#4  #1 AND #2 AND #3      [Trials register only]
```

Yield: 3 records.

1.3 Scopus

```
( TITLE-ABS-KEY ( ( "Sotorasib" OR "AMG 510" OR "Adagrasib" OR "MRTX849" OR
  "Olomorasib" OR "LY3537982" OR "Divarasib" OR "GDC-6036" OR "Glecirasib" OR
  "JAB-21822" OR "Zoldonrasib" OR "RMC-9805" OR "MRTX1133" OR "VS-7375" OR
  "GFH375" OR "Setidegrasib" OR "Daraxonrasib" OR "RMC-6236" OR "RMC-7977" OR
  "BI-2865" OR "BI-2493" OR "LY4066434" ) )
AND ( TITLE-ABS-KEY ( ( G12D OR G12V OR G12R OR G12C OR G12A OR G12S OR G13D OR
  Q61H OR Q61R OR Q61L OR K117N OR A146T OR A146V OR A146P ) ) )
AND ( TITLE-ABS-KEY ( ( "Pancreatic Ductal Adenocarcinoma" OR PDAC OR
  "Pancreatic Adenocarcinoma" OR "Pancreatic Cancer" OR "Pancreatic Carcinoma" OR
  "Pancreatic Neoplasm" OR "Pancreatic Tumor" OR "Pancreatic Malignancy" ) ) )
```

Yield: 103 records.

1.4 Google Scholar

Google Scholar does not accept Boolean strings of the length used in the other databases, so the three concept blocks were entered as simplified keyword combinations across all fields:

```
sotorasib OR adagrasib OR glecirasib OR garsorasib OR MRTX1133 OR setidegrasib
  OR daraxonrasib "pancreatic" KRAS G12C
sotorasib OR adagrasib OR glecirasib OR MRTX1133 OR setidegrasib
  "pancreatic ductal adenocarcinoma" KRAS G12D
"KRAS inhibitor" OR "RAS inhibitor" "pancreatic ductal adenocarcinoma"
  "objective response" OR "disease control"
```

Consistent with recommended methodological practice for supplementary grey-literature searching, the first 200 records ranked by relevance were screened for each combination. Yield carried forward after relevance screening: 52 records.

1.5 Pre-specified updated search — second identification stream (June 2026)

During preparation of the review, several agents with newly reported PDAC clinical data were found to be absent from the intervention block specified in the a priori protocol. An amended block was constructed and re-run in June 2026; this is the first of the two documented amendments to the a priori protocol (PRISMA item 24c) and is reported as a distinct identification stream in Figure 1. The stream identified 13 records, of which 2 were duplicates of studies already included; 11 were screened, 6 were excluded, and 5 reports were assessed for eligibility. One met the eligibility criteria — the KRAS G12C trial of garsorasib (Yamamoto et al., reference [44]) — and is a fully included study of this review, bringing the total to 24 studies; it contributes to the objective response and grade ≥3 TRAE pools. The four reports excluded at eligibility, with the reason for each, are shown in Figure 1 and listed in Section 3 below.

Corrected Block #1 (additions in the final line):

```
("Sotorasib" OR "AMG 510" OR "Adagrasib" OR "MRTX849" OR "Olomorasib" OR
 "LY3537982" OR "Divarasib" OR "GDC-6036" OR "Glecirasib" OR "JAB-21822" OR
 "Zoldonrasib" OR "RMC-9805" OR "MRTX1133" OR "VS-7375" OR "GFH375" OR
 "Setidegrasib" OR "Daraxonrasib" OR "RMC-6236" OR "RMC-7977" OR "BI-2865" OR
 "BI-2493" OR "LY4066434" OR "Garsorasib" OR "D-1553" OR "D3S-001" OR
 "RNK08954" OR "HRS-4642" OR "Opnurasib" OR "JDQ443")
```

1.6 Records identified by source

Source	Fields searched	Date last searched	Records identified
PubMed	All Fields	Inception – 26 June 2026	117
Cochrane Library (Trials)	Title, abstract, keywords	Inception – 26 June 2026	3
Scopus	Title, abstract, keywords	Inception – 26 June 2026	103
Google Scholar	All fields (first 200 records screened)	Inception – 26 June 2026	52
ClinicalTrials.gov / WHO ICTRP	Registry search for ongoing or unpublished studies	26 June 2026	0 additional eligible
Citation searching of eligible studies	Reference lists of included reports	26 June 2026	0 additional eligible
Total records identified — original search	—	—	275
Pre-specified updated search (PubMed + targeted registry verification; not a full four-database re-run)	All Fields	June 2026	13
Total records identified — both streams	—	—	288

2. Item-level risk-of-bias assessments

2.1 ROBINS-I — non-randomised clinical studies

D1 confounding; D2 selection of participants; D3 classification of interventions; D4 deviations from intended interventions; D5 missing outcome data; D6 measurement of outcomes; D7 selection of the reported result. Two assessors judged each domain independently; disagreements were resolved by discussion with a third reviewer.

Study	D1	D2	D3	D4	D5	D6	D7	Overall
Strickler et al., 2023 [40] — sotorasib, CodeBreaK 100	Moderate	Low	Low	Low	Low	Low	Moderate	Moderate
Li et al., 2025 [29] — glecirasib	Moderate	Low	Low	Low	Low	Low	Moderate	Moderate
Park et al., 2026 [35] — setidegrasib	Moderate	Low	Low	Low	Low	Low	Moderate	Moderate
Yamamoto et al., 2025 [44] — garsorasib (D-1553)	Moderate	Low	Low	Low	Low	Low	Moderate	Moderate
Dilly et al., 2024 [22] — embedded KRAS G12C PDAC clinical cohort	Serious	Serious	Moderate	Moderate	Moderate	Serious	Moderate	Serious

For the four included trials, the moderate overall judgement is driven by D1 and D7: single-arm early-phase designs cannot control for prognostic imbalance, and none reported against a publicly registered analysis plan covering the PDAC subgroup. All four are judged low risk in D2 to D6, including deviations from intended interventions (D4), reflecting protocol-defined dosing with dose modifications specified in advance in each report; this judgement is stated explicitly here and in the main text because it was omitted from the narrative of the submitted version while appearing in the corresponding figure. Garsorasib [44] is appraised here as a fully included trial rather than as an addendum. The judgements in this table are those plotted in Figures 2 and 3 of the main text.

The fifth row requires explanation. Dilly et al. [22] is principally a resistance-mechanism study, and its experimental component is appraised under the JBI checklist in Section 2.2 below. Embedded within it, however, is a cohort of 22 patients with KRAS G12C PDAC treated with adagrasib or sotorasib and assessed for disease control; that cohort contributes to the pooled disease control rate and therefore requires a design-appropriate clinical appraisal, which is given here. It is rated at serious overall risk of bias. The cohort was assembled retrospectively for genomic profiling at acquired resistance rather than enrolled prospectively, so neither prognostic balance nor the selection of participants can be established (D1, D2, both serious). Disease control was not defined against a protocol-specified assessment window, unlike the two trials it is pooled with (D6, serious). The two agents received were recorded from clinical records rather than assigned under protocol (D3), dose modification was not protocol-governed (D4), completeness of follow-up is not reported against a fixed denominator (D5) and no pre-specified analysis plan exists for the cohort (D7); these four domains are rated moderate. Because some sotorasib-treated patients in this cohort may also be represented in CodeBreaK 100 [40], the possibility of partial population overlap between two contributors to the disease control pool cannot be excluded from the published reports. This is carried into the GRADE rating for disease control in Supplementary File S3 and is stated as a limitation in the main text.

The two 2026 daraxonrasib trials were excluded at the eligibility stage of the updated search, with the reason recorded in Figure 1: daraxonrasib is a multi-selective RAS(ON) inhibitor active across RAS G12 variants rather than an allele-selective agent, and pooling the randomised trial as a single-arm proportion would discard its concurrent control arm. They were therefore not appraised here, although they are discussed in the main text; the randomised RASolute 302 trial would in any case require RoB 2 rather than ROBINS-I.

2.2 Modified JBI checklist — experimental studies

Q1 temporal sequence of cause and effect; Q2 baseline comparability of experimental models; Q3 appropriate control groups; Q4 multiple outcome measurements; Q5 completeness of follow-up; Q6 identical outcome assessment across groups; Q7 reliability of outcome measurement; Q8 appropriate statistical analysis; Q9 clear and consistent description of the intervention. NA denotes purely in vitro studies without longitudinal follow-up, for which Q5 does not apply.

Study	Q1	Q2	Q3	Q4	Q5	Q6	Q7	Q8	Q9	Overall
Abuelafia et al. (2025) [21]	Yes	Yes	Yes	Yes	NA	Yes	Yes	Yes	Yes	Include
Dilly et al. (2024) [22]	Yes	Unclear	Yes	Yes	Yes	Yes	Yes	Yes	Yes	Include
Ghanwatkar et al. (2025) [23]	Yes	Yes	Yes	Yes	Yes	Yes	Yes	Yes	Yes	Include
Hallin et al. (2022) [24]	Yes	Yes	Yes	Yes	Yes	Yes	Yes	Yes	Yes	Include
Han et al. (2024) [25]	Yes	Yes	Yes	Yes	Yes	Yes	Yes	Yes	Yes	Include
Jesenko et al. (2025) [26]	Yes	Yes	Yes	Unclear	NA	Yes	Yes	Yes	Yes	Include
Jiang et al. (2025) [27]	Yes	Yes	Yes	Yes	Yes	Yes	Yes	Yes	Yes	Include
Kumarasamy et al. (2024) [28]	Yes	Yes	Yes	Yes	Yes	Yes	Yes	Yes	Yes	Include
Ma et al. (2022) [30]	Yes	Unclear	Yes	Yes	NA	Yes	Yes	Yes	Yes	Include
Mahadevan et al. (2023) [31]	Yes	Yes	Yes	Yes	Yes	Yes	Yes	Yes	Yes	Include
Miyazaki et al. (2024) [32]	Yes	Yes	Yes	Yes	Yes	Yes	Yes	Yes	Yes	Include
Molnár et al. (2024) [33]	Yes	Unclear	Yes	Yes	NA	Yes	Yes	Yes	Yes	Include
Ozawa et al. (2025) [34]	Yes	Yes	Yes	Yes	Yes	Yes	Yes	Yes	Yes	Include
Paulsohn et al. (2025) [36]	Yes	Yes	Yes	Yes	Yes	Yes	Yes	Yes	Yes	Include
Principe et al. (2026) [37]	Yes	Yes	Yes	Yes	Yes	Yes	Yes	Yes	Yes	Include
Ramalingam et al. (2025) [38]	Yes	Yes	Yes	Unclear	NA	Yes	Yes	Yes	Yes	Include
Song et al. (2024) [39]	Yes	Yes	Yes	Yes	Yes	Yes	Yes	Yes	Yes	Include
Szigeti et al. (2026) [41]	Yes	Yes	Yes	Yes	Yes	Yes	Yes	Yes	Yes	Include

Tajiknia et al. (2024) [42]	Yes	Unclear	Yes	Yes	NA	Yes	Yes	Yes	Yes	Include
Xu et al. (2026) [43]	Yes	Yes	Yes	Yes	Yes	Yes	Yes	Yes	Yes	Include

This table reproduces manuscript Table 3 item for item. Six Unclear judgements were recorded: baseline comparability of experimental models (Q2) in Dilly [22], Ma [30], Molnár [33] and Tajiknia [42], and repeated outcome measurement (Q4) in Jesenko [26] and Ramalingam [38]. No study was judged No on any item, and all 20 met the threshold for inclusion.

3. Reports assessed at full text and excluded, with reasons

In the original search stream, 44 reports were assessed at full text and 23 were included; in the updated search stream, 5 reports were assessed and 1 (garsorasib [44]) was included, giving 24 included studies. The table below lists the eight reports most likely to appear eligible to a reader, together with the reason each was not carried into the synthesis (PRISMA item 16b); the first four are the reports excluded at the eligibility stage of the updated search and appear individually in Figure 1. The remaining full-text exclusions were on grounds already stated in the Exclusion Criteria — publication type, absence of PDAC, absence of a RAS-targeted intervention, or absence of an efficacy outcome — and are counted in the flow diagram (Figure 1).

Report	Design	Reason not included
Wolpin BM, et al. Daraxonrasib in previously treated metastatic pancreatic ductal adenocarcinoma (RMC-6236-001). N Engl J Med 2026.	Phase 1/2 trial	Daraxonrasib is a RAS(ON) multi-selective inhibitor acting across RAS G12 variants rather than on a single allele, and therefore falls outside the allele-stratified synthesis. Discussed narratively in the Discussion.
O'Reilly EM, et al. Daraxonrasib versus standard of care in previously treated metastatic pancreatic ductal adenocarcinoma (RASolute 302). N Engl J Med 2026.	Randomised phase 3 trial	Randomised, controlled design. Pooling it as a single-arm proportion would discard the control arm and misrepresent the evidence. Discussed narratively in the Discussion.
Cho BC, et al. D3S-001, a KRAS G12C inhibitor, in advanced solid tumours. Nat Med 2025;31:1524–1533. doi:10.1038/s41591-025-03688-6	Phase 1 trial	Only four PDAC patients contributed, below the minimum considered informative for single-arm estimation. Excluded at the eligibility stage and retained solely for a post-hoc sensitivity analysis of the ORR pool, reported as such.
Alagesan B, et al. A phase II trial of an extended-release siRNA implant targeting KRAS G12D/V in locally advanced pancreatic ductal adenocarcinoma (siG12D-LODER). Clin Cancer Res 2026. doi:10.1158/1078-0432.CCR-25-3189	Phase 2 trial (one randomised and one single-arm cohort)	An siRNA-based local delivery system combined with chemotherapy in locally advanced disease, rather than a systemically administered allele-selective RAS inhibitor in advanced or metastatic PDAC. Excluded at the eligibility stage of the updated search.
Xie W, et al. RNK08954, a KRAS G12D inhibitor, in advanced solid tumours. Cancer Discov 2026. doi:10.1158/2159-8290.CD-25-1346	Phase Ia trial	Published after the 26 June 2026 search cut-off. Identified by the updated search and noted, but not eligible for the formal review.
Leng X, et al. HRS-4642 plus nimotuzumab in KRAS G12D-mutant advanced solid tumours: a study protocol. Front Pharmacol 2025;16:1562481. doi:10.3389/fphar.2025.1562481	Trial protocol	Protocol with no outcome data (NCT06773130). Excluded under the study-protocol exclusion criterion.
Aslani S, et al. Garsorasib in KRAS G12C-mutant pancreatic cancer: two cases. Front Oncol 2025;15:1691760. doi:10.3389/fonc.2025.1691760	Case report (2 patients)	Excluded under the case-report exclusion criterion.
Xue J, et al. Resistance mechanisms to HRS-4642 plus nimotuzumab in KRAS G12D-mutant models. Cancer Lett 2026;633:218684. doi:10.1016/j.canlet.2026.218684	Preclinical	Reported no allele-stratified efficacy outcome that was not already covered by an included experimental study of the same agent class.
