# Supplementary material for: Allele-Stratified Activity of RAS Inhibitors in Pancreatic Ductal Adenocarcinoma: A Systematic Review and Exploratory Meta-Analysis
Source: Cancers (Basel). 2026 Sep 28;18(19):3139. doi: 10.3390/cancers18193139 (PMC13664068; doi:10.3390/cancers18193139)
Supplement: Supplementary file 1 [file cancers-18-03139-s001.zip › Supplementary_File S3_GRADE_Certainty.pdf]

Supplementary File S3. GRADE Certainty of Evidence

Allele-stratified activity of RAS inhibitors in pancreatic ductal adenocarcinoma — Manuscript ID cancers-4500792

Certainty was assessed with GRADE [61] for the three outcomes that were quantitatively synthesised. All contributing datasets are single-arm early-phase trials, except the retrospectively assembled clinical cohort that contributes to disease control, so every outcome starts at low certainty for observational evidence and is rated further down for imprecision arising from the small number of contributing datasets. No outcome was rated up. The estimates below correspond to the three-study pools reported in the revised manuscript (Figures 4, 5 and 7). The publication-bias domain is not assessed for any outcome: funnel plots were not produced, because with three studies per outcome neither visual inspection nor a formal asymmetry test can distinguish asymmetry from chance. Publication bias in the clinical literature is therefore unexamined rather than excluded.

Outcome	Studies (design)	Risk of bias	Inconsistency	Indirectness	Imprecision	Publication bias	Pooled effect (95% CI)	Certainty
Objective response rate (ORR)	3 single-arm phase I/II trials [29,40,44]	Serious — open-label, non-randomised, single-arm designs; ROBINS-I overall moderate, with residual confounding (D1) and selective-reporting concern (D7) in every trial	Serious — $I^2 = 66.2\%$, $\tau^2 = 0.30$; leave-one-out shows the sotorasib PDAC cohort drives the dispersion	Not serious — directly assessed KRAS G12C inhibitors in advanced PDAC using RECIST-defined response	Serious — three studies, pooled 95% CI spans 9.8–75.7%	Not assessed — funnel plots were not produced and no formal asymmetry test was applied; fewer than ten studies contribute to any outcome, so no funnel-based method is interpretable. Publication bias is unexamined rather than excluded.	36.9% (95% CI 9.8–75.7); ≈369 responders per 1,000 treated	⨁○○○ Very low
Disease control rate (DCR)	3 single-arm datasets [22,29,40]	Very serious — two open-label uncontrolled trials at moderate risk under ROBINS-I, plus one retrospective convenience cohort [22] at serious risk in which disease control was not protocol-defined and partial population overlap with [40] cannot be excluded	Not serious — $I^2 = 0\%$, $\tau^2 = 0$; leave-one-out range 83.3–88.4%	Not serious — RECIST-assessed disease control after KRAS G12C inhibition in PDAC	Serious — three studies; pooled 95% CI 62.1–95.8%	Not assessed — funnel plots were not produced and no formal asymmetry test was applied; fewer than ten studies contribute to any outcome, so no funnel-based method is interpretable. Publication bias is unexamined rather than excluded.	85.9% (95% CI 62.1–95.8); ≈859 with disease control per 1,000 treated	⨁○○○ Very low
Grade ≥3 treatment-related adverse	3 single-arm phase I/II trials	Serious — open-label safety reporting without comparator arms; one	Not serious — $I^2 = 0\%$, $Q = 1.80$, $df = 2$, $p =$	Serious — the glecirasib safety	Serious — three studies; pooled 95%	Not assessed — funnel plots were	23.8% (95% CI 11.2–43.5); ≈238 per 1,000	⨁○○○ Very low

events	[29,40,44]	denominator reflects the broader solid-tumour safety population rather than the PDAC efficacy subset	0.406	denominator (15/54) is the broader KRAS G12C solid-tumour population, not the 32-patient PDAC subset. A sensitivity analysis rescaling the glecirasib event count to that subset gave 22.9% (10.1–43.8; $I^2 = 0\%$), and removing the dataset entirely gave 19.7% from two trials, so the pooled estimate is insensitive to the denominator; the indirectness of the population is not thereby removed.	CI 11.2–43.5%	not produced and no formal asymmetry test was applied; fewer than ten studies contribute to any outcome, so no funnel-based method is interpretable. Publication bias is unexamined rather than excluded.	treated	

⨁○○○ Very low: the estimate is very uncertain, and the true effect is likely to be substantially different. All three quantitatively synthesised outcomes are rated very low, and the revised manuscript describes them as exploratory descriptions of observed activity rather than as comparative estimates of treatment efficacy throughout. Time-to-event outcomes (progression-free and overall survival) were not pooled and were therefore not GRADE-rated; they are reported as study-level medians with 95% confidence intervals in Tables 7 and 8 of the main text. The experimental (preclinical) evidence was synthesised narratively, contributes to no pooled estimate and is not GRADE-rated; it is instead stratified by the level of model validation achieved, in Table 5, Table 6 and Supplementary File S5.
