# Supplementary material for: Allele-Stratified Activity of RAS Inhibitors in Pancreatic Ductal Adenocarcinoma: A Systematic Review and Exploratory Meta-Analysis
Source: Cancers (Basel). 2026 Sep 28;18(19):3139. doi: 10.3390/cancers18193139 (PMC13664068; doi:10.3390/cancers18193139)
Supplement: Supplementary file 1 [file cancers-18-03139-s001.zip › Supplementary_File S4_Dataset_and_Code.pdf]

Supplementary File S4. Data Extraction Template, Study-Level Dataset and Analytic Code

Allele-stratified activity of RAS inhibitors in pancreatic ductal adenocarcinoma: a systematic review with model-validation-stratified synthesis of preclinical evidence and exploratory meta-analysis of early-phase trials — Manuscript ID cancers-4500792

1. Data extraction template

Two reviewers extracted every included study independently onto the form below. Discrepancies were resolved by discussion, with a third reviewer as arbiter. No automation tools were used at this stage, and study investigators were not contacted; all values were taken from the published reports.

Field	Type	Definition and permitted values
Study ID	Free text	First author, year, reference number in this review
Country	Free text	Country or countries in which the work was conducted
Study design	Controlled list	Phase 1 / phase 1-2 / phase 2 trial; retrospective clinical cohort; in vitro; in vivo; ex vivo (PDO, PDX, GEMM)
Sample characteristics	Free text	For clinical studies: number enrolled, number efficacy-evaluable, line of therapy, disease stage. For experimental studies: model system and number of independent replicates
Cell lines / models	Free text	Named human PDAC lines, organoids, xenografts or genetically engineered models
Tumour molecular characteristics	Free text	KRAS or RAS allele(s), co-mutations where reported
Intervention	Free text	Agent(s), dose, schedule, and any combination partner
Target allele	Controlled list	G12C / G12D / G12V / G12R / other / pan-RAS
Comparator	Free text	Standard therapy, placebo, vehicle, alternative targeted agent, or none
Outcome measures	Controlled list	ORR; DCR; PFS; OS; DoR; CBR; grade >=3 TRAEs; IC50; combination index; Bliss score
Numerator and denominator	Numeric	For each dichotomous outcome: events and the denominator reported by the source, with a note stating which population that denominator represents
Effect estimate and precision	Numeric	Median with 95% CI for time-to-event outcomes, as reported
Findings	Free text	One- to two-sentence summary in the authors' own terms
Risk-of-bias tool applied	Controlled list	ROBINS-I (clinical) or modified JBI quasi-experimental checklist (experimental)
Notes on population overlap	Free text	Any indication that participants may also appear in another included report

2. Study-level dataset used for all pooled analyses

Every pooled estimate reported in the manuscript is computed from the eleven study-outcome records below. Each record is a single arm: events is the number of participants with the event, and n is the denominator reported by the source for that outcome. Proportions are given with exact (Clopper–Pearson) 95% confidence intervals, which are the intervals quoted for each trial in the Results and drawn on every forest plot; they are computed from the events and the denominator alone and do not depend on the meta-analysis model. Reference numbers correspond to the manuscript reference list. This table is supplied in machine-readable form as S4_study_level_dataset.csv.

**Study**	**Ref.**	**Drug**	**Allele**	**Outcome**	**Events**	**n**	Proportion (exact 95% CI)
Strickler 2023	40	Sotorasib	G12C	ORR	8	38	21.1% (9.6–37.3)
Li 2025	29	Glecirasib	G12C	ORR	15	32	46.9% (29.1–65.3)
Yamamoto 2025	44	Garsorasib	G12C	ORR	10	22	45.5% (24.4–67.8)
Park 2026	35	Setidegrasib	G12D	ORR	6	31	19.4% (7.5–37.5)
D3S-001 2025	NA	D3S-001	G12C	ORR	3	4	75.0% (19.4–99.4)
Strickler 2023	40	Sotorasib	G12C	DCR	32	38	84.2% (68.7–94.0)
Li 2025	29	Glecirasib	G12C	DCR	30	32	93.8% (79.2–99.2)
Dilly 2024	22	Adagrasib/Sotorasib	G12C	DCR	18	22	81.8% (59.7–94.8)
Strickler 2023	40	Sotorasib	G12C	TRAE_ge3	6	38	15.8% (6.0–31.3)
Li 2025	29	Glecirasib	G12C	TRAE_ge3	15	54	27.8% (16.5–41.6)
Yamamoto 2025	44	Garsorasib	G12C	TRAE_ge3	6	24	25.0% (9.8–46.7)

Notes. (i) D3S-001 (Cho et al., 2025) is not an included study: it was excluded at the eligibility stage of the updated search because its PDAC subset comprised only 4 patients, and it contributes solely to the post-hoc ORR sensitivity analysis. It is marked NA in the reference column. (ii) Park 2026 (setidegrasib, KRAS G12D) is included in the review and reported narratively; it is not pooled, because no second clinically comparable KRAS G12D trial was available. (iii) Dilly 2024 [22] contributes only to the disease-control pool; that cohort was assembled retrospectively and is appraised at serious risk of bias under ROBINS-I in Supplementary File S2, Section 2.1. (iv) The glecirasib grade ≥3 TRAE denominator (n = 54) is the broader KRAS G12C solid-tumour safety population reported by that trial, not the 32-patient PDAC efficacy subset; this is stated in the manuscript and is the basis for the indirectness rating in Supplementary File S3. Two sensitivity analyses quantify its consequence — rescaling the glecirasib event count to the 32-patient subset gives 22.9% (10.1–43.8), and removing the dataset gives 19.7% from two trials — and both are reproduced by the code in Section 3. (v) The 20 experimental (preclinical) studies contribute to no pooled estimate; they are synthesised narratively and stratified by level of model validation in Table 5, Table 6 and Supplementary File S5.

3. Analytic code (R)

The script below reproduces every pooled estimate, per-study exact confidence interval, heterogeneity statistic, leave-one-out analysis, sensitivity analysis and forest plot reported in the manuscript, from the dataset in Section 2. It requires R (≥ 4.4) and the meta package (≥ 6.0), and is supplied as S4_analysis_code.R. It was verified on R 4.5.2 with meta 8.5.0. No funnel plot is produced, because with three studies per outcome no funnel-based method is interpretable. The values the script produces are listed in the closing comment block for direct comparison against the manuscript.

```
################################################################################
# Supplementary File S4, Section 3 - Analytic code
#
# Allele-stratified activity of RAS inhibitors in pancreatic ductal
# adenocarcinoma: a systematic review with model-validation-stratified
# synthesis of preclinical evidence and exploratory meta-analysis of
# early-phase trials
# Manuscript ID: cancers-4500792  (revised version, R1)
#
# This script reproduces every pooled estimate, per-study exact confidence
# interval, heterogeneity statistic, leave-one-out analysis, sensitivity
# analysis and forest plot reported in the manuscript, from the study-level
# dataset in S4_study_level_dataset.csv.
#
# Requirements: R >= 4.4, meta >= 6.0
#   install.packages("meta")
#
# Reproduction:
#   Rscript S4_analysis_code.R
# Outputs: fig4_ORR.png, fig5_DCR.png, fig6_LOO_DCR.png, fig7_TRAE.png,
#          and S4_reproduced_results.csv
#
# Method. Single-arm proportions are pooled on the logit scale with an
# inverse-variance random-effects model (DerSimonian-Laird estimator of tau^2)
# and Hartung-Knapp adjustment of the confidence interval, then
# back-transformed to percentages. Hartung-Knapp is applied to every pooled
# estimate; at k = 3 it uses t with 2 degrees of freedom, which is why the
# intervals are wider than a normal-approximation interval would be. This is
# intentional: the manuscript describes all three pooled estimates as
# exploratory descriptions of observed activity, and the intervals should
# reflect that. Per-study intervals are exact (Clopper-Pearson) and are
# printed on every forest plot, so each pooled estimate is reported alongside
# the individual proportions that produce it.
#
# No funnel plot is produced and no test of funnel-plot asymmetry is applied.
# With three studies contributing to each outcome, neither visual inspection
# nor a formal test can distinguish asymmetry from chance; publication bias in
# the clinical literature is therefore reported as unexamined rather than as
# excluded (see the manuscript, Data Analysis, and Supplementary File S3).
################################################################################

library(meta)
options(stringsAsFactors = FALSE)

DATA <- "S4_study_level_dataset.csv"
dat  <- read.csv(DATA)
stopifnot(all(c("study", "ref", "drug", "allele", "outcome",
                "events", "n") %in% names(dat)))

## ----------------------------------------------------------------------------
## Pool definitions
##
## Each pool names the studies that contribute, so the composition of every
## reported estimate is explicit rather than implied by a filter. The ORR and
## grade >=3 TRAE pools draw on the three single-arm early-phase KRAS G12C
## trials, which are the complete set of eligible PDAC-specific datasets. The
## disease-control pool substitutes the KRAS G12C PDAC cohort embedded in
## Dilly et al. (ref 22) for the garsorasib trial, because garsorasib did not
## report disease control on a comparable basis. That cohort was assembled
## retrospectively and is appraised at serious risk of bias under ROBINS-I
## (Supplementary File S2, Section 2.1); the disease-control estimate is rated
## very low certainty accordingly (Supplementary File S3).
##
## D3S-001 is NOT an included study: it was excluded at the eligibility stage
## because its PDAC subset comprised only 4 patients. It contributes solely to
## the post-hoc ORR sensitivity analysis and is excluded from the primary pools.
## ----------------------------------------------------------------------------

POOLS <- list(
  ORR = list(
```

```
    outcome = "ORR",
    studies = c("Strickler 2023", "Li 2025", "Yamamoto 2025"),
    file    = "fig4_ORR.png",
    label   = "Objective response rate, KRAS G12C inhibitors"),
  DCR = list(
    outcome = "DCR",
    studies = c("Strickler 2023", "Li 2025", "Dilly 2024"),
    file    = "fig5_DCR.png",
    label   = "Disease control rate, KRAS G12C inhibitors"),
  TRAE = list(
    outcome = "TRAE_ge3",
    studies = c("Strickler 2023", "Li 2025", "Yamamoto 2025"),
    file    = "fig7_TRAE.png",
    label   = "Grade >=3 treatment-related adverse events")
)

## Sensitivity pool: ORR with the four-patient D3S-001 PDAC cohort added.
ORR_SENS <- c("Strickler 2023", "Li 2025", "Yamamoto 2025", "D3S-001 2025")

subset_pool <- function(outcome, studies) {
  d <- dat[dat$outcome == outcome & dat$study %in% studies, ]
  d <- d[match(studies, d$study), ]        # fix row order for reproducibility
  stopifnot(nrow(d) == length(studies), !any(is.na(d$events)))
  d
}

fit_pool <- function(d, label) {
  metaprop(
    event            = d$events,
    n                = d$n,
    studlab          = paste0(d$study, " (", d$drug, ")"),
    sm               = "PLOGIT",           # logit-transformed proportion
    method           = "Inverse",          # inverse variance, not GLMM
    method.tau       = "DL",               # DerSimonian-Laird
    random           = TRUE,
    common           = TRUE,
    method.random.ci = "HK",               # Hartung-Knapp adjustment
    title            = label
  )
}

summarise_fit <- function(m, name) {
  bt <- function(x) round(100 / (1 + exp(-x)), 1)
  data.frame(
    pool = name,
    k    = m$k,
    pct  = bt(m$TE.random),
    lci  = bt(m$lower.random),
    uci  = bt(m$upper.random),
    I2   = round(100 * m$I2, 1),
    tau2 = round(m$tau2, 4),
    Q    = round(m$Q, 3),
    df   = m$df.Q,
    Q_p  = round(m$pval.Q, 4)
  )
}

## ---------------------------------------------------------------------------
## Per-study exact (Clopper-Pearson) confidence intervals
##
## These are the intervals quoted for each trial in the Results and drawn on
## the forest plots. They are computed from the events and denominator alone
## and do not depend on the meta-analysis model.
## ---------------------------------------------------------------------------

exact_ci <- function(events, n) {
  ci <- binom.test(events, n)$conf.int
  data.frame(pct = round(100 * events / n, 1),
             lci = round(100 * ci[1], 1),
             uci = round(100 * ci[2], 1))
}

per_study <- cbind(
  dat[, c("study", "ref", "drug", "allele", "outcome", "events", "n")],
  do.call(rbind, Map(exact_ci, dat$events, dat$n))
)
cat("\nPer-study proportions with exact 95% confidence intervals\n")
print(per_study, row.names = FALSE)

## ---------------------------------------------------------------------------
```

```
## Primary pooled estimates and forest plots
## ---------------------------------------------------------------------------

results <- list()
fits    <- list()

for (nm in names(POOLS)) {
  p <- POOLS[[nm]]
  d <- subset_pool(p$outcome, p$studies)
  m <- fit_pool(d, p$label)
  fits[[nm]]    <- m
  results[[nm]] <- summarise_fit(m, nm)

  png(p$file, width = 2200, height = 900, res = 240)
  forest(m,
         pscale       = 1,
         digits       = 3,
         leftcols     = c("studlab", "event", "n"),
         leftlabs     = c("Study (drug)", "Events", "N"),
         print.I2     = TRUE,
         print.tau2   = TRUE,
         print.pval.Q = TRUE,
         col.square   = "grey35",
         col.diamond  = "black",
         smlab        = p$label)
  dev.off()
}

## ---------------------------------------------------------------------------
## Leave-one-out sensitivity analyses (ORR and DCR)
##
## At k = 3 every leave-one-out pool has k = 2, where the Hartung-Knapp
## interval is uninformative. The point estimates, not the intervals, are the
## quantity of interest here, and the manuscript says so explicitly.
## ---------------------------------------------------------------------------

loo_table <- function(nm) {
  p <- POOLS[[nm]]
  do.call(rbind, lapply(p$studies, function(s) {
    keep <- setdiff(p$studies, s)
    m    <- fit_pool(subset_pool(p$outcome, keep), p$label)
    summarise_fit(m, paste0(nm, ", omitting ", s))
  }))
}

loo_ORR <- loo_table("ORR")
loo_DCR <- loo_table("DCR")

png("fig6_LOO_DCR.png", width = 2200, height = 900, res = 240)
forest(metainf(fits$DCR, pooled = "random"),
       smlab = "Leave-one-out, disease control rate")
dev.off()

## ---------------------------------------------------------------------------
## Post-hoc ORR sensitivity analysis adding the D3S-001 PDAC cohort
## ---------------------------------------------------------------------------

orr_sens <- summarise_fit(
  fit_pool(subset_pool("ORR", ORR_SENS), "ORR, sensitivity"),
  "ORR sensitivity (+ D3S-001, post-hoc)")

## ---------------------------------------------------------------------------
## Safety sensitivity analyses: the glecirasib denominator
##
## The glecirasib grade >=3 TRAE denominator (n = 54) is the broader KRAS G12C
## solid-tumour safety population reported by that trial, not the 32-patient
## PDAC efficacy subset, and it carries the largest weight in the pool. Two
## analyses quantify the consequence: (a) rescaling the glecirasib event count
## to the 32-patient PDAC subset at the observed rate, and (b) removing the
## glecirasib dataset altogether.
## ---------------------------------------------------------------------------

d_trae <- subset_pool("TRAE_ge3", POOLS$TRAE$studies)

d_rescaled <- d_trae
i <- which(d_rescaled$study == "Li 2025")
rate <- d_rescaled$events[i] / d_rescaled$n[i]
d_rescaled$events[i] <- round(rate * 32)
d_rescaled$n[i]      <- 32
```

```
trae_sens_rescaled <- summarise_fit(
  fit_pool(d_rescaled, "TRAE, glecirasib rescaled to the PDAC subset"),
  "TRAE sensitivity (glecirasib rescaled to n = 32)")

trae_sens_dropped <- summarise_fit(
  fit_pool(d_trae[d_trae$study != "Li 2025", ], "TRAE, glecirasib removed"),
  "TRAE sensitivity (glecirasib removed, k = 2)")

## ---------------------------------------------------------------------------
## Results table
## ---------------------------------------------------------------------------

final <- do.call(rbind, c(results,
                          list(orr_sens),
                          list(trae_sens_rescaled),
                          list(trae_sens_dropped),
                          list(loo_ORR),
                          list(loo_DCR)))
rownames(final) <- NULL
print(final, row.names = FALSE)
write.csv(final, "S4_reproduced_results.csv", row.names = FALSE)

## ---------------------------------------------------------------------------
## Values reported in the manuscript, for verification against `final`
##
##   ORR   k=3   36.9%  (9.8 - 75.7)   I2 = 66.2%  tau2 = 0.302
##                                     Q = 5.924  df = 2  p = 0.052
##   DCR   k=3   85.9%  (62.1 - 95.8)  I2 = 0%     tau2 = 0
##                                     Q = 1.912  df = 2  p = 0.384
##   TRAE  k=3   23.8%  (11.2 - 43.5)  I2 = 0%     tau2 = 0
##                                     Q = 1.804  df = 2  p = 0.406
##
##   ORR leave-one-out range           31.8% - 46.3%
##     omitting Strickler 2023         46.3%  (37.6 - 55.2)   I2 = 0%
##   DCR leave-one-out range           83.3% - 88.4%
##     omitting Dilly 2024             88.4%                  I2 = 31.6%
##
##   ORR sensitivity (+ D3S-001) k=4   40.3%  (15.6 - 71.2)   I2 = 61.7%
##   TRAE glecirasib rescaled    k=3   22.9%  (10.1 - 43.8)   I2 = 0%
##   TRAE glecirasib removed     k=2   19.7%  (interval uninformative at k = 2)
##
##   Per-study exact 95% CIs
##     ORR   Strickler  8/38   21.1%  (9.6 - 37.3)
##           Li        15/32   46.9%  (29.1 - 65.3)
##           Yamamoto  10/22   45.5%  (24.4 - 67.8)
##           Park       6/31   19.4%  (7.5 - 37.5)    not pooled
##           D3S-001    3/4    75.0%  (19.4 - 99.4)   sensitivity only
##     DCR   Strickler 32/38   84.2%  (68.7 - 94.0)
##           Li        30/32   93.8%  (79.2 - 99.2)
##           Dilly     18/22   81.8%  (59.7 - 94.8)
##     TRAE  Strickler  6/38   15.8%  (6.0 - 31.3)
##           Li        15/54   27.8%  (16.5 - 41.6)
##           Yamamoto   6/24   25.0%  (9.8 - 46.7)
##
## Note on denominators. The glecirasib grade >=3 TRAE denominator (n = 54) is
## the broader KRAS G12C solid-tumour safety population reported by that trial,
## not the 32-patient PDAC efficacy subset. This is stated in the manuscript,
## is the reason the safety pool is rated indirect in the GRADE table
## (Supplementary File S3), and is quantified by the two sensitivity analyses
## above.
###############################################################################
```
