# Supplementary material for: Allele-Stratified Activity of RAS Inhibitors in Pancreatic Ductal Adenocarcinoma: A Systematic Review and Exploratory Meta-Analysis
Source: Cancers (Basel). 2026 Sep 28;18(19):3139. doi: 10.3390/cancers18193139 (PMC13664068; doi:10.3390/cancers18193139)
Supplement: Supplementary file 1 [file cancers-18-03139-s001.zip › Supplementary_File S5_Model_Validation_and_Achievability.pdf]

Supplementary File S5

Model-validation classification of the experimental evidence and pharmacological-achievability appraisal of the in vitro concentrations

Manuscript: Allele-stratified activity of RAS inhibitors in pancreatic ductal adenocarcinoma (cancers-4500792). This file supports Table 5, Table 6 and the Discussion subsection “Pharmacological Achievability of the In Vitro Concentrations”.

1. Classification of experimental model validation

Each of the 20 included experimental studies was classified, independently and in duplicate, by the highest level of model validation it achieved, using the hierarchy given in the Methods. This classification was added in response to peer review and is a post-hoc analysis; the hierarchy was specified in full before any study was classified and applied uniformly to all 20 studies. A second, independent judgement recorded whether the principal finding of the study was reproduced across at least two independent classes of model system. Disagreements were resolved by discussion with a third reviewer. Tier codes: T1 conventional two-dimensional cell lines only; T2 cell lines with three-dimensional spheroid culture; T3 patient-derived material in vitro without an in vivo component; T4 cell-line-derived xenograft, syngeneic or orthotopic in vivo without patient-derived material; T5 patient-derived xenograft; T6 multi-model validation (≥3 complementary systems including both patient-derived material and an in vivo model).

Study [ref]	Model systems reported	Tier	Patient-derived material	In vivo	Immuno-competent	Principal finding reproduced across model classes
Abuelafia 2025 [21]	MiaPaCa-2, Panc-1, BxPC-3 cell lines; PDAC001T-PDAC064T patient-derived primary cultures	T3	Yes	No	No	Partly — patient-derived cultures but no in vivo confirmation
Dilly 2024 [22]	Cell lines; patient-derived organoids; PDX; KPC genetically engineered mice; patient cohort	T6	Yes	Yes	Yes	Yes
Ghanwatkar 2025 [23]	PANC-1, AsPC-1; murine KPC/Y and UN-KPC-961 lines; in vivo tumour-burden experiments	T4	No	Yes	Yes	Yes
Hallin 2022 [24]	KRAS G12D cell lines in 2-D and 3-D; cell-line-derived xenografts; patient-derived xenografts	T5	Yes	Yes	No	Yes
Han 2024 [25]	PANC-1, SW1990, Panc04.03, HPAC; mouse xenograft models	T4	No	Yes	No	Yes
Jesenko 2025 [26]	MIA PaCa-2, PANC-1 monolayer culture only	T1	No	No	No	No
Jiang 2025 [27]	Patient-derived organoids; patient-derived xenografts; KC genetically engineered mice	T6	Yes	Yes	Yes	Yes
Kumarasamy 2024 [28]	PDAC cell lines; spheroids; patient-derived xenografts; syngeneic mice	T6	Yes	Yes	Yes	Yes
Ma 2022 [30]	BxPC-3, AsPC-1, MIA PaCa-2, Capan-1, COLO357, T3M4, PATU8902 monolayer culture only	T1	No	No	No	No
Mahadevan 2023 [31]	Organoids; patient-derived xenografts; orthotopic and immunocompetent mouse models	T6	Yes	Yes	Yes	Yes
Miyazaki 2024 [32]	MIA PaCa-2, SUIT-2, KP-4, PANC-1, PK-59; xenografts	T4	No	Yes	No	Yes
Molnár 2024 [33]	PANC-1, AsPC-1, SW1990 in 2-D and 3-D spheroid culture; no in vivo component	T2	No	No	No	Partly — 3-D confirmation only
Ozawa 2025 [34]	Cell lines; patient-derived organoids; patient-derived xenografts; human PDAC tumour specimens	T6	Yes	Yes	No	Yes
Paulsohn 2025 [36]	MIA PaCa-2, 51T-2D, AsPC-1; patient-derived organoids; GöCDX models; KPC models	T6	Yes	Yes	Yes	Yes
Principe 2026 [37]	PANC-1, AsPC-1, CD18 and murine KPC-2138, KPC-3213, FC1245 lines; 3-D PANC1K cultures; in vivo survival experiments	T4	No	Yes	Yes	Yes
Ramalingam 2025 [38]	MIA PaCa-2, PANC-1 monolayer culture only	T1	No	No	No	No
Song 2024 [39]	MIA PaCa-2, Calu1, resistant derivatives; patient-derived organoids; mouse models	T6	Yes	Yes	No	Yes
Szigeti 2026 [41]	ASPC1, SW1990, PANC1; in vivo liver-metastasis model	T4	No	Yes	No	Yes

Tajiknia 2024 [42]	BxPC-3, AsPC-1, MIA PaCa-2 and CRC lines, monolayer culture only	T1	No	No	No	No
Xu 2026 [43]	AsPC-1, PANC-1, SW1990, murine KPC1199; xenografts	T4	No	Yes	No	Yes

1.1 Summary of the classification

Category	n / 20	%	Studies
Conventional two-dimensional cell lines only (T1)	4	20	[26,30,38,42]
Cell lines with three-dimensional spheroid culture (T2)	1	5	[33]
Patient-derived material in vitro, no in vivo (T3)	1	5	[21]
Xenograft / syngeneic / orthotopic in vivo, no patient-derived material (T4)	6	30	[23,25,32,37,41,43]
Patient-derived xenograft (T5)	1	5	[24]
Multi-model validation (T6)	7	35	[22,27,28,31,34,36,39]
Any in vivo component	14	70	[22,23,24,25,27,28,31,32,34,36,37,39,41,43]
Any patient-derived material	9	45	[21,22,24,27,28,31,34,36,39]
Immunocompetent (GEMM / syngeneic / orthotopic) models	7	35	[22,23,27,28,31,36,37]
Principal finding reproduced across ≥2 model classes	14	70	[22,23,24,25,27,28,31,32,34,36,37,39,41,43]

2. Pharmacological achievability of the in vitro concentrations

Every quantitative in vitro potency value reported in Table 9 and Table 10 of the main text was compared with the systemic exposure achievable in patients at the licensed or clinically evaluated dose of the same agent. Exposure benchmarks are derived from regulatory pharmacokinetic data; plasma protein binding is stated because only the unbound fraction is pharmacologically available. Where the compound tested has no human pharmacokinetic data, achievability is recorded as not assessable rather than assumed.

2.1 Clinical exposure benchmarks

Agent	Clinical dose	Exposure benchmark	Plasma protein binding	Source
Sotorasib (AMG-510)	960 mg once daily (licensed)	Steady-state apparent clearance 26.2 L/h; average steady-state total plasma concentration ~1.5 µg/mL (~2.7 µM, molecular weight 560.6); unbound ~0.3 µM	89%	US prescribing information [58]
Adagrasib (MRTX849)	600 mg twice daily (licensed)	Steady state reached within 8 days with ~6-fold accumulation; unbound fraction is a small proportion of total exposure	98%	US prescribing information [59]
MRTX1133	No approved or reported human dose	Not orally bioavailable; administered parenterally in all source studies. No human pharmacokinetic data published. A first-in-human study has opened but has reported no exposure data.	Not reported in humans	Wang et al. 2022 [57]
BI-3406 (KRAS::SOS1)	No clinical use in PDAC	Not assessable	Not reported	-

2.2 Appraisal of the reported in vitro concentrations

Agent / value [ref]	Model	Concentration	Comparison with achievable exposure	Classification
Sotorasib IC50 0.04 µM [38]	MIA PaCa-2	40 nM	Approximately 1/70 of average total steady-state exposure; below unbound exposure	Plausibly achievable
Sotorasib IC50 0.25 µM [26]	MIA PaCa-2	250 nM	Approximately 1/10 of average total steady-state exposure; near unbound exposure	Plausibly achievable
Sotorasib IC50 2.5 µM [36]	MIA PaCa-2	2.5 µM	Comparable to average total steady-state exposure but ~8-fold above unbound exposure	Uncertain achievability
Sotorasib IC50 20 µM [38]	PANC-1 (non-G12C)	20 µM	~7-fold above average total steady-state exposure; ~70-fold above unbound	Above achievable exposure
Sotorasib and BI-3406 comparisons at 10 µM [30]	Multiple lines, cross-allele comparison	10 µM	~4-fold above average total steady-state exposure; ~30-fold above unbound; BI-3406 has no clinical benchmark	Above achievable exposure
Adagrasib IC50 0.13 µM [39]	MIA PaCa-2	130 nM	Within the range of reported clinical exposure on total drug; unbound fraction 2%	Uncertain achievability
Adagrasib IC50 60.2 µM [39]	MIA PaCa-2R (resistant)	60.2 µM	Far above any achievable exposure	Above achievable exposure; qualitative demonstration of resistance only
MRTX1133 IC50 5-50 nM [24,31,33,41,43]	G12D-mutant lines, 2-D and 3-D	5-50 nM	No human pharmacokinetic data exist for this compound	Not assessable
MRTX1133 IC50 1.77-2.5 µM [42]	HPAF-II, PANC-1	1.8-2.5 µM	No human pharmacokinetic data exist for this compound	Not assessable
MRTX1133 IC50 >1 µM to >10 µM [28,33,34,42]	Resistant and wild-type lines	>1 µM	No human pharmacokinetic data exist for this compound	Not assessable
MDP5 IC50 18.06 µM [23]	AsPC-1	18.06 µM	Investigational conjugate; no clinical benchmark	Not assessable
M1C ADC IC50 31 nM [34]	PF405	31 nM	Investigational antibody-drug conjugate; no clinical benchmark	Not assessable

Interpretation. Of the values appraised, two sotorasib potency estimates fall within plausibly achievable exposure, two are of uncertain achievability, and the cross-allele comparisons made at 10 µM and the values above 10 µM lie above achievable exposure and should be read as pharmacology at supratherapeutic concentrations. The single largest category is not assessable, and it is occupied almost entirely by MRTX1133, the compound underpinning the majority of the KRAS G12D preclinical literature synthesised in this review. This does not diminish the mechanistic value of those studies, but it does mean that no statement about the clinical relevance of the concentrations used in them can be made, and that the synergy observed in MRTX1133 combination experiments cannot be translated into a prediction about combination therapy in patients.
